# Graphitic Carbon Nitride (g-C_3_N_4_)-Catalysed Green Synthesis of Heterocycles: Progress, Mechanistic Insights, and Future Perspectives

**DOI:** 10.3390/molecules31183188

**Published:** 2026-09-10

**Authors:** Jayanthi Barasarathi, Kasi Venkatesan, Saleh Alofi, Malgorzata Jeleń, Parasuraman Karthikeyan, Potchanun Sripothong, Beata Morak Młodawska

**Affiliations:** 1 Faculty of Health and Life Sciences, INTI International University, Persiaran Perdana BBN, Putra Nilai, Nilai 71800, Negeri Sembilan, Malaysia; jayanthi.baras@newinti.edu.my; 2 Chemistry Division, Department of H&S, CVR College of Engineering, Vastunagar, Mangalpalli (V), R.R Dist, Hyderabad 501510, Telangana, India; 3 Department of Chemistry, College of Science in Yanbu, Taibah University, Yanbu Governorate, Saudi Arabia; 4 Department of Organic Chemistry, Faculty of Pharmaceutical Sciences, The Medical University of Silesia, Jagiellońska 4, 41-200 Sosnowiec, Poland; manowak@sum.edu.pl; 5 PG and Research Department of Chemistry, Pachaiyappas College Campus, University of Madras, Chennai 600030, Tamil Nadu, India; 6 Faculty of Engineering and Technology, Shinawatra University, Pathum Thani 12160, Thailand

**Keywords:** graphitic carbon nitride (g-C_3_N_4_), heterocyclic compounds, visible-light catalysis, sustainable synthesis, multicomponent reactions (MCRs), catalyst recyclability, green chemistry

## Abstract

Graphitic carbon nitride (g-C_3_N_4_) has proved to be an excellent and versatile catalyst, devoid of any metal, for the sustainable production of heterocycles. Owing to its nitrogen-rich conjugated framework, which provides Lewis-basic and hydrogen-bonding sites and enables tuneable electronic properties, together with its excellent thermal and chemical stability and recyclability, graphitic carbon nitride (g-C_3_N_4_) has emerged as a versatile metal-free heterogeneous catalyst for the synthesis of heterocycles. In this review, a critical analysis of recent trends in g-C_3_N_4_-catalysed multicomponent reactions (MCRs) and their related approaches towards the synthesis of pharmaceutically important heterocycles is provided. This review highlights current developments in g-C_3_N_4_-catalysed multicomponent reactions (MCRs) for the synthesis of biologically and pharmaceutically relevant heterocyclic frameworks, including pyrimidines, pyridines, pyrans, chromenes, tetrazoles, quinolines, imidazoles, triazoles, and spirocyclic heterocycles. Mechanistic aspects, including Lewis acid–base mechanism, photocatalysis and formation of radicals, have been highlighted to establish the correlation between catalyst properties and the reaction outcomes. The sustainability of the methodology is assessed through reaction mass efficiency, atom economy, process mass intensity, E-factor, energy efficiency and catalyst reusability. Critically analysed advancements have been made regarding the development of hybrid catalysts based on g-C_3_N_4_. Challenges faced during catalyst deactivation, utilization of visible light, scalability and incompleteness in green metric reporting are addressed.

## 1. Introduction

Carbon is one of the most multifaceted elements in the periodic table and displays great structural and chemical versatility. Carbon-containing materials have received considerable interest from many scientific and technological areas due to their unique physicochemical properties [1,2]. In recent decades, there have been many efforts aimed at the design and development of new carbon nanomaterials for many applications. In contrast to bulk materials, carbon nanomaterials at nanoscale display a range of exceptional features such as low density, high thermal and electrical conductivity, high mechanical strength and good chemical stability [3]. Moreover, the broad range of carbon nanostructures allows one to adjust their properties according to specific application conditions.

**Figure 1:**
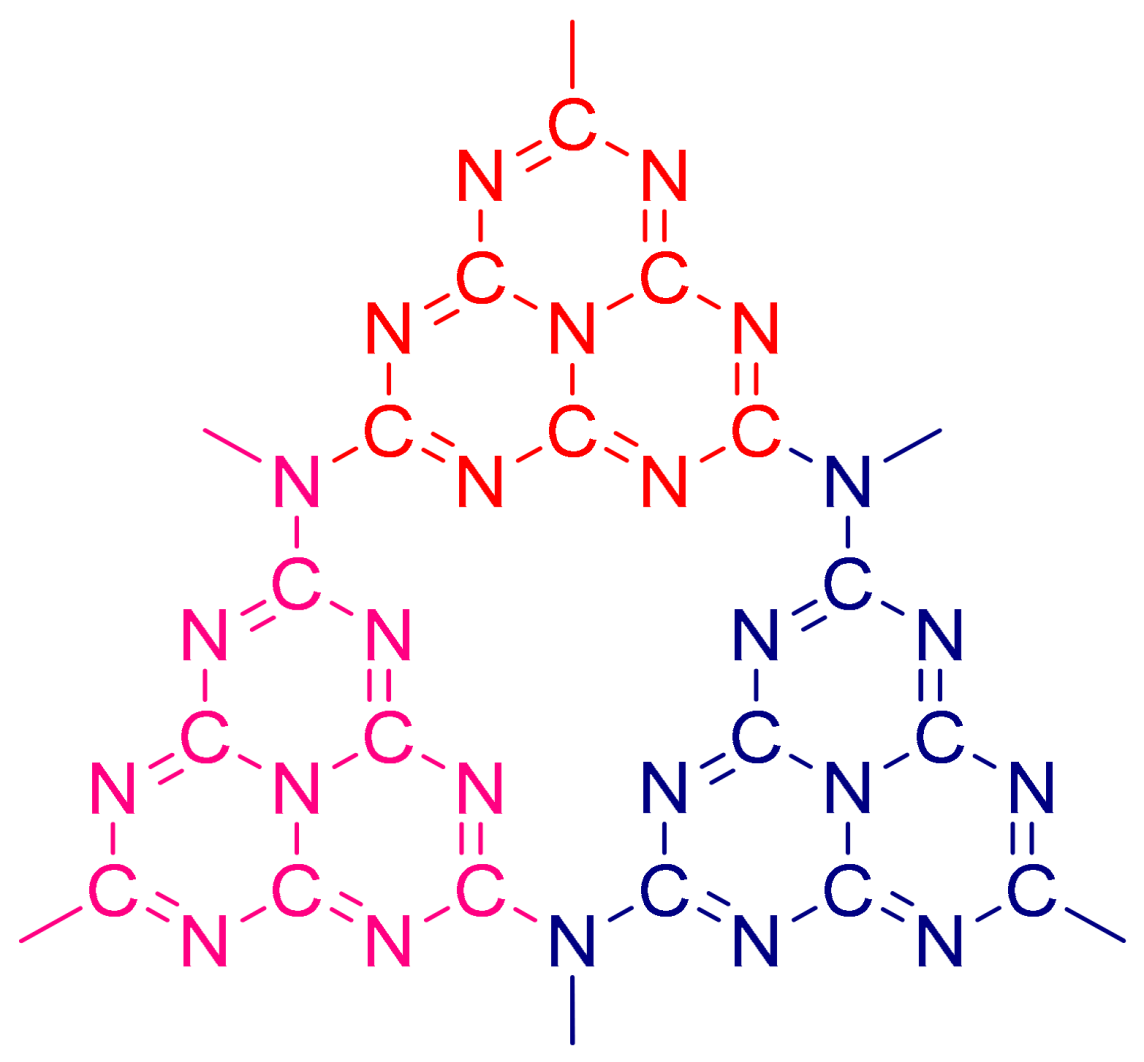
2D structure of g-C_3_N_4_.

Thus, carbon nanomaterials have found many applications in optoelectronics, photonics, nanomedicine, biotechnology, energy storage devices, polymer composites and catalysis [4,5,6]. The unique property of carbon that allows it to form various allotropes and nanostructures with different physical and chemical properties forms the basis of its great importance for modern materials science [5]. In terms of carbon-containing substances, a great deal of focus has been given to g-C_3_N_4_, a metal-free and stable catalyst for organic reactions. Due to its abundance on Earth, non-toxic nature, and good stability to heat and chemicals, g-C_3_N_4_ can easily be synthesized from nitrogen sources through a single-step method and the structure is given in Figure 1 [7].

Graphitic carbon nitride (g-C_3_N_4_) has been demonstrated to be a highly favourable non-metallic semiconductor polymer based on its outstanding physicochemical features, environmental compatibility, and catalytic properties. Although g-C_3_N_4_ is attractive because it can be prepared from readily available carbon- and nitrogen-containing precursors and does not require a metal component, its overall economic and environmental benefits should not be generalized without considering the synthesis route, energy requirements, precursor cost, scalability, and catalyst recovery. Thus, g-C_3_N_4_ is better regarded as a potentially sustainable and versatile photocatalytic platform rather than being universally superior in cost or environmental performance to established semiconductor materials such as TiO_2_. Following the discovery of visible-light-facilitated hydrogen production through polymeric carbon nitride by Xinchen Wang and his group in 2009, investigations into g-C_3_N_4_ have flourished in the fields of photocatalysis, electrocatalysis, environmental pollution removal, energy harvesting, and chemical transformations [8]. Recently, g-C_3_N_4_ has received considerable interest owing to its application in recyclable heterogeneous catalysts for synthesizing heterocycles in an economical manner because it integrates both metal-free catalysis and thermal stability with surface basicity and easy recoverability [9].

The interest in g-C_3_N_4_ for heterogeneous organic synthesis arises from the combination of a nitrogen-rich conjugated framework, accessible surface sites, tuneable electronic properties, and the possibility of metal-free or hybrid catalytic architectures. Its heterogeneous nature can also facilitate catalyst separation and reuse in suitable reaction systems. However, the catalytic performance of g-C_3_N_4_ is strongly dependent on its structural form, surface area, defect density, functionalization, reaction medium, and the nature of the substrates. Therefore, its advantages over conventional catalysts should be evaluated on a reaction-specific basis rather than considered universal. In addition, the catalyst has many nitrogen-based sites that help to activate carbonyl compounds, enable hydrogen bonding, and take part in multicomponent reactions that lead to the formation of heterocyclic molecules [10,11].

The formation of heterocycles lies at the heart of organic synthesis because of their presence in almost all drugs, biologically active natural products, agrochemicals, materials, dyes, and advanced electronic materials. Heterocycles serve as an integral element in giving rise to varied biological and physical chemical properties that make them an essential part of medicinal chemistry. Traditional procedures of heterocycle formation generally use catalysts in the form of acid and bases, transition metal compounds, organic solvents and energy-consuming reactions. Even though they give good yield most of the time, these methods have certain disadvantages such as low catalyst reusability, atom wastage, difficulty in purification, metal contamination and formation of toxic waste. Therefore, designing green methods of catalysis that reduce environmental pollution without compromising the catalytic efficiency has been one of the goals of modern synthetic chemistry [12,13,14,15].

Graphitic carbon nitride (g-C_3_N_4_) has attracted considerable interest in heterocyclic synthesis because its nitrogen-rich, metal-free framework provides surface active sites capable of interacting with and activating organic substrates. The presence of Lewis-basic nitrogen sites, surface amino groups, hydrogen-bonding functionalities, and a conjugated structure enables g-C_3_N_4_ to participate in substrate adsorption and catalytic activation, making it suitable for multicomponent and one-pot heterocycle synthesis. In addition, its semiconductor characteristics enable visible-light-driven photocatalytic transformations, providing opportunities to combine conventional surface catalysis with photochemical activation.

Owing to the ready availability and low cost of carbon- and nitrogen-containing precursors, g-C_3_N_4_ can be readily prepared by thermal condensation or polymerization of suitable molecular precursors. The special π-conjugated structure of g-C_3_N_4_ containing Lewis-basic sites as well as the electronically tuneable nature of this material renders it suitable for catalysis in different types of reactions. In addition, the heterogeneous nature of this material helps in the ease of isolation and recycling of the catalyst. It has recently been shown that pristine as well as functionalized forms of g-C_3_N_4_ act as very effective catalysts in the preparation of various heterocycles under mild conditions [16,17,18].

It is important, however, to distinguish pristine g-C_3_N_4_ from functionalized and composite g-C_3_N_4_-based catalysts. In modified systems, the observed catalytic activity may arise from the synergistic contribution of g-C_3_N_4_ and the incorporated functional component. For example, CSA@g-C_3_N_4_ represents an acidic hybrid catalyst in which camphorsulfonic acid is immobilized on the g-C_3_N_4_ surface, introducing additional acid sites for substrate activation. Similarly, KGCN/RGO is a functionalized nanocomposite in which reduced graphene oxide can modify the physicochemical and charge-transfer properties of g-C_3_N_4_. Therefore, these materials should not be considered equivalent to pristine g-C_3_N_4_, and their catalytic performance should be interpreted in terms of the specific interactions and synergistic effects between the constituent components. This distinction is essential for understanding the catalytic mechanisms and structure–activity relationships of g-C_3_N_4_-based systems in sustainable heterocycle synthesis.

The application of g-C_3_N_4_ in one-pot synthesis through MCRs is the most important application that is environmentally friendly as compared to other synthetic methods [19,20,21]. This is because MCRs involve the reaction of three or more reactants at once to form molecules having complexity, good atom economy, bonding efficiency, and few purifications processes thus leading to low use of solvents and hence being eco-friendly. Due to the special physical and chemical characteristics, the metal-free and recyclable nature of g-C_3_N_4_, it is the best catalyst in heterocyclization reactions [22,23].

## 2. Synthesis of Graphitic Carbon Nitride

Graphitic carbon nitride (g-C_3_N_4_) is a non-metallic polymeric semiconductor that has received a lot of prominence owing to its facile preparation process, low cost, outstanding chemical and thermal stability, as well as adjustable electronic features. The common way to prepare g-C_3_N_4_ is the thermal polycondensation of nitrogenous precursors, including melamine, dicyandiamide, cyanamide, urea, thiourea, and ammonium thiocyanate at the temperature range of 500–650 °C with the ambient or inert atmosphere. In the thermal process, nitrogen-containing precursors react through step-by-step deamination and polycondensation reactions, leading to the construction of the intermediate products such as melam, melem, and melon, followed by the polymerization of them into the two-dimensional graphitic structure with tri-s-triazine (heptazine) units interconnected by tertiary nitrogen atoms. The properties of prepared g-C_3_N_4_ are highly dependent on the type of used precursors, calcination temperature, heating rate, and dwelling time [24,25].

However, to circumvent the drawbacks associated with bulk g-C_3_N_4_ such as low surface area and fast recombination of the photogenerated carriers, a number of fabrication methodologies have been used including template-assisted synthesis, supramolecular self-assembly, hydro-/solvothermal treatment, molten salt synthesis, microwave-assisted synthesis, and exfoliation processes to synthesize porous or ultrathin nanosheets. In addition, doping with heteroatoms like B, P, S, O, and halogen atoms, metal incorporation, and formation of heterojunctions with metal oxides, metal sulphides, carbon nanostructures, and metal–organic framework materials have been widely applied to increase the visible light absorption capacity, density of catalytically active sites and charge separation efficiency. These techniques greatly expand the catalytic and photocatalytic activity of g-C_3_N_4_ for organic synthesis, environmental applications, hydrogen generation, CO_2_ reduction, and energy conversion processes, making it one of the most versatile sustainable catalysts in materials science [26,27].

A quantitative comparison with representative semiconductor photocatalysts is useful for positioning g-C_3_N_4_ within the broader photocatalytic materials landscape. Its photocatalytic performance is governed by its optical band gap, band-edge positions, surface area, defect structure, surface functional groups and charge-carrier dynamics. Unlike a single intrinsic descriptor, these properties can vary substantially with synthesis conditions, morphology, doping and defect concentration. In particular, the band gap and CB/VB positions determine the accessible photoinduced redox processes, whereas surface area and surface chemical functionality influence substrate adsorption and interfacial reactions. The nitrogen-rich framework additionally provides Lewis-basic and hydrogen-bonding sites that can be relevant to non-photocatalytic transformations. Accordingly, g-C_3_N_4_ should not be regarded as universally superior to other semiconductor photocatalysts; rather, its value arises from the combination of visible-light responsiveness, tuneable electronic structure, metal-free composition and chemically functional surfaces.

## 3. Surface Chemistry and Catalytic Active Sites of g-C_3_N_4_

Graphitic carbon nitride (g-C_3_N_4_) exhibits special surface chemistry that is responsible for remarkable catalytic activity during various organic reactions. Chemically, g-C_3_N_4_ is made up of 2D layers of conjugated heptazine (tris-triazine) rings bridged together via tertiary nitrogen atoms. The surface of g-C_3_N_4_ is characterized by the presence of different nitrogen functionalities, among which there are pyridinic nitrogen, tertiary (bridging) nitrogen, amino groups (-NH_2_, -NH), and graphitic nitrogen. The nitrogen-rich framework of g-C_3_N_4_ contains Lewis-basic sites that can participate in hydrogen-bonding interactions and, where appropriate, facilitate deprotonation of acidic reaction partners to generate or enhance nucleophilic species. Activation of electrophilic carbonyl substrates, when observed, is more appropriately associated with hydrogen-bonding interactions, surface polarization, or additional acidic/functional sites present in modified g-C_3_N_4_-based catalysts (aldehydes, ketones, carbonyl derivatives, and activated nitriles). Additionally, terminal amino groups can be involved in hydrogen bonding and proton transfer, thus facilitating substrate adsorption and stabilization of the reaction intermediates. Delocalized π-electron structure also ensures high electron mobility and the existence of active adsorption centres for aromatic compounds via π-π interactions [28,29].

Catalytic activity of g-C_3_N_4_ results from the synergy of surface functional groups, electronic structure, and structural defects. Nitrogen vacancy, carbon vacancy, edge defect, and heteroatoms doping (such as B, P, S, O, and halogen) will create extra unsaturated coordination environment and alter the electronic structure of g-C_3_N_4_, thus providing more catalytically active sites. In addition, the exfoliation into ultrathin nanosheets or the formation of porous structure increases the surface area and provides extra active sites. The presence of both Lewis-basic nitrogen sites and Brønsted acidic amine groups can facilitate cooperative acid–base catalysis and enable g-C_3_N_4_ to effectively promote Knoevenagel condensation, Michael addition, Biginelli reaction, and other multicomponent reactions in mild condition. In addition, due to the high thermal and chemical stability, g-C_3_N_4_ possesses good recyclability without any obvious decrease in activity and thus is a promising and sustainable heterogeneous catalyst for green organic synthesis. The results revealed that the combination of g-C_3_N_4_ with metal nanoparticles or metal oxides not only separates charges but also activates substrate and greatly improves the catalytic performance in the thermal and photo-heterocyclic synthesis; were given in Table 1 [30,31].

1. Pristine g-C_3_N_4_ as a metal-free heterogeneous catalyst, where its nitrogen-rich framework, Lewis-basic sites, surface amino groups, π-conjugated structure, and hydrogen-bonding ability can participate directly in substrate activation. The revised manuscript explicitly discusses the activation of carbonyl compounds through surface hydrogen bonding and the role of basic nitrogen sites in facilitating proton abstraction/nucleophile activation.
2. g-C_3_N_4_ as a photocatalyst, where its semiconductor character and visible-light response are fundamentally different from its role in conventional thermal catalysis. We have emphasized that the photocatalytic contribution should not simply be equated with the acid/base catalytic behaviour of pristine g-C_3_N_4_.
3. g-C_3_N_4_ as a support or platform, particularly in systems such as Fe_3_O_4_@g-C_3_N_4_, metal/g-C_3_N_4_, metal oxide/g-C_3_N_4_ and other composite catalysts. In these cases, we now make it clear that the enhanced activity cannot automatically be attributed exclusively to g-C_3_N_4_. Rather, its contribution may involve highly dispersed active species, improved interfacial contact, adsorption, charge separation, electronic interaction, surface-area effects, and stabilization of the active component.
4. Functionalized g-C_3_N_4_ as a synergistic catalyst, where immobilized acidic/basic functionalities or other catalytic species provide the primary chemical functionality while g-C_3_N_4_ offers a heterogeneous, recyclable and electronically tuneable support. For example, this review now explicitly describes CSA@g-C_3_N_4_ as an acidic hybrid system rather than presenting the activity as originating solely from pristine g-C_3_N_4_.

## 4. Recent Progress in g-C_3_N_4_-Catalysed Heterocyclic Synthesis

In recent years, g-C_3_N_4_ has been identified as a highly efficient and sustainable catalyst for the preparation of different types of heterocyclic compounds. Electron-rich sites of nitrogen, surface amino groups, and conjugation in g-C_3_N_4_ facilitate its functionality towards acidic–basic activation, hydrogen bonding, and photoreactivity. g-C_3_N_4_ has successfully led to the efficient synthesis of different heterocyclic compounds such as chromenes, pyrans, spirooxindoles, imidazopyridines, xanthenes, pyrroles, coumarins, acridines and phenazines.

### 4.1. Synthesis of Pyrimidine and Pyridine Derivatives

Pyrimidine and pyridine derivatives are significant classes of nitrogen-containing heterocyclic compounds that occupy a central position in medicinal chemistry, pharmaceutical development, and functional materials owing to their diverse biological and physicochemical properties [32,33]. The pyridine nucleus is widely present in biologically active molecules and contributes to hydrogen bonding, metal coordination, and modulation of molecular polarity, while the pyrimidine scaffold, containing two ring nitrogen atoms, is a prominent structural feature in nucleic acid bases and numerous therapeutic agents. Both heterocyclic scaffolds exhibit diverse bioactivities, including antiviral, anticancer, anti-inflammatory, antimicrobial, antitubercular, and antioxidant properties [34,35].

**Scheme 1:**
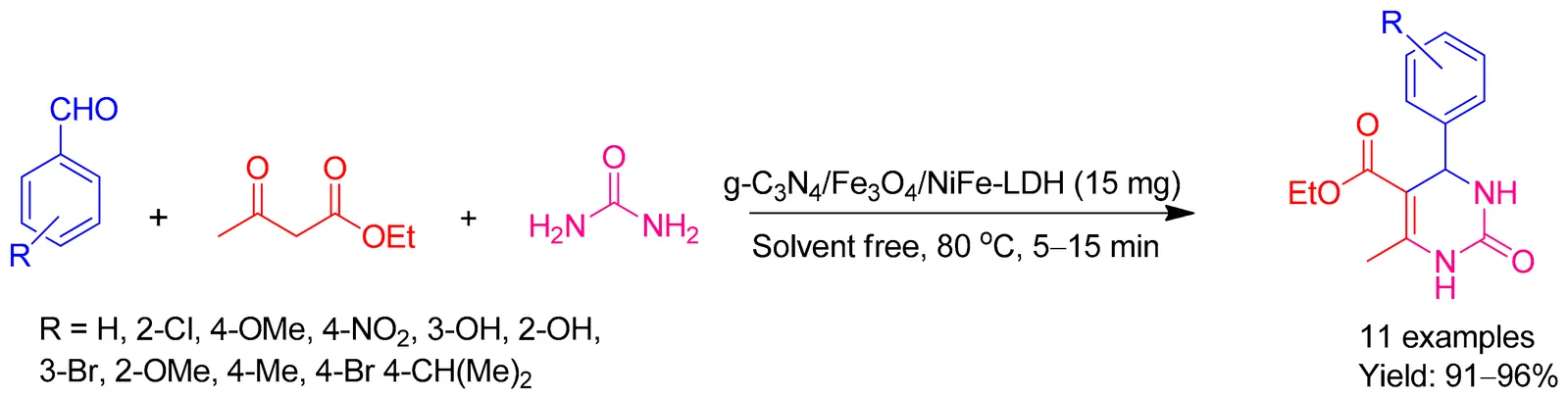
Synthesis of DHPM derivatives using C_3_N_4_/Fe_3_O_4_/NiFe-LDH catalyst.

In 2025, Maryam Gani et al. [36] reported a novel magnetic mesoporous C_3_N_4_/Fe_3_O_4_/NiFe layered double hydroxide (LDH) nanocomposite as a highly efficient heterogeneous catalyst. The catalyst successfully utilized the Biginelli reaction of urea, various benzaldehydes, and ethyl acetoacetate under solvent-free conditions, affording 3,4-DHPM derivatives in 5–15 min with 91–96% yields, as shown in Scheme 1. The catalyst also exhibited excellent recyclability and was reused for five repeated runs with no substantial loss of catalytic action.

**Scheme 2:**
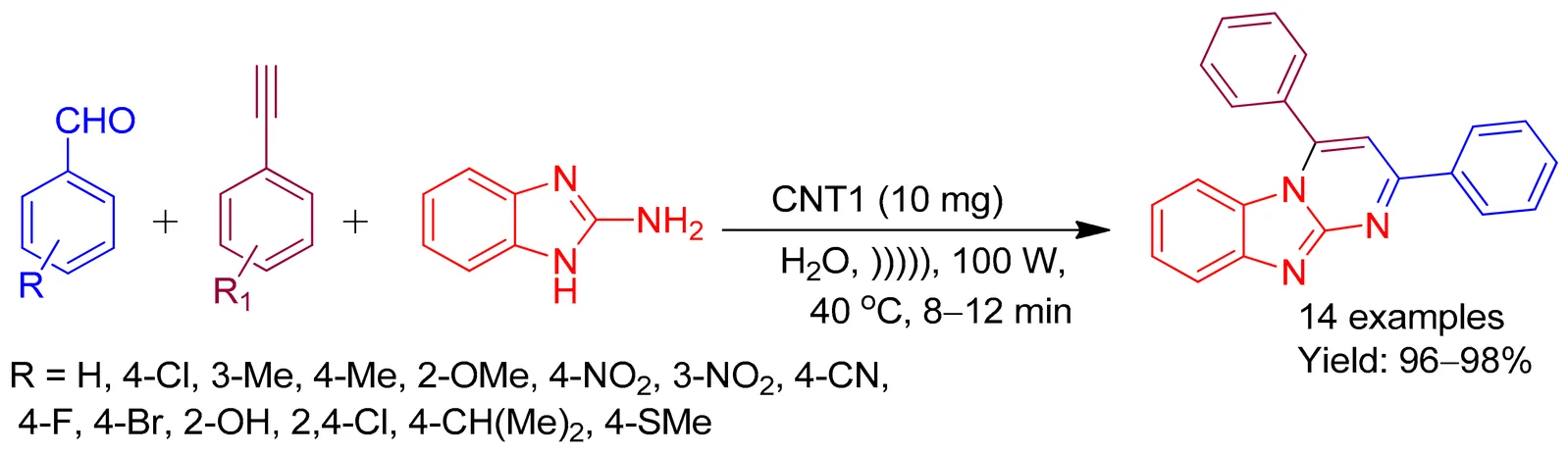
Synthesis of imidazo-pyrimidine derivatives catalysed by PIL-Br-modified hollow tubular g-C_3_N_4_ (CNT1).

In 2024, Javad Safaei Ghomi et al. [37] reported the fabrication of hollow tubular g-C_3_N_4_ using poly(ionic liquid) bromide (PIL-Br) as a template, affording CNT-1 with a unique nanotubular architecture and enhanced catalytic performance. The PIL-Br-modified CNT catalyst efficiently promoted the regiospecific synthesis of pyrimidines via multicomponent reaction of phenylacetylene, 2-aminobenzimidazole, and various aldehydes under ultrasonication, affording the desired products in 3–15 min with an exceptional yield (96–98%), as shown in Scheme 2. The catalyst retained its catalytic efficiency over six repeated cycles.

**Scheme 3:**
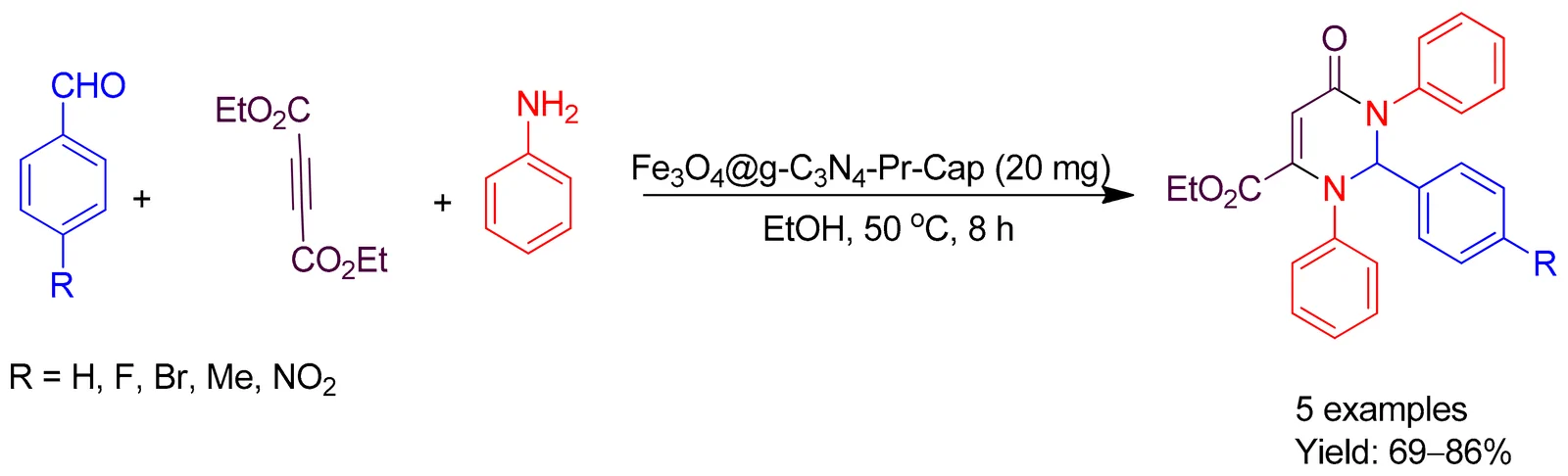
Synthesis of pyrimidine derivatives using Fe_3_O_4_@g-C_3_N_4_-Pr-Cap catalyst.

In 2023, Fatemeh Rezaei et al. [38] reported a captopril-functionalized magnetic graphitic carbon nitride catalyst, prepared by anchoring captopril onto the surface of magnetic g-C_3_N_4_ using a linker agent. The heterogeneous catalyst efficiently used the one-pot preparation of pyrimidine derivatives from various benzaldehydes, diethyl acetylenedicarboxylate, and anilines in ethanol at 50 °C, giving the products within 8 h with 69–86% yields, as shown in Scheme 3. The catalyst showed exceptional recyclability, retaining its catalytic activity over five repeated cycles.

A considerable difference in reaction times is observed among these g-C_3_N_4_-based catalytic systems. The C_3_N_4_/Fe_3_O_4_/NiFe-LDH system completes the Biginelli reaction within 5–15 min under solvent-free conditions, while PIL-Br-modified tubular g-C_3_N_4_ affords products within 3–15 min under ultrasonication. In contrast, Fe_3_O_4_@g-C_3_N_4_-Pr-Cap requires 8 h at 50 °C in ethanol. These differences arise from variations in catalyst architecture, reaction medium, activation mode, and substrate combination; therefore, direct comparison of reaction times should be made cautiously without standardized reaction conditions.

**Scheme 4:**
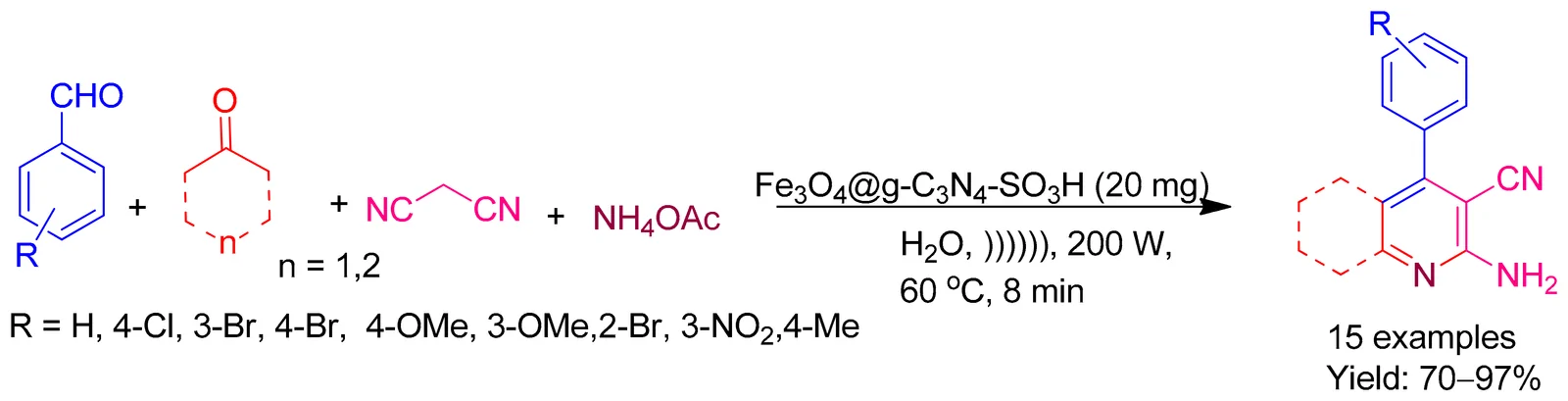
Synthesis of functionalized pyridine derivatives using Fe_3_O_4_@g-C_3_N_4_-SO_3_H catalyst.

In 2020, Edrisi [39] and associates reported Fe_3_O_4_@g-C_3_N_4_–SO_3_H as a powerful and facile heterogeneous catalyst for the one-pot preparation of polysubstituted pyridine derivatives. The catalyst efficiently promoted the reaction of various aldehydes, ammonium acetate, ketones, and malononitrile under ultrasonic irradiation in water, furnishing the required compounds in good to excellent yields (70–97%) within 8 min, as illustrated in Scheme 4. Furthermore, the catalyst was readily recovered and successfully reused for up to five repeated reaction runs without major loss of catalytic action.

**Scheme 5:**
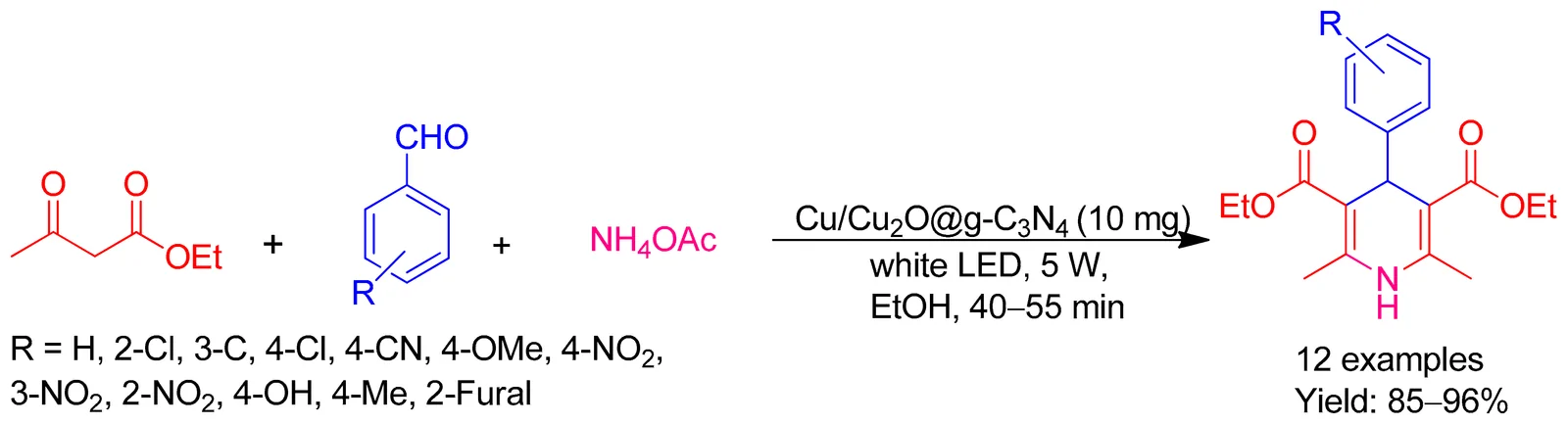
Synthesis of 1,4-DHPs using Cu/Cu_2_O@g-C_3_N_4_ catalyst.

Puneet K. Singh et al. (2023) [40] reported a Cu/Cu_2_O@g-C_3_N_4_ nanocomposite as an effective and reusable photocatalyst for the synthesis of 1,4-DHPs. The reaction involved the one-pot condensation of various aldehydes, NH_4_OAc, and ethyl acetoacetate under white LED irradiation in ethanol as the solvent. A broad range of pyridines was obtained in 40–55 min with excellent yields of 85–96%, as illustrated in Scheme 5. The catalyst was effectively reused for five cycles with negligible loss of activity.

**Scheme 6:**
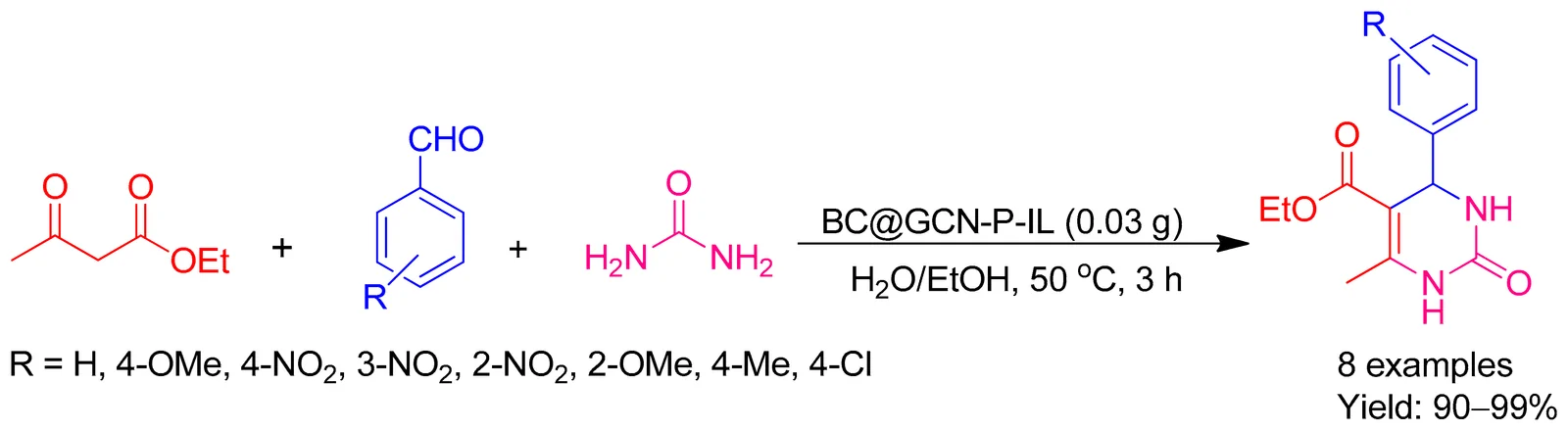
Synthesis of 3,4-DHPMs using BC@GCN-P-IL catalyst.

In 2020, Samahe Sadjadi et al. [41] reported a biochar-based GCN (BC@g-C_3_N_4_) catalyst prepared from the calcination of *Zinnia grandiflora* petals and urea, followed by functionalization with acidic and ionic-liquid groups. The catalyst effectively used a Biginelli reaction of ethyl acetoacetate, urea and various aldehydes in aqueous medium under mild conditions, affording 3,4-DHPMs in 3 h with 90–99% yields, as illustrated in Scheme 6. The catalyst was reused for seven repeated runs without substantial loss of catalytic activity.

**Scheme 7:**
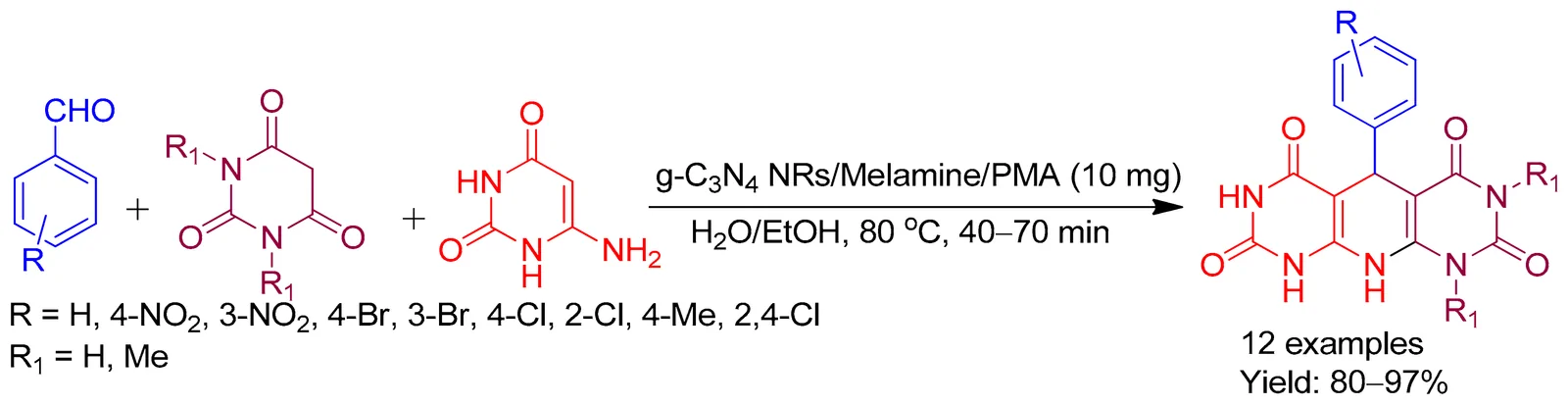
Synthesis of pyrido-dipyrimidine derivative using g-C_3_N_4_ NRs/Melamine/PMA catalyst.

In 2026, Josheghani [42] and co-workers reported phosphomolybdic acid (PMA)-supported GCN nanorods (g-C_3_N_4_ NRs/Melamine/PMA) as a competent catalyst. The catalyst was employed for the one-pot preparation of pyrido-dipyrimidines via the condensation of 6-aminouracil, various aromatic aldehydes and barbituric acid, in aqueous medium, affording the desired products within 120–150 min with 80–97% yields, as illustrated in Scheme 7. The catalyst showed superb recyclability and retained its catalytic action for five succeeding cycles.

**Scheme 8:**
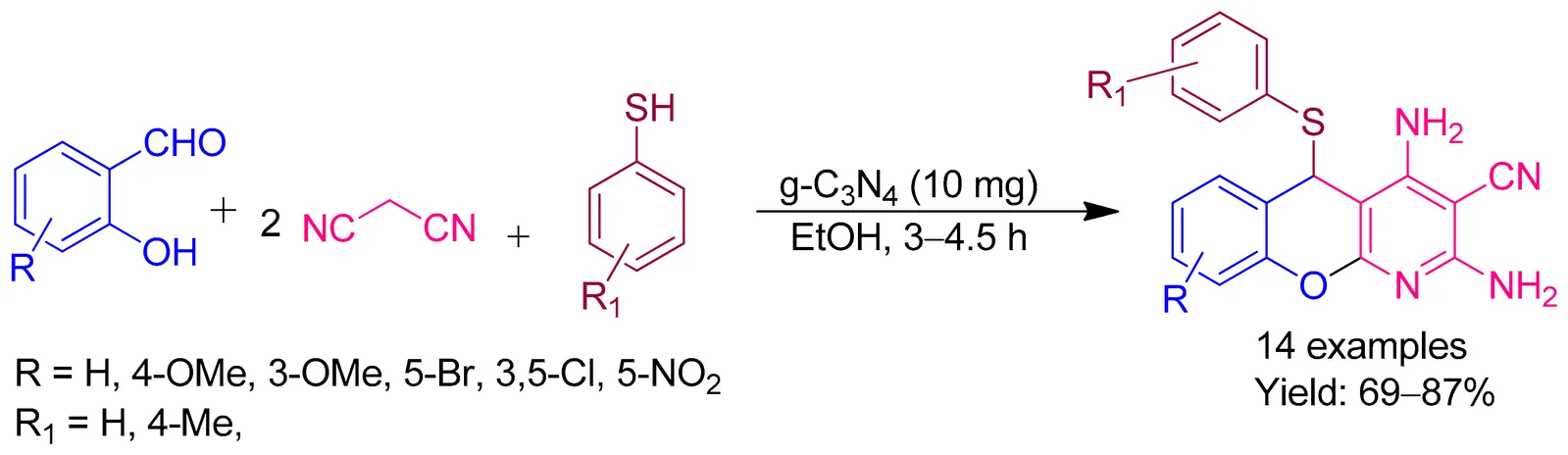
Synthesis of chromeno-pyridine derivatives using g-C_3_N_4_ catalyst.

In 2022, Sushmita Gajurel [43] and co-workers reported the synthesis of g-C_3_N_4_ via the simple thermal polymerization of inexpensive and readily available urea. The catalytic performance of the prepared g-C_3_N_4_ was investigated in the one-pot 3CR of chromeno-pyridines by reacting salicylaldehyde, malononitrile, and thiophenol in ethanol. The procedure provided the desired compounds in excellent yields of 69–87% within 3–4.5 h under mild reaction conditions. Furthermore, the heterogeneous catalyst showed excellent recyclability, retaining its catalytic activity over five succeeding cycles. The synthetic methodology is illustrated in Scheme 8.

**Scheme 9:**
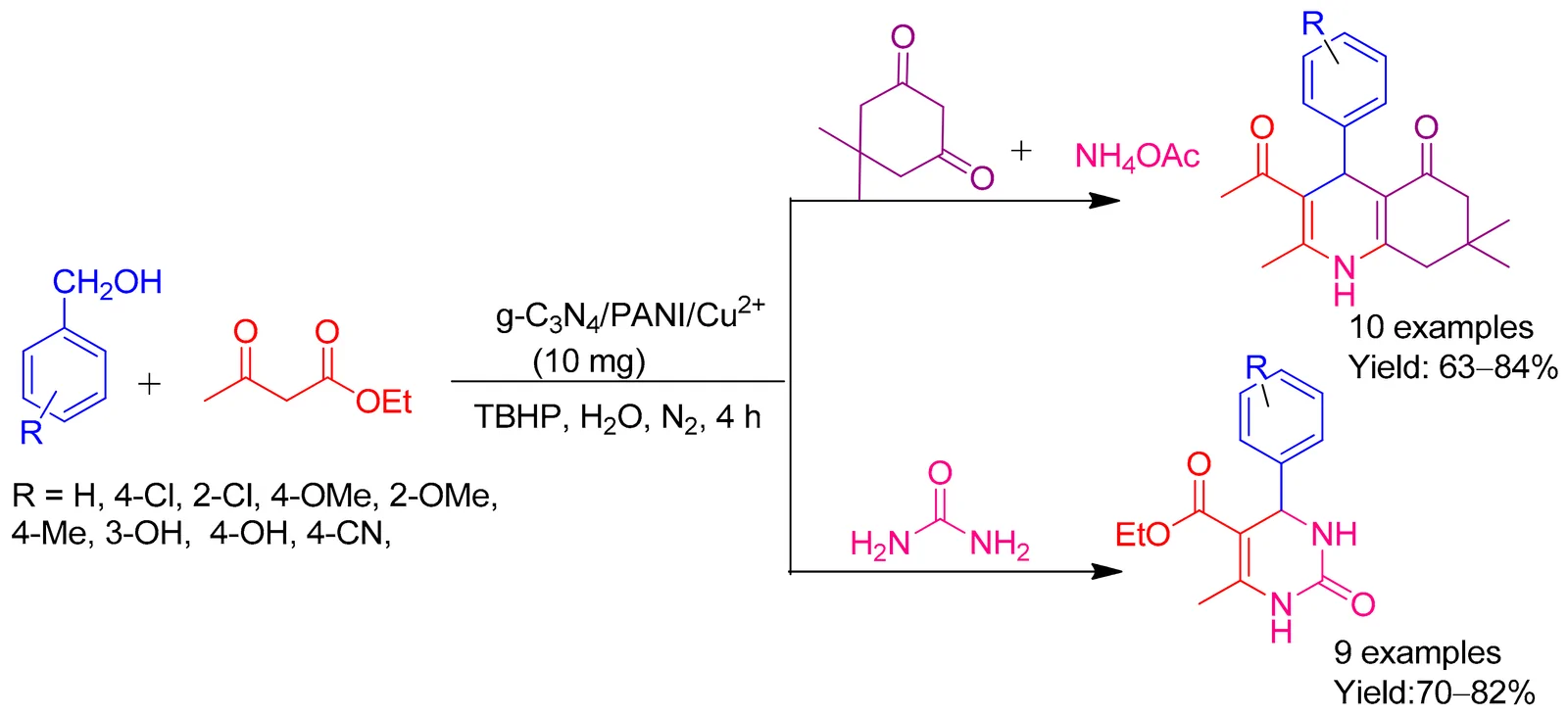
Synthesis of DHP and DHPM derivatives using a g-C_3_N_4_/PANI/Cu^2+^ catalyst.

In 2026, Moradi [44] and co-workers reported a g-C_3_N_4_/PANI/Cu^2+^ nanocomposite catalyst composed of graphitic carbon nitride (g-C_3_N_4_), Cu^2+^ ions and polyaniline (PANI). The resulting nanocomposite served as an efficient heterogeneous catalyst for the one-pot synthesis of polyhydroquinolines synthesized from benzyl alcohol, dimedone, ethyl acetoacetate, and NH_4_OAc, while dihydropyrimidines were obtained from benzyl alcohol, urea and ethyl acetoacetate in the presence of TBHP and water. The required products were obtained in 63–84% yields, and the catalyst maintained its activity after five consecutive reaction cycles. The synthetic methodology is illustrated in Scheme 9.

### 4.2. Synthesis of Quinoline Derivatives

The quinoline derivatives are a significant group of nitrogen-based fused heterocyclic compounds that have numerous claims in the fields of medicinal chemistry, pharmaceutical sciences, and materials chemistry. Quinoline rings are present in many biological molecules that possess various physiological effects, which include antimalarial, antibacterial, antiviral, anticancer, anti-inflammatory, and antifungal properties [45,46,47].

**Scheme 10:**
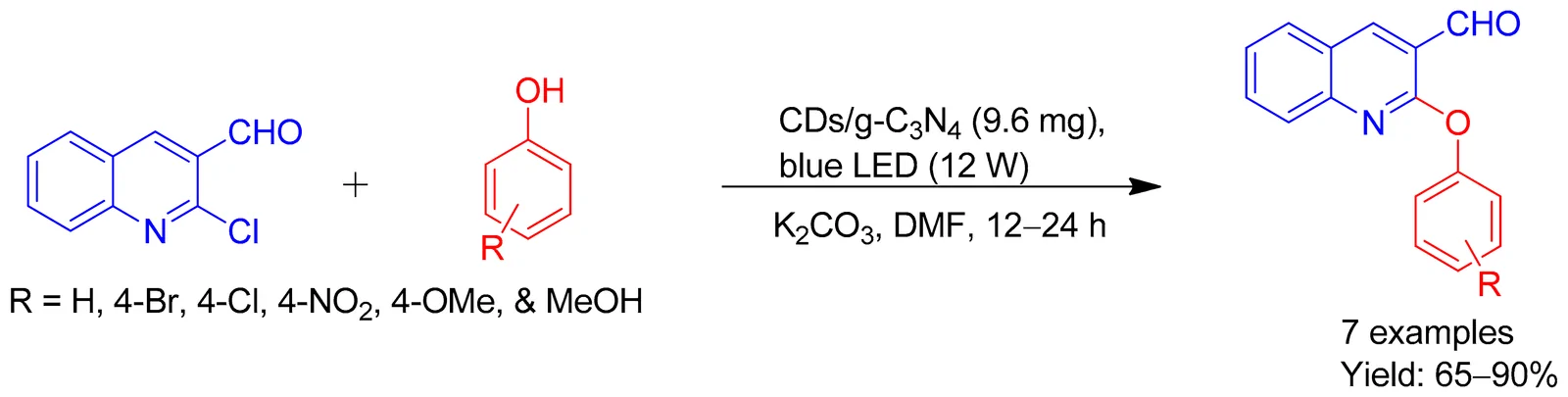
Synthesis of O-arylated-quinoline-carbaldehyde derivatives by using CDs/g-C_3_N_4_ catalyst.

In 2023, Manjupriya et al. [48] reported a facile CDs/g-C_3_N_4_ heterojunction consisting of carbon dots (CDs) loaded onto graphitic carbon nitride (g-C_3_N_4_) and evaluated its photocatalytic performance for the preparation of *O*-arylated quinoline-carbaldehyde derivatives. The reaction of 2-chloroquinoline-3-carbaldehyde with substituted phenols in the presence of K_2_CO_3_ in DMF under blue LED irradiation offered the required compounds in good to excellent yields (65–90%) within 12–24 h, as illustrated in Scheme 10. Furthermore, the photocatalyst exhibited good stability and was successfully recycled for up to five consecutive reaction cycles with minimal loss of activity.

**Scheme 11:**
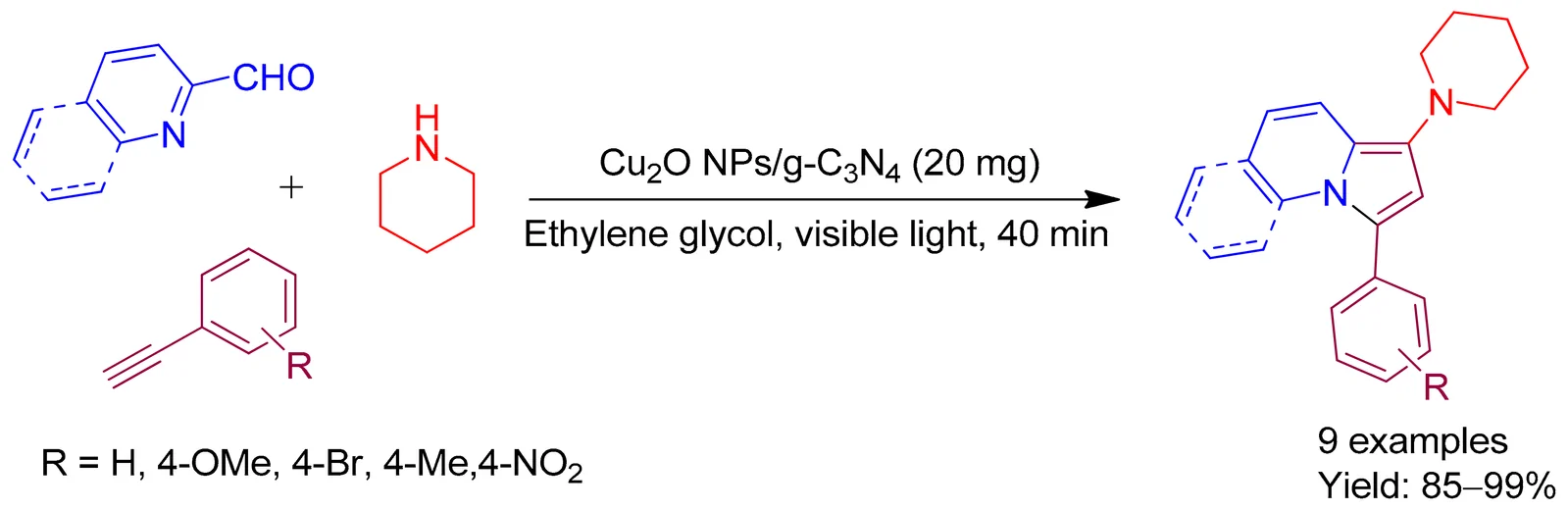
Synthesis of indolizines and pyrrolo-quinoline derivatives using Cu_2_ONPs/g-C_3_N_4_ catalyst.

In 2020, Anuj S. Sharma et al. [49] reported a photoactive Cu_2_O NPs@g-C_3_N_4_ nanocomposite, in which copper(I) oxide nanoparticles were supported on graphitic carbon nitride. The catalyst was competently used for the visible-light-mediated preparation of pyrroloquinoline and indolizine derivatives from quinoline-2-carboxaldehyde or picolinaldehyde, phenylacetylene, and piperidine in ethylene glycol. The reactions were completed within 40 min, giving the desired compounds in good to excellent yields (85–99%), as illustrated in Scheme 11. Furthermore, the photocatalyst was successfully recycled for up to five consecutive reaction cycles with only a marginal decrease in catalytic activity.

**Scheme 12:**
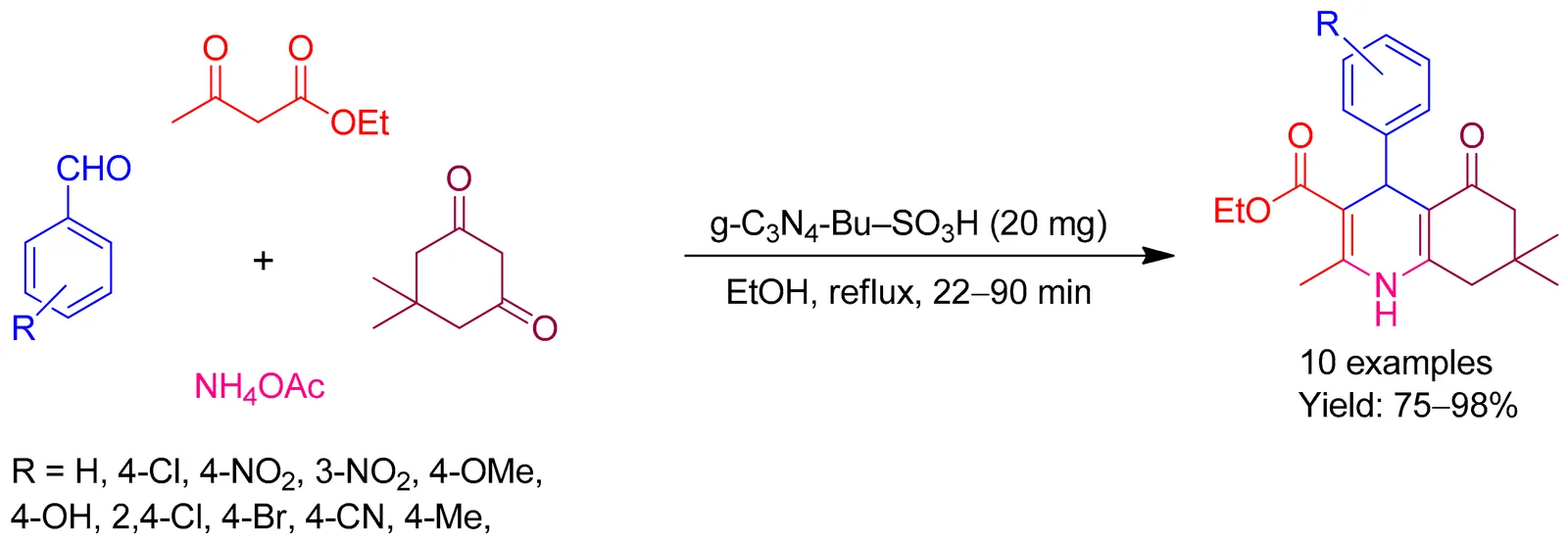
Synthesis of PHQ derivatives using CNNs-Bu–SO_3_H catalyst.

In 2022, Monavar Rahmati [50] and co-workers reported a covalently linked sulfonic acid (SO_3_H)-functionalized GCN nanosheet (SO_3_H@g-C_3_N_4_) catalyst. The catalyst efficiently promoted the preparation of PHQs via 4 CR of various benzaldehydes, dimedone, ammonium acetate, and ethyl acetoacetate in ethanol, affording the desired products in 22–90 min with 75–98% yields, as shown in Scheme 12. The catalyst demonstrated exceptional recyclability, preserving its catalytic efficacy over seven successive runs with negligible loss of action.

**Scheme 13:**
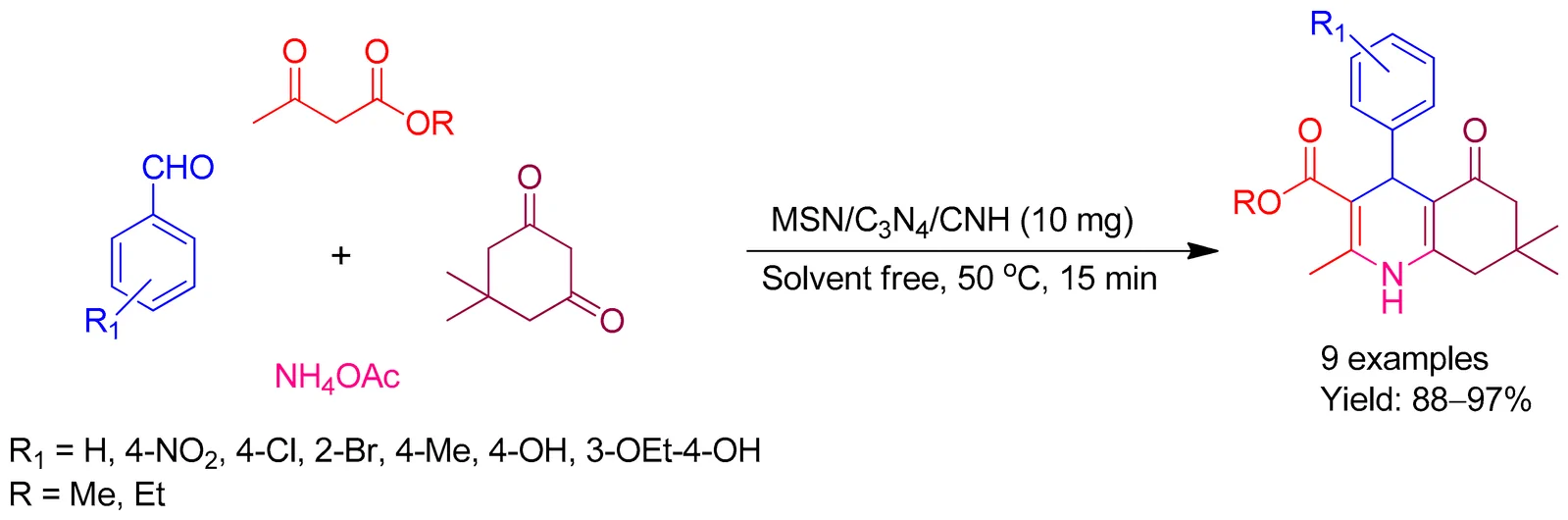
Synthesis of PHQ derivatives using the MSN/C_3_N_4_/CNH catalyst.

In 2023, Rahmati et al. [51] reported a copper nitrate hydroxide (CNH)-comprising mesoporous silica NP/graphitic carbon nitride (MSN/C_3_N_4_/CNH) composite as a robust catalyst. The catalyst efficiently promoted the synthesis of PHQ derivatives through the 4CR of dimedone, various benzaldehydes, NH_4_OAc and ethyl acetoacetate under solvent-free settings, affording the desired products within 15 min with 88–97% yields, as shown in Scheme 13. The catalyst showed exceptional recyclability, retaining its catalytic action over six repeated runs with only a minor decline in performance.

**Scheme 14:**
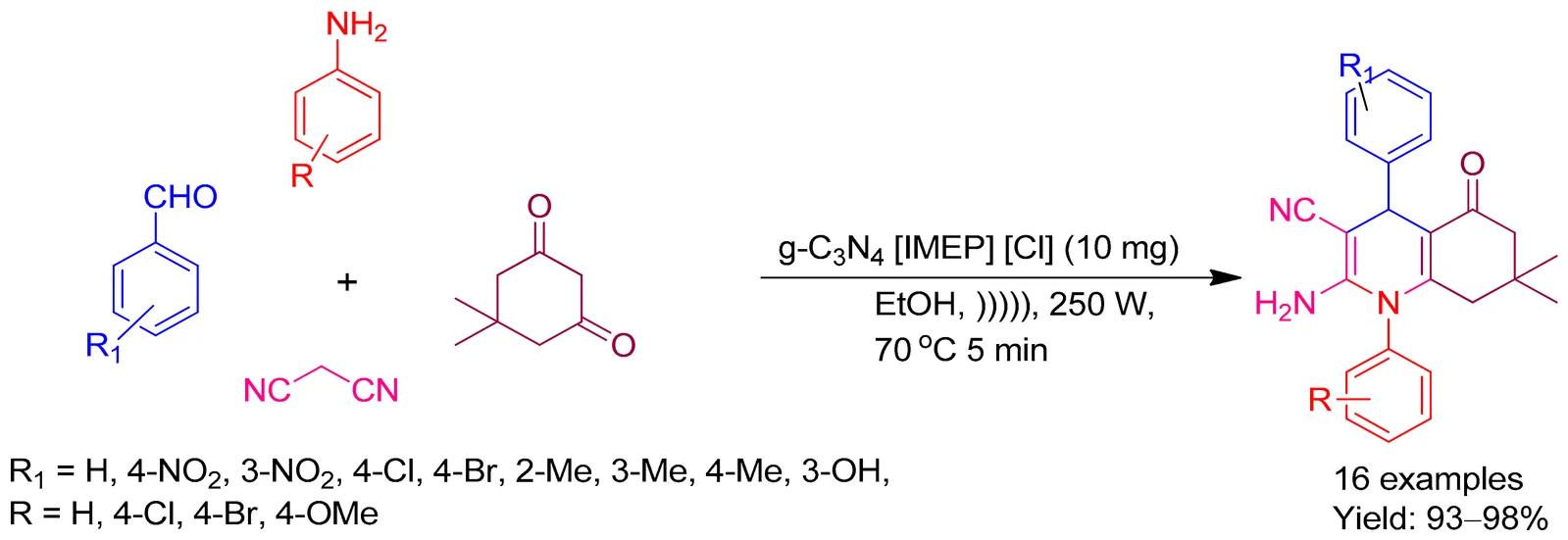
Synthesis of hexahydroquinoline frameworks using g-C_3_N_4_ [IMEP] [Cl] catalyst.

In 2024, Shafaati et al. [52] reported a mesoporous carbon nitride tube (CNT) catalyst fabricated using an ionic [IMEP][Cl]imidazole–epichlorohydrin copolymer as a template. The CNT catalyst efficiently promoted the regioselective synthesis of hexahydroquinoline derivatives through 4CR of malononitrile, dimedone, aniline and aromatic aldehydes under ultrasonication in water, giving the desired compounds within 5 min with 93–98% yields, as shown in Scheme 14. The catalyst demonstrated exceptional recyclability, maintaining its catalytic efficiency over seven repeated cycles with negligible loss of activity.

**Scheme 15:**
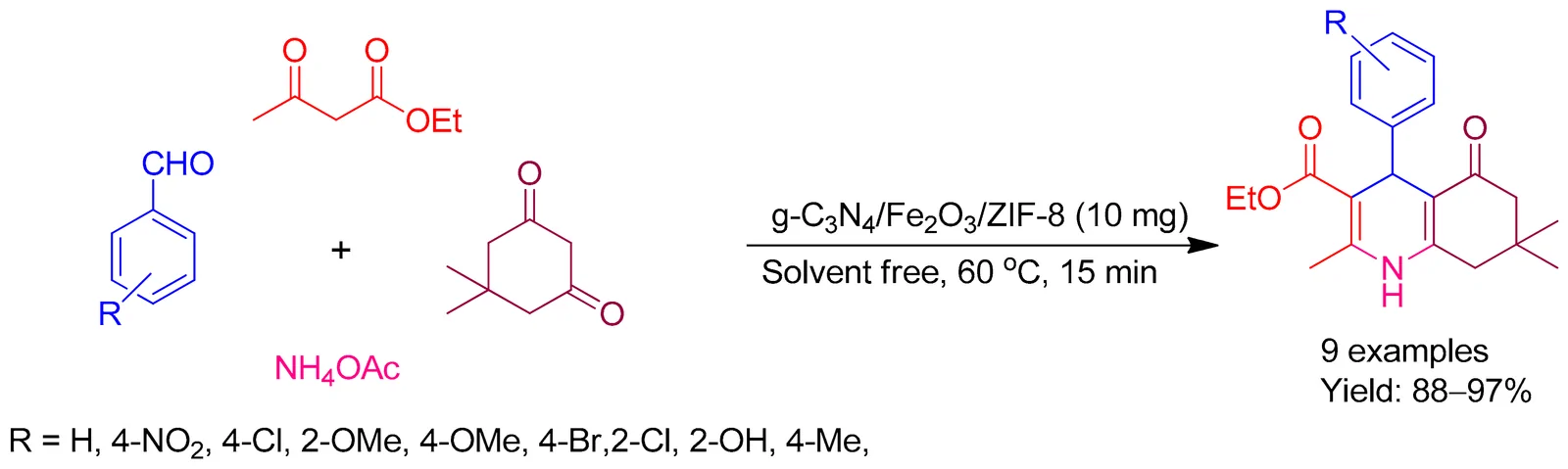
Synthesis of PHQ derivatives using the g-C_3_N_4_/Fe_2_O_3_/ZIF-8 catalyst.

In 2024, Ghiaci [53] and co-workers reported a g-C_3_N_4_/Fe_2_O_3_/ZIF-8 nanocomposite prepared by a straightforward synthetic approach. The nanocatalyst competently used for the preparation of PHQ derivatives through the 4CR of dimedone, benzaldehyde derivatives, ammonium acetate, and ethyl acetoacetate under solvent-free situations at 60 °C, affording the desired products within 15 min with 88–97% yields, as shown in Scheme 15. The catalyst exhibited excellent recyclability and was reused for five repeated runs without major loss of catalytic action.

**Scheme 16:**
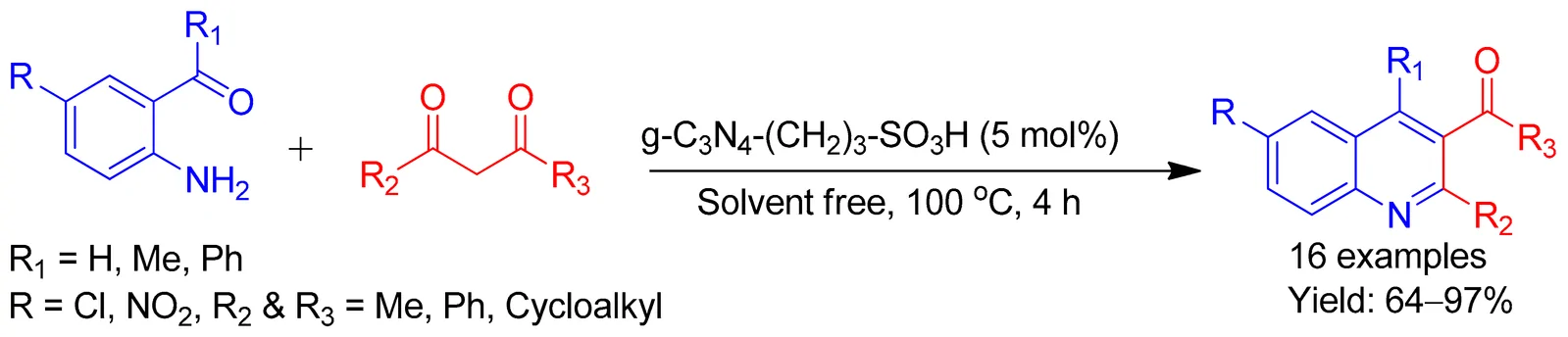
Synthesis of quinoline derivatives using g-C_3_N_4_-CO-(CH_2_)_3_-CO_2_H catalyst.

In 2024, Krishna [54] and co-workers reported a surface-functionalized GCN catalyst, g-C_3_N_4_-CO-(CH_2_)_3_-CO_2_H, synthesized from melamine via sequential functionalization with glutaric anhydride and 1,3-propanesultone. The catalyst effectively used the solvent-free protocol of quinoline derivatives through the condensation of α-methylene carbonyl compounds and 2-aminoaryl ketones, offering the desired products in 64–97% yields within 4 h. The heterogeneous catalyst showed superb recyclability, keeping its catalytic action over five repeated cycles with negligible loss in performance. The synthetic methodology is illustrated in Scheme 16.

### 4.3. Synthesis of Quinazoline Derivatives

Quinazoline derivatives are a valuable group of nitrogen-containing heterocyclic fusions, which have gained great interest in pharmacological and medicinal research due to their varied biological actions. The quinazoline moiety is well recognized as a privileged structure in drug discovery and is found in many biologically active compounds, especially those acting against cancer, bacteria, fungi, inflammation, viruses, and malaria. This structural diversity of quinazoline permits modification at various sites, leading to the design of bioactive and selective molecules [55,56,57].

**Scheme 17:**
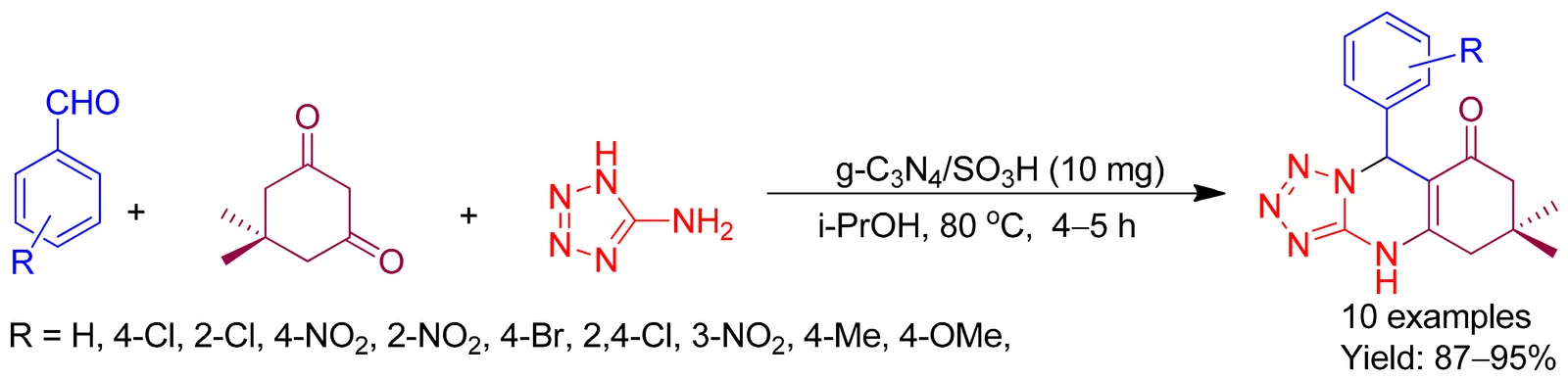
Synthesis of tetrazolo-quinazoline derivatives using g-C_3_N_4_/NHSO_3_H catalyst.

In 2020, Hassankhani et al. [58] reported a covalently bonded sulfamic acid-functionalized GCN (g-C_3_N_4_/NHSO_3_H) as a cost-effective catalyst. The catalyst efficiently promoted the preparation of quinazolines via 3CR of various benzaldehydes, 2-aminotetrazole, and dimedone in isopropanol at 80 °C, affording the desired products within 4–5 h with 87–95% yields, as shown in Scheme 17. The catalyst showed superb stability and was reused for at least eight sequential runs without any noticeable loss of catalytic activity.

**Scheme 18:**
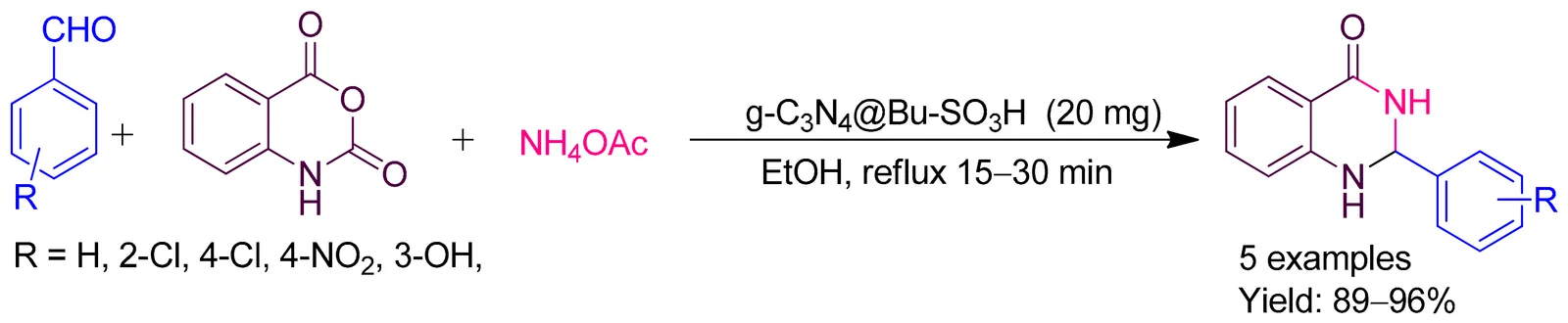
Synthesis of quinazoline derivatives using g-C_3_N_4_@Bu-SO_3_H catalyst.

In 2022, Ghafuri et al. [59] reported 1,4-butane sultone-functionalized GCN nanosheets (g-C_3_N_4_@Bu-SO_3_H) as an effective heterogeneous catalyst. The catalyst was employed for the preparation of quinazolines via the one-pot reaction of various benzaldehydes, isatoic anhydride, and ammonium acetate in ethanol under reflux, offering the desired products within 15–30 min with 89–96% yields, as shown in Scheme 18. The catalyst exhibited good catalytic efficiency and recyclability.

**Scheme 19:**
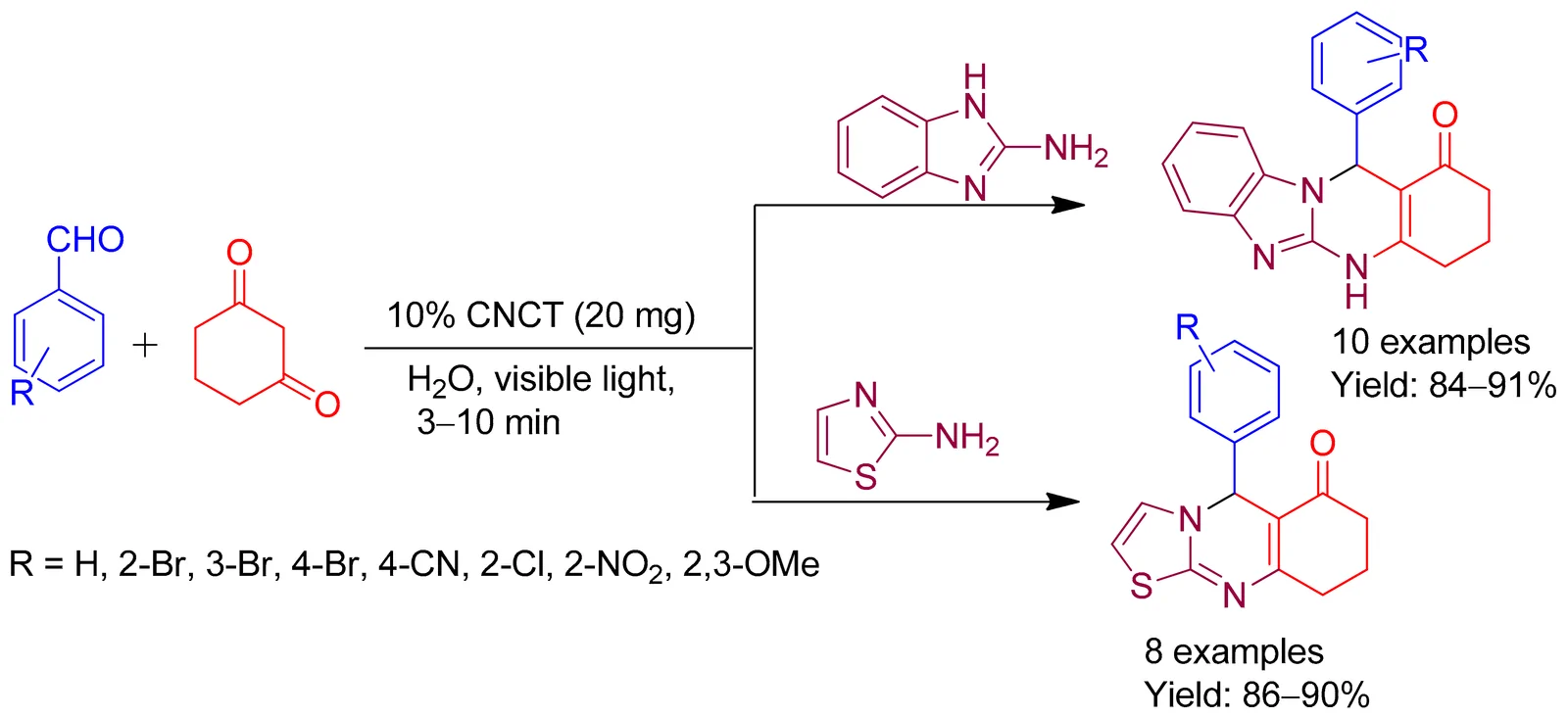
Synthesis of quinazolinone derivatives using CNCT under visible light irradiation.

In 2023, Arunachalapandi [60] and co-workers reported a Cu_3_TiO_4_ nanoparticle-supported exfoliated g-C_3_N_4_ nanosheet (CNCT) photocatalyst, where Cu_3_TiO_4_ nanoparticles were synthesized using *Citrus limon* juice extract as a capping agent. The nanocomposite efficiently catalysed the synthesis of quinazolinone derivatives via the multicomponent reaction of 2-aminothiazole or 2-aminobenzimidazole, 1,3-cyclohexanedione, and various aldehydes in water, offering the products in 3–10 min with 84–91% yields, as illustrated in Scheme 19. The photocatalyst was reused for five consecutive cycles without appreciable loss of photocatalytic activity.

**Scheme 20:**
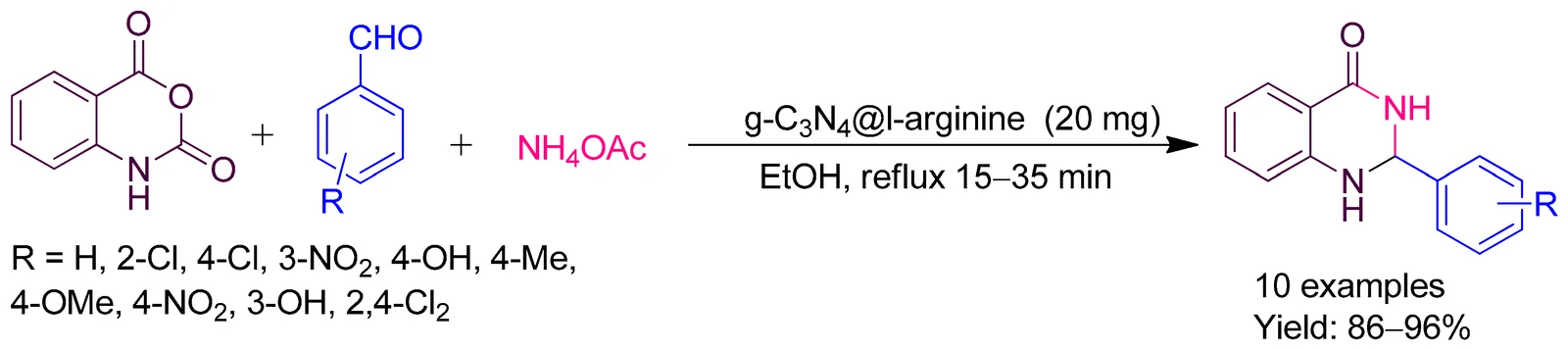
Synthesis of quinazoline derivatives using g-C_3_N_4_@L-arginine catalyst.

In 2021, Ghafuri et al. [61] reported a GCN-supported L-arginine (g-C_3_N_4_@L-arginine) nanocatalyst as an effective heterogeneous catalyst for the synthesis of quinazoline derivatives. The catalyst was used for the one-pot 3CR of ammonium acetate, benzaldehyde, and isatoic anhydride, in ethanol, offering the required products in good to excellent yields (86–96%) within 15–35 min, as illustrated in Scheme 20. Furthermore, the catalyst revealed good recyclability and was efficiently reused for up to five sequential reaction cycles without substantial loss of catalytic action.

### 4.4. Synthesis of Quinoxaline Derivatives

The quinoxaline derivatives belong to the category of nitrogen heterocyclic compounds, which have been successfully applied in medicinal chemistry, pharmaceutics, and materials science. Various biological actions such as anticancer, antimicrobial, antiviral, antitubercular, anti-inflammatory, and antioxidant effects have been established with the use of the quinoxaline moiety [62,63].

**Scheme 21:**
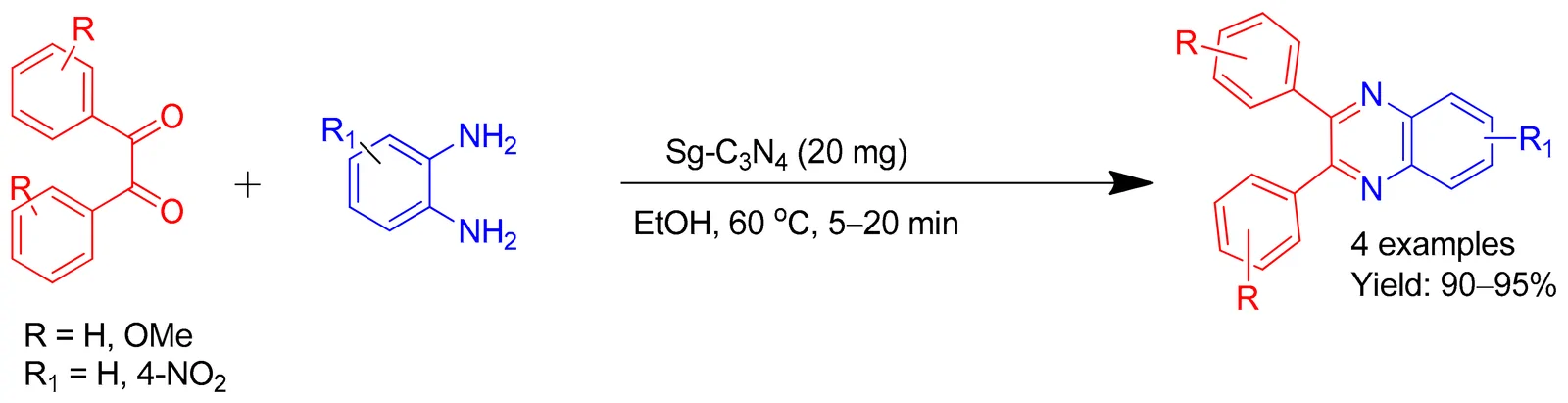
Synthesis of quinoxaline derivatives using Sg-C_3_N_4_ catalyst.

Ghafuri et al. (2019) [64] reported a sulfonated graphitic carbon nitride (Sg-C_3_N_4_) heterogeneous catalyst prepared through a simple and facile procedure using commercially available precursors. The catalyst showed remarkable catalytic efficiency in the preparation of quinoxaline derivatives via the reaction of various diamine with 1,2-diketones in ethanol. The transformation proceeded efficiently within 5–20 min, providing the desired compounds in 90–95% yields, as depicted in Scheme 21.

**Scheme 22:**
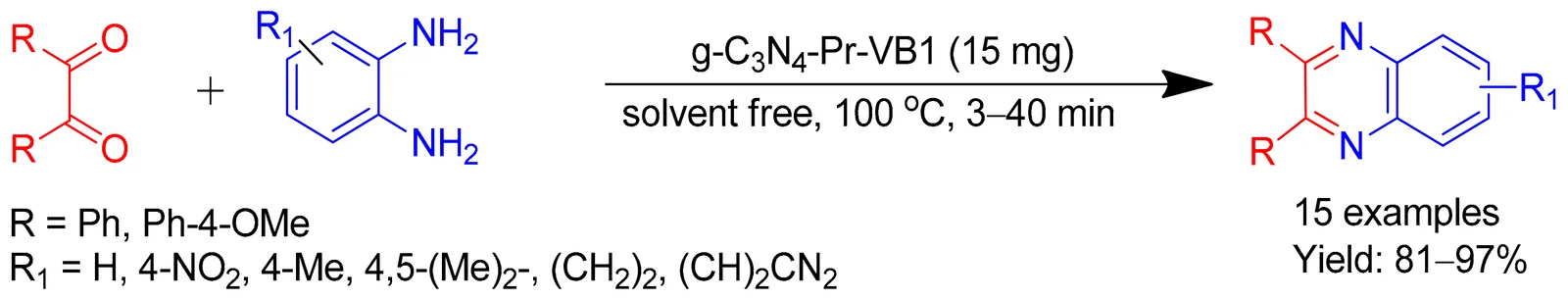
Synthesis of quinoxaline derivatives using CN-Pr-VB1 catalyst.

In 2019, Rashidizadeh [65] and co-workers reported the first covalent functionalization of g-C_3_N_4_ nanosheets with vitamin B1 using 1,3-dibromopropane linker to construct the CN-Pr-VB1 organocatalyst. The catalyst efficiently promoted the solvent-free preparation of quinoxalines via the condensation of *o*-phenylenediamine and benzil, affording the desired products in tremendous yields (81–97%) within 3 min, as illustrated in Scheme 22. Moreover, the catalyst was readily recovered and reused for up to six successive cycles without major loss of catalytic activity.

### 4.5. Synthesis of Imidazole Derivatives

Imidazole analogues are a group of heterocyclic compounds having significant importance in medicine and pharmacology due to their widespread uses in different scientific areas. Imidazole ring is a basic structural element in various biological molecules exhibiting a wide range of biological action such as antimicrobial, antioxidant, antiviral, antifungal, antitubercular and anti-inflammatory effects [66,67,68].

**Scheme 23:**
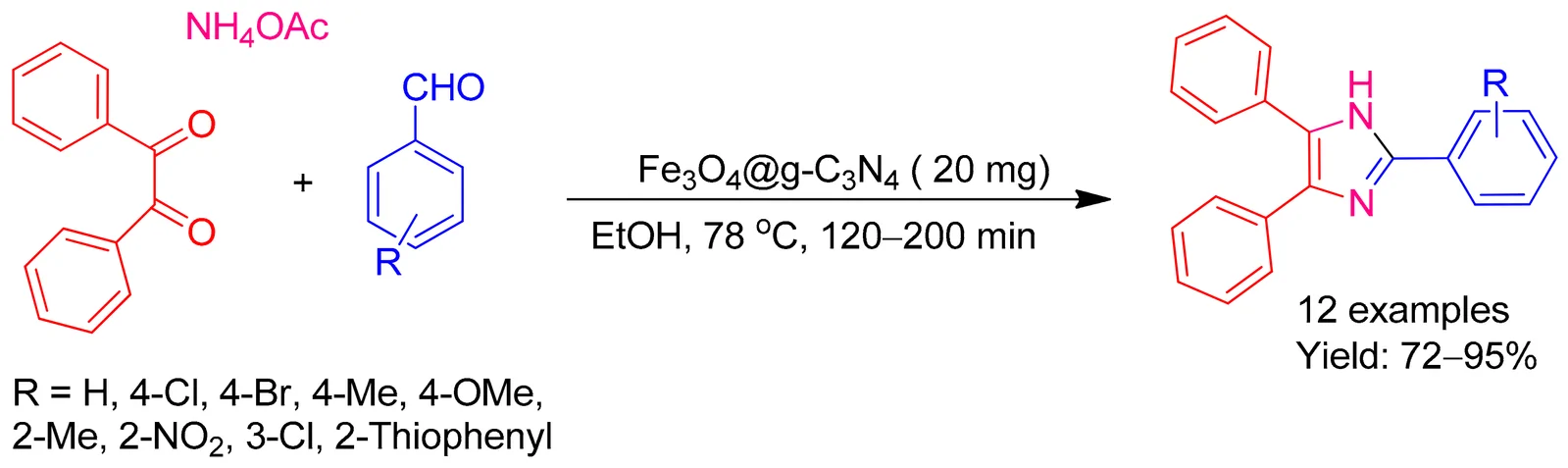
Synthesis of imidazole derivatives using Fe_3_O_4_@g-C_3_N_4_ catalyst.

In 2018, Ahooie et al. [69] reported a reusable Fe_3_O_4_@g-C_3_N_4_ magnetic nanocomposite as an effective heterogeneous catalyst for the one-pot 3CR of imidazole derivatives. The reaction of aldehydes, ammonium acetate, and 1,2-diketones in ethanol at 78 °C offered the required products in excellent yields (72–95%) within 120–200 min, as shown in Scheme 23. Furthermore, the magnetic Fe_3_O_4_@g-C_3_N_4_ catalyst was recovered and successfully reused for up to six repeated reaction cycles without substantial loss of catalytic action.

**Scheme 24:**
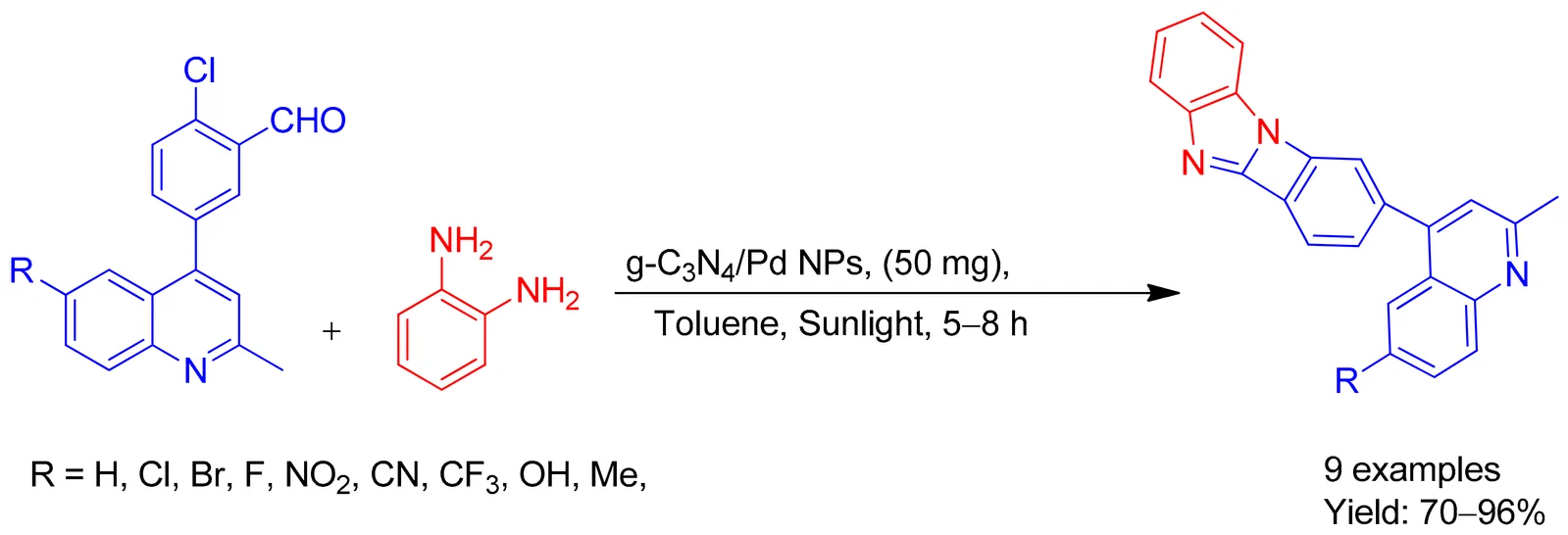
Synthesis of quinoline-fused benzoazeto-benzimidazole using Pd/g-C_3_N_4_ catalyst.

In 2026, Kouhdareh et al. [70] developed an ultrathin Pd/g-C_3_N_4_ photocatalyst by anchoring ultra-small, monodisperse Pd NPs onto crystalline 2D graphitic carbon nitride nanosheets. The catalyst showed exceptional visible-light-driven catalytic activity for the preparation of benzoazeto-benzimidazole derivatives via the intramolecular cyclization of *o*-phenylenediamine with 2-chloro-5-(2-methylquinoline-4-yl) benzaldehydes in the presence of Cs_2_CO_3_ using toluene as the solvent. The required products were acquired in good to excellent yields (70–96%) within 5–8 h, as shown in Scheme 24. Sunlight offers a renewable and energy-efficient alternative to artificial Xe or LED lamps, while visible-light-responsive g-C_3_N_4_ promotes photogenerated charge-carrier formation and dispersed Pd nanoparticles facilitate interfacial electron transfer.

**Scheme 25:**
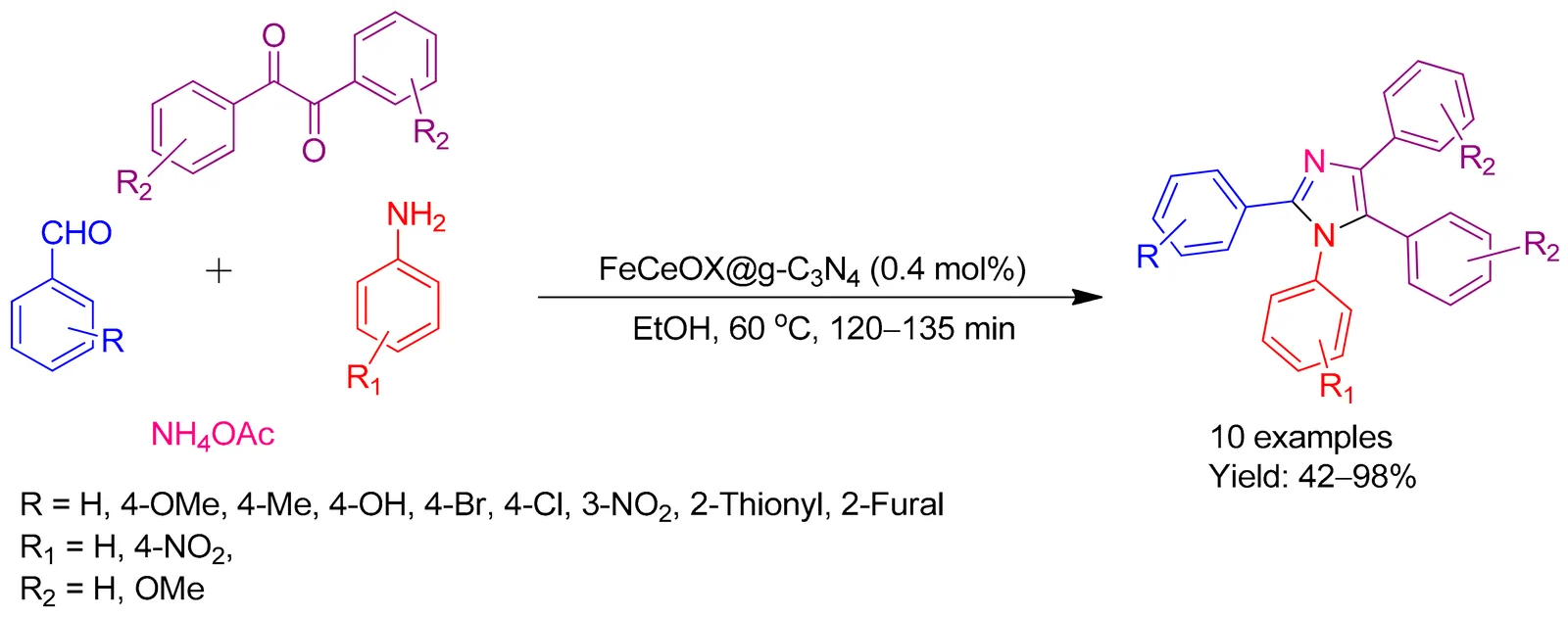
Synthesis of tetraphenyl imidazole using FeCeO_x_@g-C_3_N_4_ catalyst.

In 2023, Azizi et al. [71] developed a novel FeCeO_x_@g-C_3_N_4_ nanocomposite through a combination of sonication and hydrothermal methods. The resulting nanocatalyst efficiently promoted the one-pot preparation of tetrasubstituted imidazoles from benzil, benzaldehyde, ammonium acetate (NH_4_OAc), and aniline in ethanol at 60 °C. The required compounds were attained in modest to excellent yields (42–98%) within 120–135 min, as illustrated in Scheme 25. Furthermore, the FeCeO_x_@g-C_3_N_4_ catalyst exhibited excellent reusability and retained its catalytic activity after five consecutive reaction cycles.

**Scheme 26:**
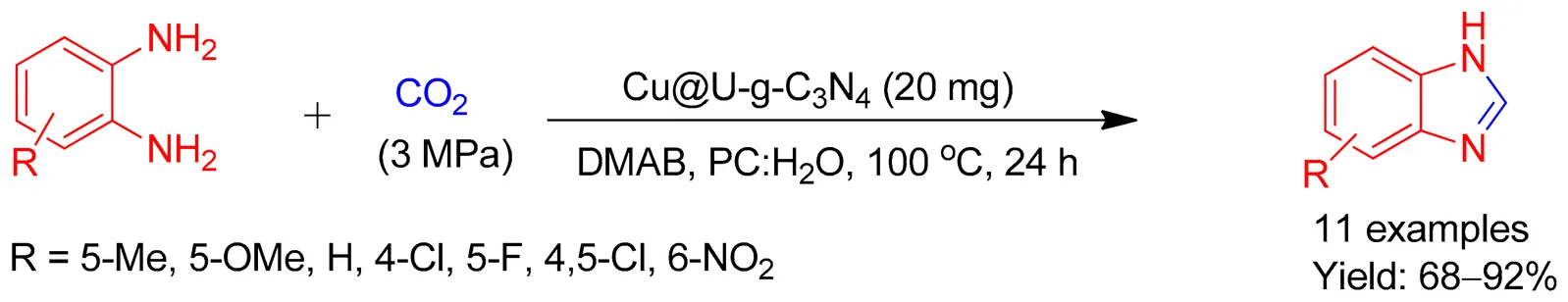
Synthesis of benzimidazole derivatives using Cu@U-g-C_3_N_4_ catalyst.

In 2019, Phatake [72] and co-workers reported a Cu@U-g-C_3_N_4_ catalyst prepared by immobilizing CuCl_2_ on urea-derived porous graphitic carbon nitride (Cu@U-g-C_3_N_4_). The catalyst was employed in the cyclization of *o*-phenylenediamines with CO_2_ using DMAB (dimethylamine borane) as the reducing agent in propylene carbonate/water at 100 °C, affording benzimidazole derivatives in 24 h with 68–92% yields, as illustrated in Scheme 26. The catalyst was retrieved and reused for at least four consecutive cycles without any noticeable loss of catalytic activity.

**Scheme 27:**
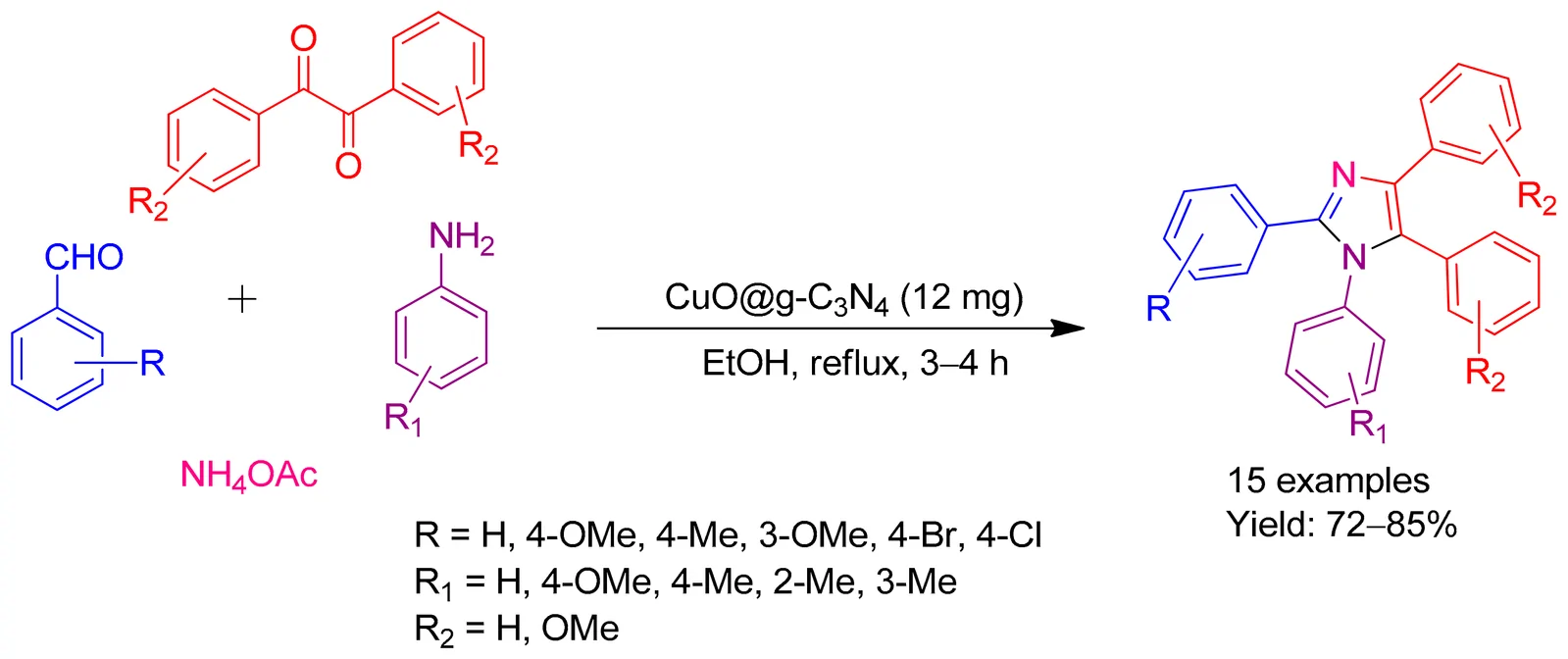
Synthesis of imidazole using CuO@g-C_3_N_4_.

In 2023, Gajurel [73] and co-workers reported CuO@g-C_3_N_4_ as an efficient heterogeneous catalyst for the preparation of multisubstituted imidazole derivatives. The reaction of benzil, aniline, benzaldehyde, and ammonium acetate under ultrasonic-assisted reflux afforded the required products in 72–85% yields within 2–4 h. The catalyst demonstrated exceptional recyclability, retaining its catalytic action over five repeated reaction cycles with negligible loss in performance. The synthetic methodology is illustrated in Scheme 27.

**Scheme 28:**
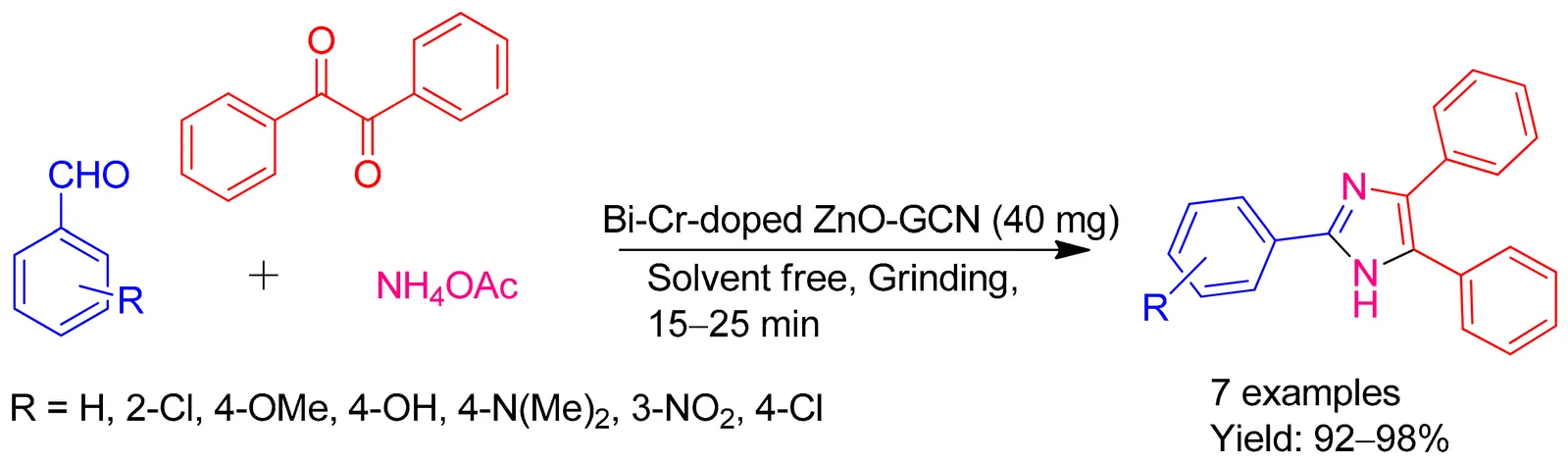
Synthesis of imidazole derivatives using Bi/Cr–ZnO@g-C_3_N_4_ catalyst.

In 2024, Patel [74] and co-workers reported a Bi (4%)- and Cr (1%)-doped ZnO/graphitic carbon nitride (Bi/Cr–ZnO@g-C_3_N_4_) nanocomposite as a competent catalyst for the solvent-free preparation of imidazoles. The catalyst promoted the one-pot reaction of aromatic aldehydes, benzoin/benzil and ammonium acetate (NH_4_OAc) under mechanochemical grinding conditions, affording the desired products in 92–98% yields within 15–25 min. The synthetic methodology is illustrated in Scheme 28.

### 4.6. Synthesis of Triazole Derivatives

Triazole derivatives represent an important class of heterocycles containing high nitrogen content with wide-ranging uses in medicinal chemistry, pharmaceuticals, agrochemicals, and materials science. Triazole ring has excellent chemical stability and good hydrogen bonding and coordination ability, which makes it an important pharmacophore in designing bioactive molecules. Triazoles have various biological actions such as antimicrobial, antifungal, antiviral, anti-inflammatory, anticancer and antitubercular actions [75,76,77].

**Scheme 29:**
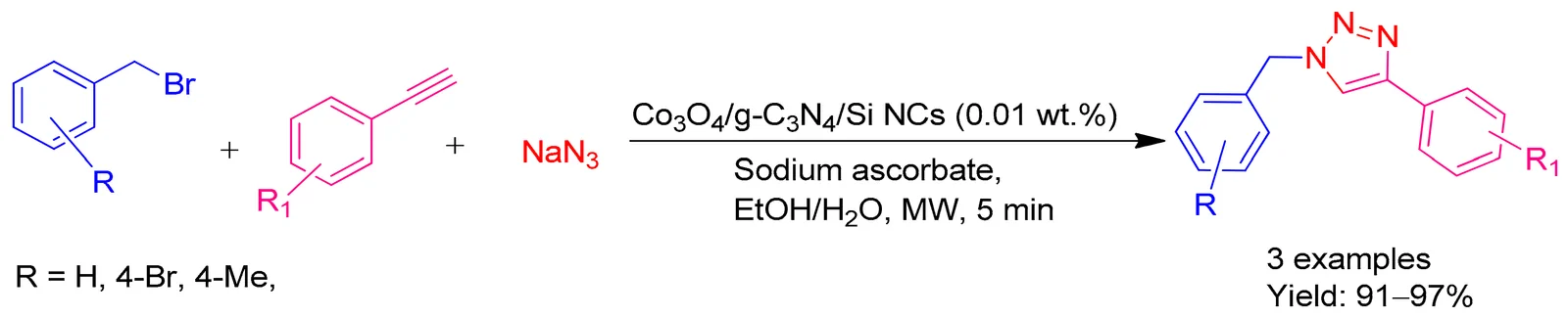
Synthesis of triazole derivatives using Co_3_O_4_/g-C_3_N_4_Si catalyst.

In 2025, Qahtani [78] and associates reported a Co_3_O_4_/g-C_3_N_4_Si nanocomposite as a competent catalyst for the microwave-assisted click protocol of triazole derivatives. The reaction of sodium azide, benzyl bromides, phenylacetylene, and sodium ascorbate in aqueous ethanol afforded the required products in 91–97% yields within 5 min. The catalyst revealed exceptional recyclability and was successfully reused for up to five repeated reaction cycles with no substantial loss of catalytic action. The synthetic methodology is illustrated in Scheme 29.

**Scheme 30:**
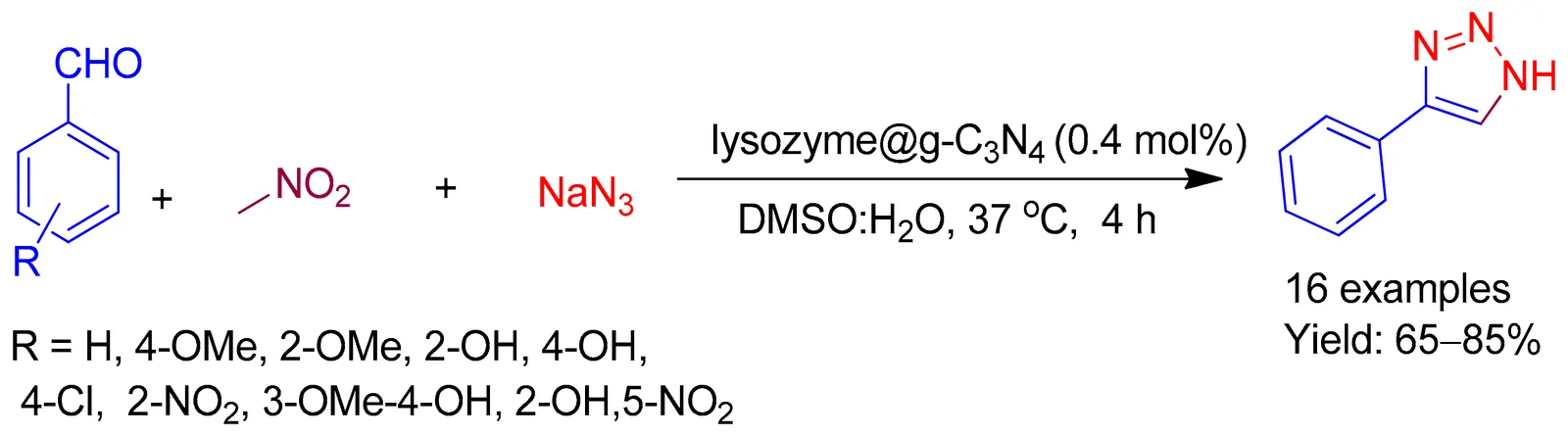
Synthesis of triazole derivatives using the lysozyme@g-C_3_N_4_ biocatalyst.

In 2025, Piraman [79] and co-workers reported a metal-free biocatalytic system based on lysozyme immobilized on g-C_3_N_4_. The immobilized biocatalyst efficiently promoted the synthesis of triazoles from nitromethane, aromatic aldehydes, and sodium azide in a DMSO/H_2_O medium, affording the desired products within 4 h with 65–85% yields, as shown in Scheme 30. The catalyst revealed good recyclability and was reused for up to three succeeding runs with retained catalytic action.

**Scheme 31:**
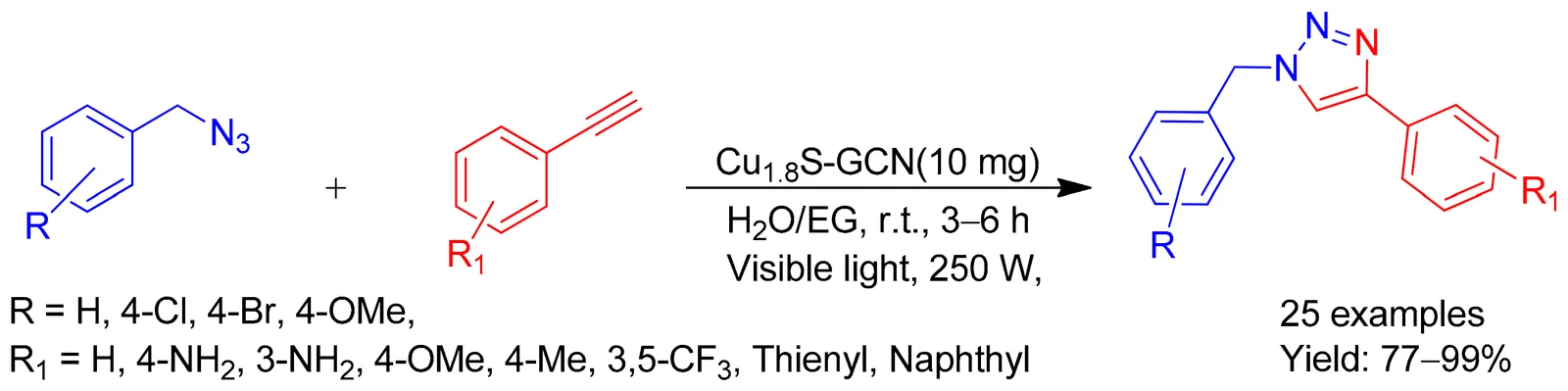
Synthesis of triazoles using Cu_1_._8_S–g-C_3_N_4_ catalyst.

In 2023, Gogoi et al. [80] reported a copper sulfide–GCN (Cu_1.8_S–g-C_3_N_4_) nanocomposite as an efficient visible-light photocatalyst. The catalyst efficiently stimulated the preparation of triazoles via the cycloaddition of benzyl azide and phenylacetylene under visible-light irradiation, affording the desired products within 3–6 h with 77–99% yields, as illustrated in Scheme 31. The photocatalyst showed exceptional stability and was reused for eight repeated cycles without substantial loss of catalytic activity.

**Scheme 32:**
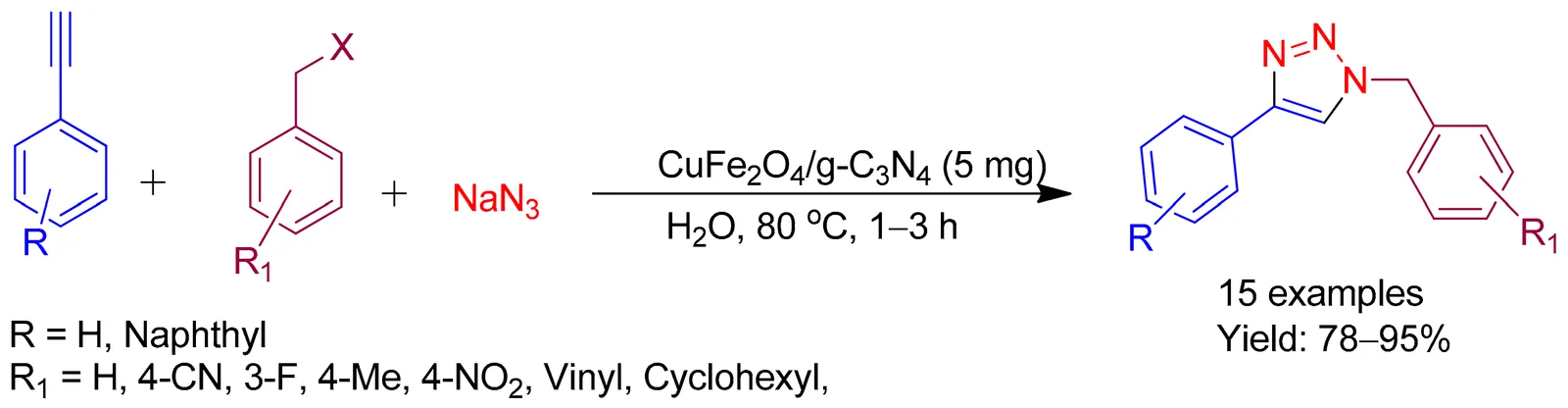
Synthesis of triazole derivatives using CuFe_2_O_4_/g-C_3_N_4_ catalyst.

In 2019, Dariush Khalili et al. [81] reported magnetic CuFe_2_O_4_/g-C_3_N_4_ hybrids synthesized via a facile one-step method. The nanocomposite efficiently catalysed the synthesis of triazole derivatives through the in situ generation of organic azides from alkyl halides or epoxides followed by cycloaddition with alkynes in water at 80 °C, giving the chosen products within 1–3 h with 78–95% yields, as shown in Scheme 32. The catalyst showed superb recyclability and was reused for six sequential cycles without substantial loss of catalytic activity.

**Scheme 33:**
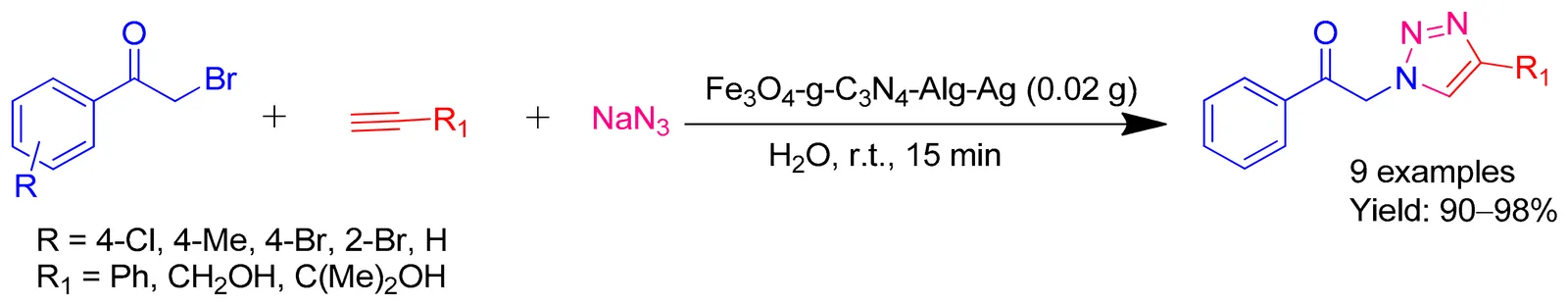
Synthesis of triazole derivatives using the Fe_3_O_4_-g-C_3_N_4_-alginate-Ag catalyst.

In 2021, Daraie [82] and co-workers reported the Ag nanoparticle-immobilized Fe_3_O_4_/g-C_3_N_4_/alginate nanocomposite as an effectual and recyclable catalyst for the regioselective preparation of triazoles via a click reaction. The catalyst utilized the one-pot reaction of sodium azide, alkyl halides/α-haloketones, and terminal alkynes in aqueous medium, offering the required products in excellent yields (90–98%) within 15 min, as illustrated in Scheme 33. Moreover, the Fe_3_O_4_-g-C_3_N_4_-alginate-Ag catalyst was successfully reused for up to seven consecutive cycles with no major loss in catalytic action.

### 4.7. Synthesis of Tetrazole Derivatives

Tetrazoles constitute one of the many significant classes of nitrogen-containing heterocyclics that have drawn considerable attention due to their relevance in medicinal chemistry, pharmaceuticals, and materials science. The tetrazole ring system has numerous biological properties, including being antihypertensive, antimicrobial, anticancer, anti-inflammatory, antiviral, and antitubercular [83,84].

**Scheme 34:**
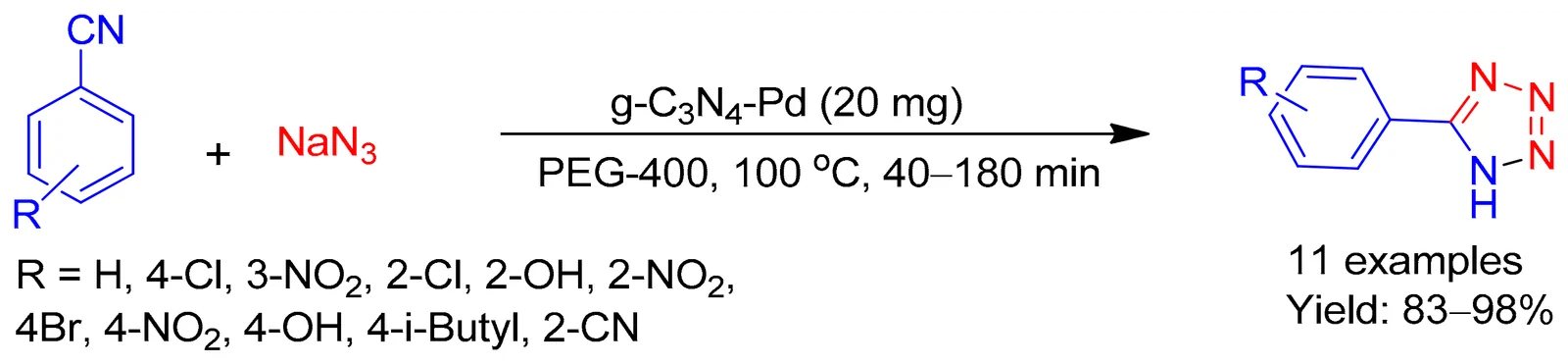
Synthesis of tetrazoles using (Pd@g-C_3_N_4_) catalyst.

In 2026, Nafis Ahmad et al. [85] reported a palladium-anchored organically modified GCN (Pd@g-C_3_N_4_) nanocatalyst as an effective and durable catalyst for the preparation of tetrazole derivatives. The catalyst promoted the reaction of various benzonitriles with sodium azide in PEG-400, affording the corresponding tetrazoles in 40–180 min with 83–98% yields, as illustrated in Scheme 34. The nanocatalyst also exhibited superb recyclability and was reused for five repeated cycles without substantial loss of catalytic action.

### 4.8. Synthesis of Chromene Derivatives

The chromenes belong to a significant family of oxygen-containing heterocycles which have established much consideration in medicinal chemistry, pharmaceutics, and the synthesis of natural products owing to their wide variety of biological performances. The chromene structure is incorporated in many naturally existing chemical substances and bioactive substances, and it shows wide-spectrum activity as an anticancer, antimicrobial, anti-inflammatory, antiviral, antioxidant, and antidiabetic agent [86,87,88].

**Scheme 35:**
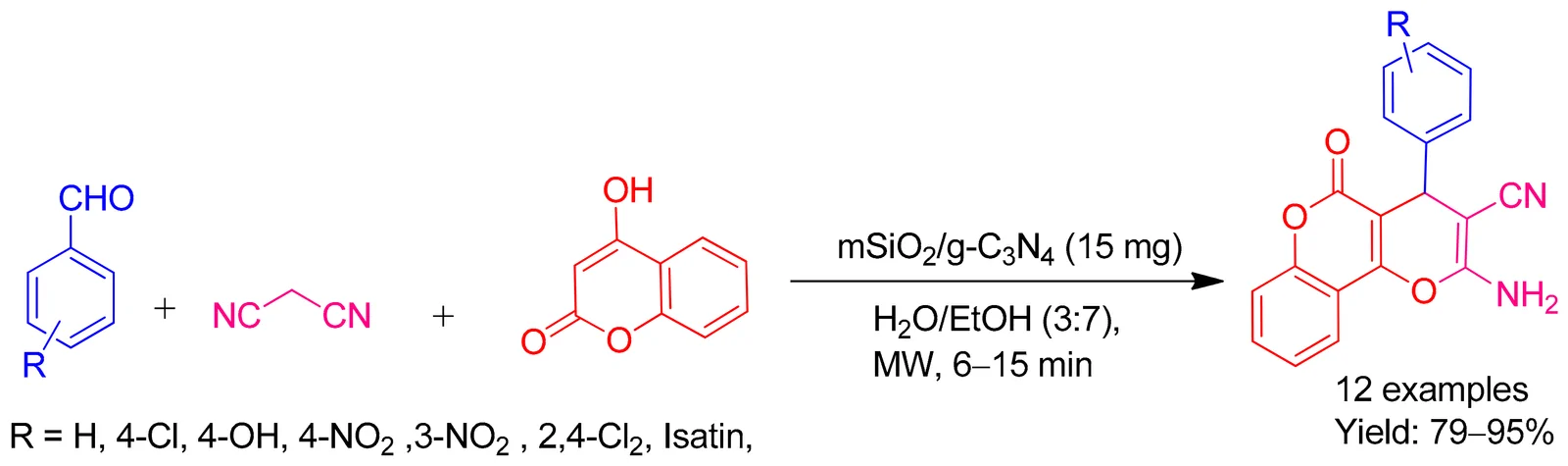
Synthesis of pyranochromenes using mSiO_2_/g-C_3_N_4_ catalyst.

In 2022, Neamani [89] and co-workers developed magnetic mesoporous silica/GCN composites (mSiO_2_/g-C_3_N_4_) by decorating mesoporous silica nanoparticles with g-C_3_N_4_ layers through the calcination of immobilized melamine. The catalytic execution was assessed in the microwave-assisted preparation of chromenes from 4-hydroxycoumarin, malononitrile, and benzaldehyde in an ethanol/water medium. The wanted products were attained in good yields (79–95%) within 6–15 min, as illustrated in Scheme 35. Furthermore, the magnetic catalyst was readily recovered and reused for up to four repeated cycles with minimal loss of catalytic activity.

**Scheme 36:**
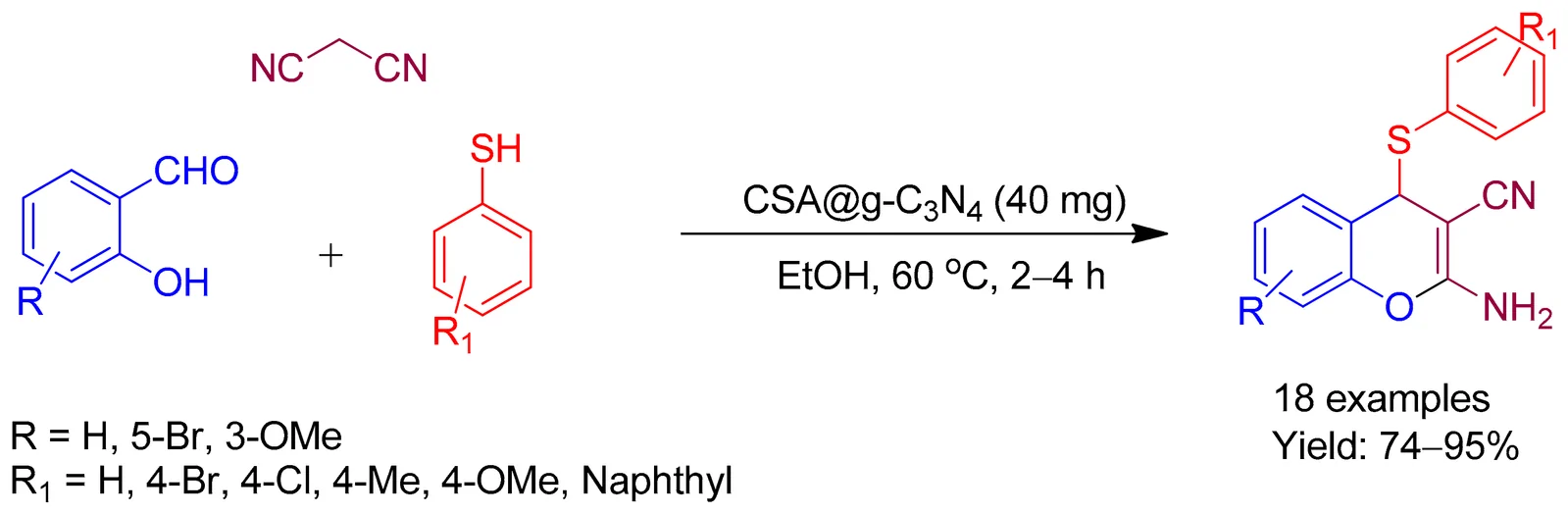
Synthesis of substituted chromene derivatives catalysed by CSA@g-C_3_N_4_ catalyst.

In 2023, Hosseini et al. [90] developed a CSA@g-C_3_N_4_ heterogeneous acidic catalyst by immobilizing camphorsulfonic acid (CSA) onto the surface of g-C_3_N_4_ under mild conditions. The catalyst effectively used the one-pot condensation of thiophenol, salicylaldehyde, and malononitrile in ethanol at 60 °C, affording substituted chromenes in good to excellent yields (74–95%) within 2–4 h, as illustrated in Scheme 36. Moreover, the CSA@g-C_3_N_4_ catalyst exhibited excellent stability and was successfully recovered and reused for up to ten consecutive reaction cycles without major loss of catalytic action.

**Scheme 37:**
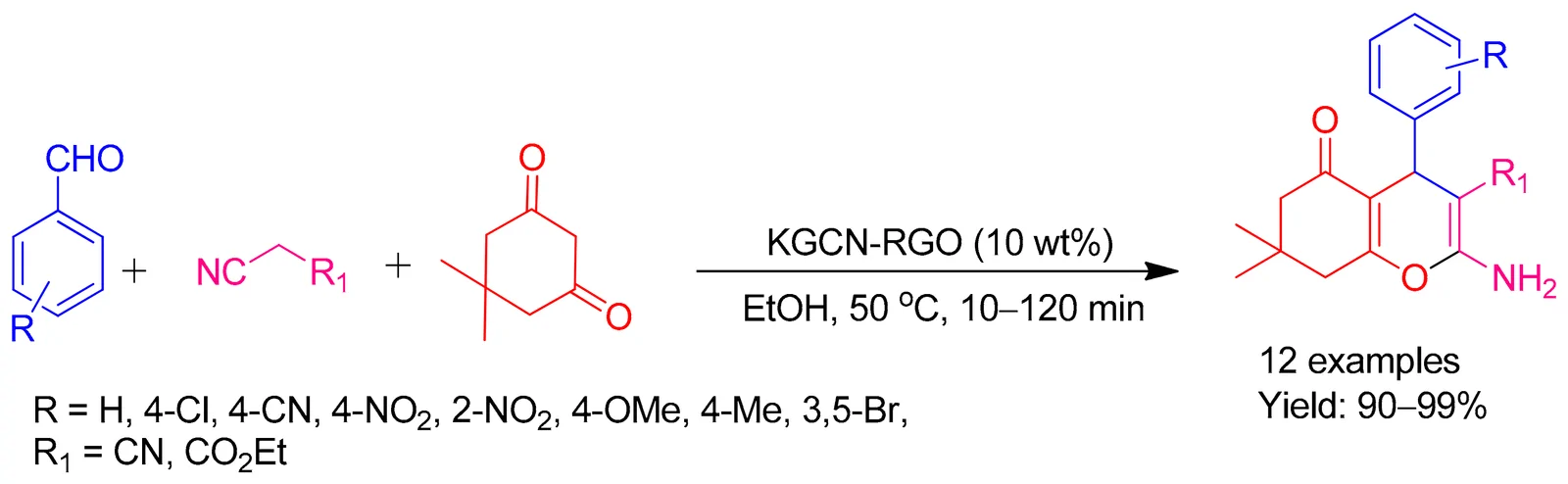
Synthesis of substituted chromenes using KGCN−RGO catalyst.

In 2018, Bahuguna [91] and co-workers reported a potassium-functionalized g-C_3_N_4_ /reduced graphene oxide (KGCN/RGO) nanocomposite prepared via a facile hydrothermal method. The catalyst competently used the preparation of aryl-substituted chromenes through 3CR of dimedone, active methylene compounds, and various benzaldehydes in ethanol at 50 °C, affording the products in 10–120 min with 90–99% yields, as illustrated in Scheme 37. The catalyst was reused for five repeated cycles without substantial loss of catalytic activity.

**Scheme 38:**
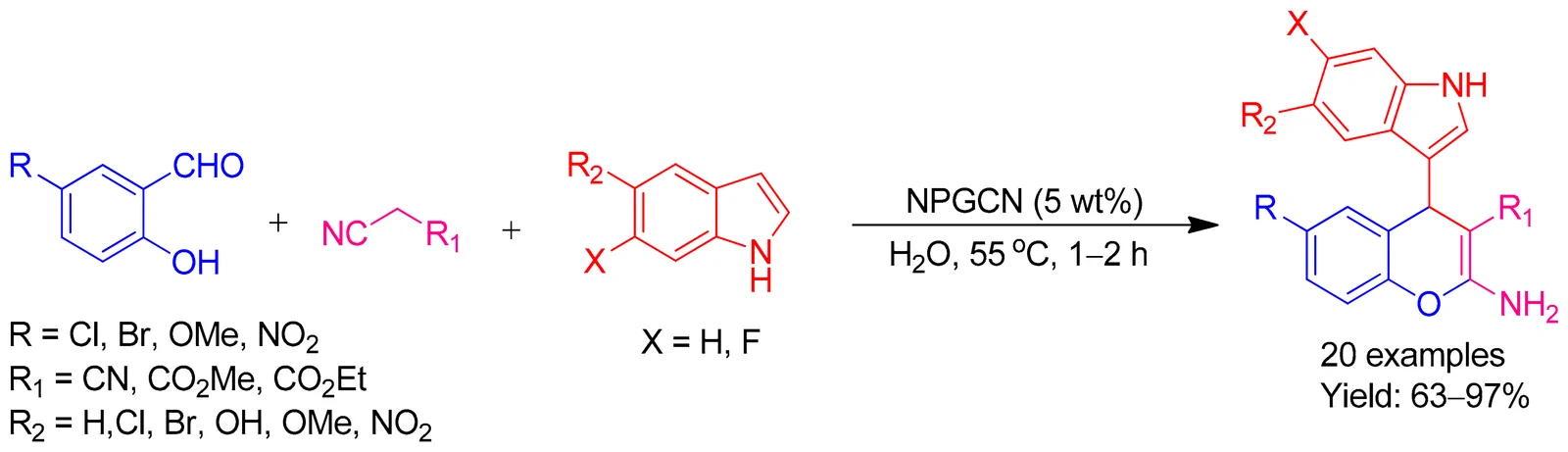
Synthesis of indole-substituted chromene derivatives using the NPGCN catalyst.

In 2018, Bahuguna et al. [92] reported a polyaniline/g-C_3_N_4_ (PANI/g-C_3_N_4_) nanocomposite prepared by the oxidative polymerization of aniline on ammonia-doped g-C_3_N_4_ nanosheets, yielding the PANI/g-C_3_N_4_ nanocomposite (NPGCN). The catalyst competently used the preparation of indole-substituted chromenes through the multicomponent reaction of salicylaldehydes, indole, and malononitrile in water at 50 °C, affording the desired products within 1–2 h with 63–97% yields, as shown in Scheme 38. The catalyst exhibited excellent recyclability and was reused for five successive cycles with no extensive loss of catalytic activity.

**Scheme 39:**
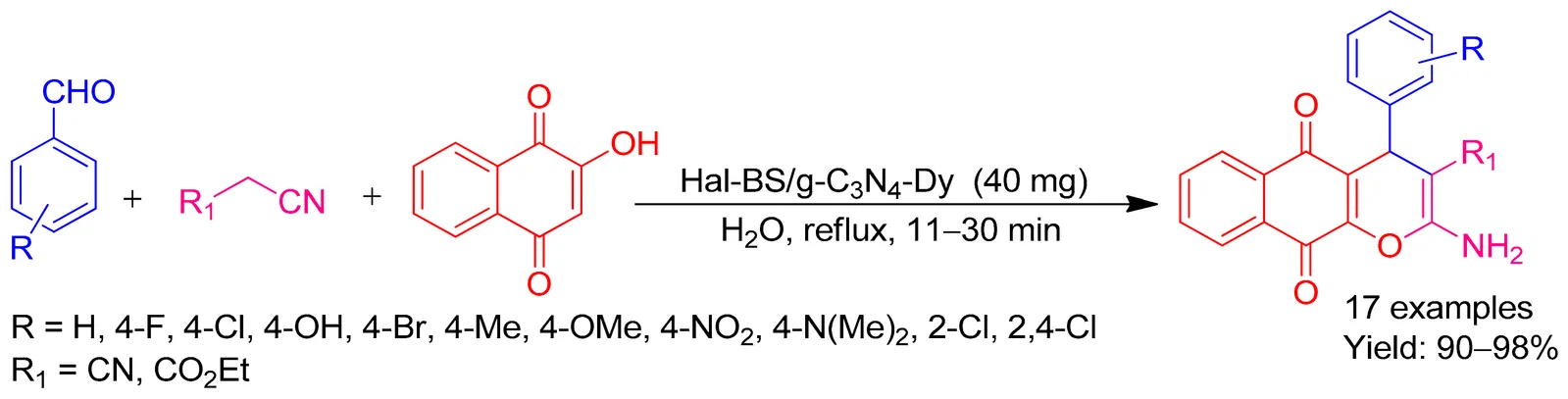
Synthesis of chromenes using Hal-BS/g-C_3_N_4_-Dy catalyst.

In 2022, Rahimzadeh [93] and co-workers reported a dysprosium–balsalazide complex immobilized on functionalized g-C_3_N_4_/halloysite ([Hal-BS/g-C_3_N_4_-Dy]) as a competent catalyst. The catalyst was utilized for the one-pot preparation of chromenes via the reaction of different benzaldehyde, ethyl cyanoacetate/malononitrile, and 2-hydroxy-1,4-naphthoquinone using water in reflux situation, giving the desired products in 90–98% yields within 11–30 min. Moreover, the catalyst was successfully recycled for up to eight consecutive reaction cycles with no substantial loss of activity. The synthetic methodology is illustrated in Scheme 39.

**Scheme 40:**
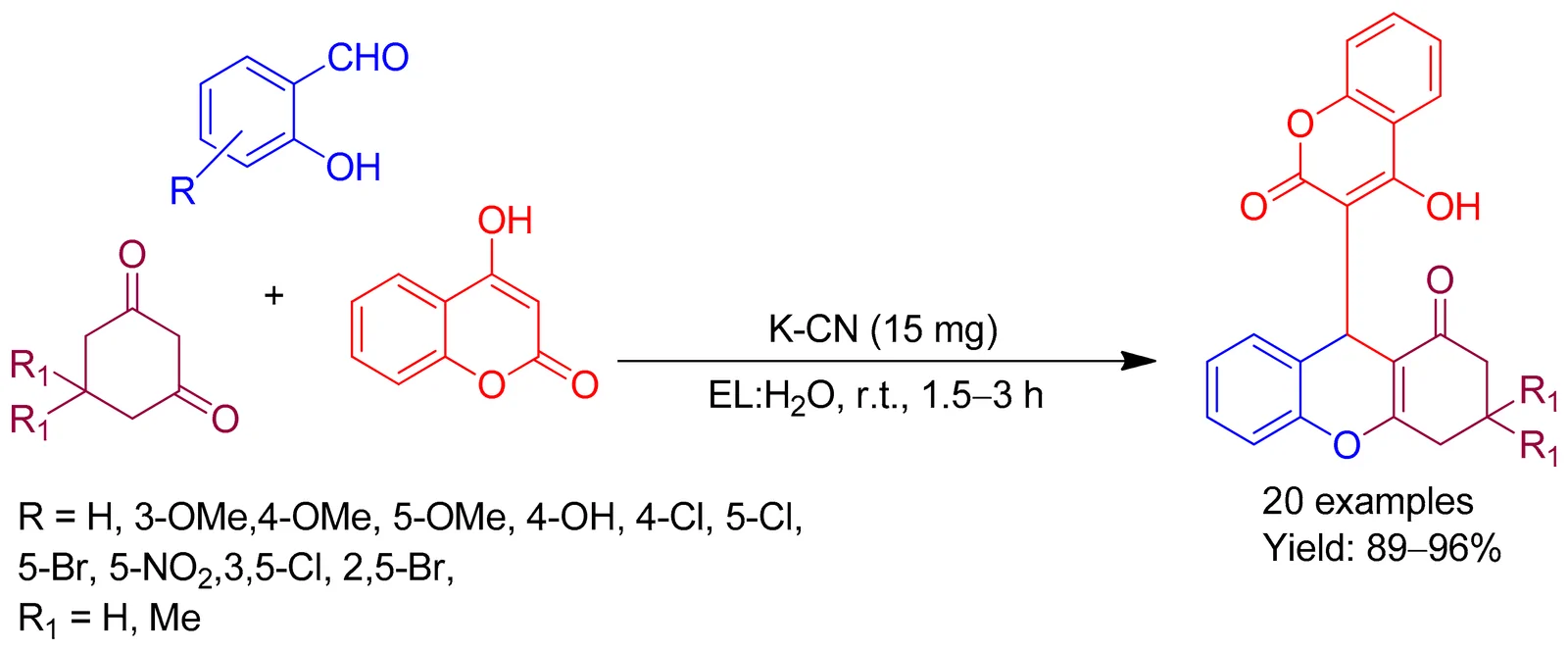
Synthesis of chromenes using K-CN catalyst.

In 2022, Chen [94] and co-workers reported potassium-doped g-C_3_N_4_ (K-CN), prepared via a simple polymerization method, as an effective catalyst for the single-step production of chromene derivatives. The reaction of cyclohexane-1,3-diones, salicylaldehydes, and 4-hydroxycoumarin in H_2_O/ethyl lactate medium at room temperature offered the required products in 89–96% yields within 1.5–3 h (Scheme 40). The catalyst exhibited excellent recyclability and was successfully reused for up to five consecutive reaction cycles without significant loss of activity.

### 4.9. Synthesis of Pyran Derivatives

The pyran analogues form a valuable group of oxygen-containing heterocyclics that exhibit great importance in medicinal chemistry and pharmacy. Pyran nucleus is present in many naturally occurring compounds exhibiting distinct biological actions, including anticancer, antimicrobial, antiviral, anti-inflammatory, antioxidant, and antidiabetic properties [95,96,97].

**Scheme 41:**
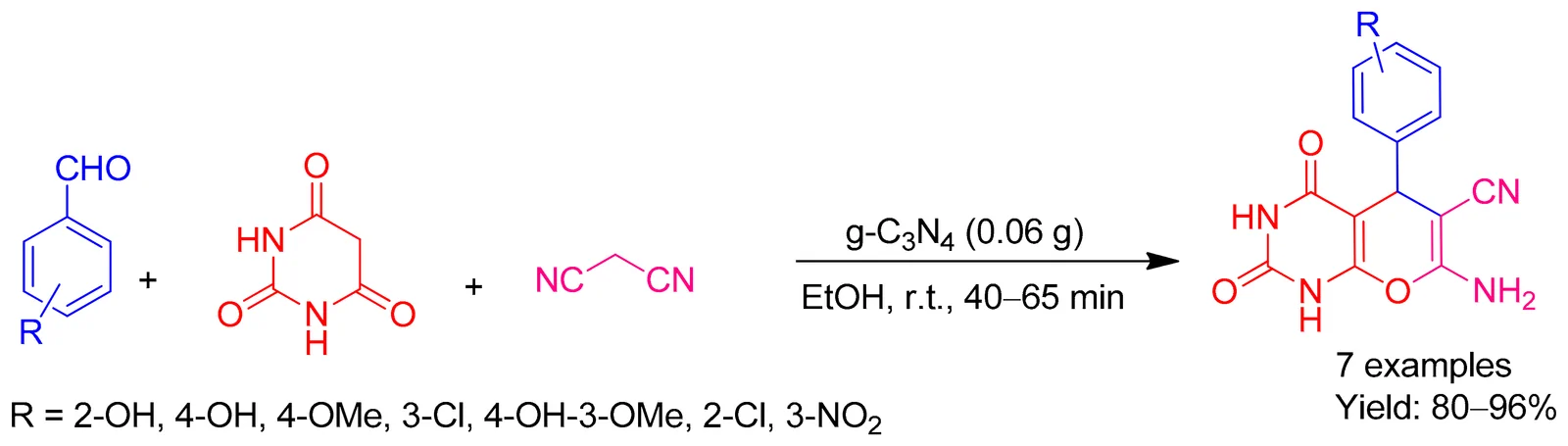
Synthesis of pyrano-pyrimidine derivatives by using g-C_3_N_4_ catalyst.

In 2025, Belambe [98] and co-workers reported GCN (g-C_3_N_4_) as an effective heterogeneous catalyst for the single-step multicomponent preparation of pyrano-pyrimidine derivatives via a Knoevenagel–cyclization sequence. The reaction of malononitrile, various benzaldehydes and barbituric acid in ethanol at room temperature provided the required products in 80–96% yields within 40–65 min. The catalyst showed exceptional recyclability and was successfully reused for up to five repeated reaction cycles without substantial loss of catalytic action. The synthetic methodology is illustrated in Scheme 41.

**Scheme 42:**
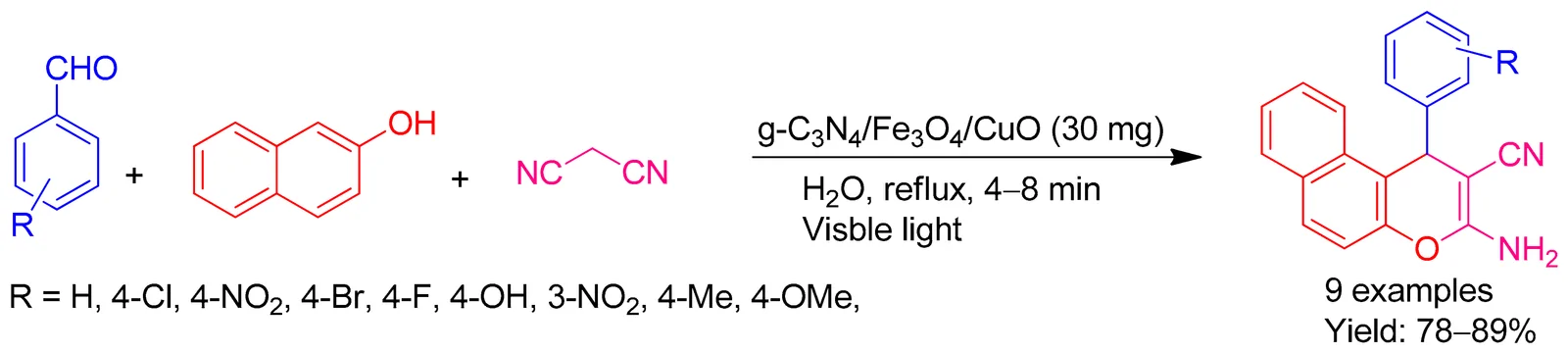
Synthesis of pyran derivatives using g-C_3_N_4_/Fe_3_O_4_/CuO catalyst.

In 2025, Araz Taravati et al. [99] reported a g-C_3_N_4_/Fe_3_O_4_/CuO photocatalyst with dual-functional applications. The heterogeneous photocatalyst efficiently promoted the one-pot synthesis of pyrans via the reaction of 2-naphthol, aromatic aldehydes, and malononitrile under visible-light irradiation and reflux conditions, affording the desired products within 4–8 min with 78–89% yields, as illustrated in Scheme 42. The photocatalyst exhibited excellent recyclability and was reused for five repeated cycles without major loss of catalytic action.

**Scheme 43:**
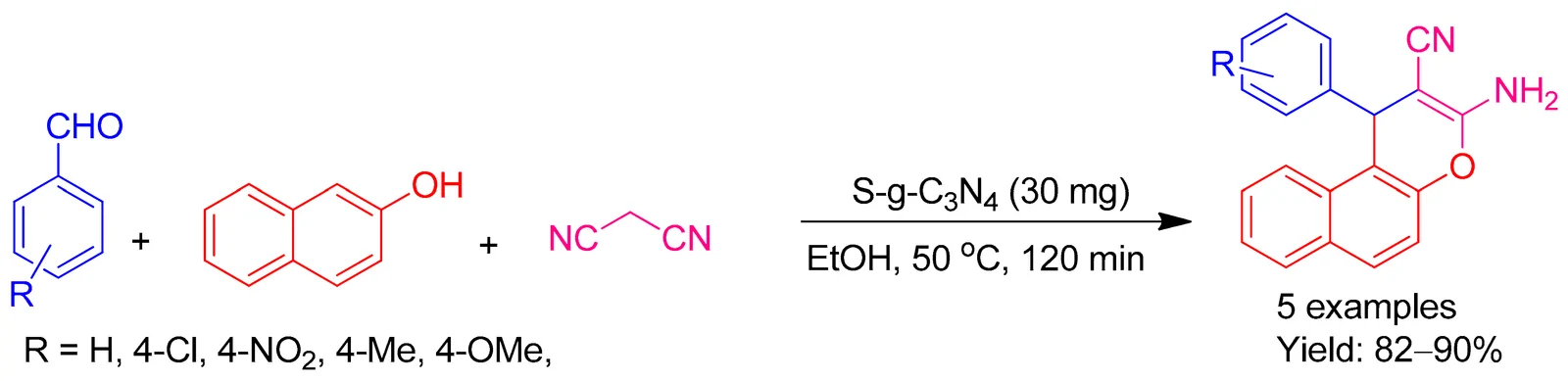
Synthesis of pyran derivatives using S-g-C_3_N_4_ catalyst.

In 2021, Choudhary et al. [100] reported an acid–base bifunctional sulfonated GCN (S-g-C_3_N_4_) catalyst. The catalyst efficiently utilized the sequential tandem reaction of benzaldehydes, active methylene compounds, and 2-naphthol in ethanol at 50 °C, affording the corresponding pyran derivatives within 120 min with 82–90% yields, as illustrated in Scheme 43. The S-g-C_3_N_4_ catalyst was successfully recovered and reused for five consecutive cycles without significant loss of catalytic activity.

**Scheme 44:**
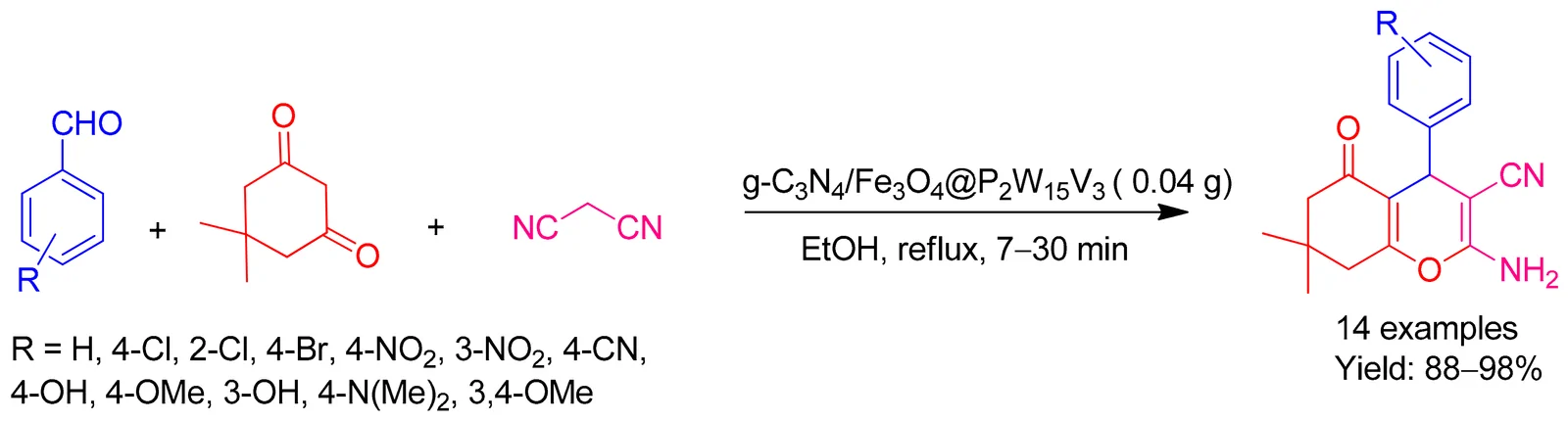
Synthesis of pyran derivatives using g-C_3_N_4_/Fe_3_O_4_@P_2_W_15_V_3_ catalyst.

In 2024, Maleki et al. [101] developed a novel g-C_3_N_4_/Fe_3_O_4_@P_2_W_15_V_3_ magnetic nanocomposite by decorating graphitic carbon nitride with a tri-vanadium-functionalized Dawson-type heteropoly tungstate. The heterogeneous catalyst was effectively used in the single-step preparation of pyran derivatives through the 4CR of benzaldehydes, dimedone, and malononitrile in ethanol under reflux situations. The required products were obtained in good to excellent yields (88–98%) within 7–30 min, as shown in Scheme 44. Furthermore, the catalyst exhibited excellent recyclability and was successfully reused for up to five consecutive reaction cycles with minimal loss of catalytic action.

### 4.10. Synthesis of Spirooxindole Derivatives

The spirooxindoles form a significant group of biologically active heterocycles whose unique structure consists of an oxindole ring fused to an additional one through a spiro junction. The unique 3D structure, rigidity, and chemical diversity of this type of heterocycle make it a very promising molecule in drug discovery and medicinal chemistry. The spirooxindoles demonstrate several biological actions, such as antimicrobial, anticancer, antimalarial, antiviral, anti-inflammatory, and antidiabetic properties [102,103,104].

**Scheme 45:**
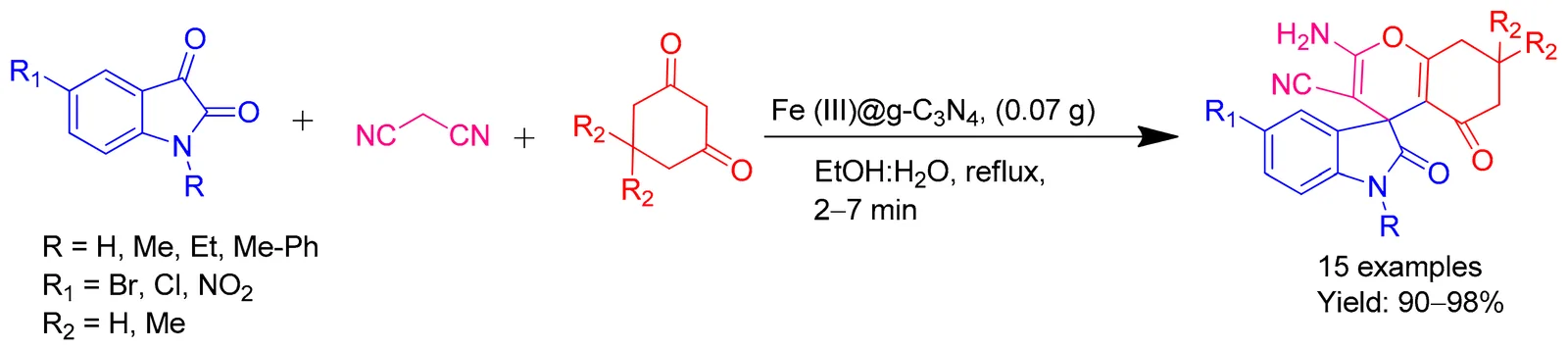
Synthesis of spirooxindole derivatives catalysed by Fe(III)@ g-C_3_N_4._

Allahresani et al. (2019) [105] reported the synthesis of an Fe(III)-supported GCN nanocomposite (Fe(III)@g-C_3_N_4_) via FeCl_3_ impregnation onto g-C_3_N_4_. The catalyst was competently used in multicomponent preparation of spirooxindole derivatives from isatins, 1,3-dicarbonyl compounds, and malononitrile in EtOH/water medium under reflux, offering the desired compounds in excellent yields (90–98%) within 2–7 min, as shown in Scheme 45. Moreover, the catalyst was immediately recovered and reused over several cycles with negligible loss of catalytic activity.

**Scheme 46:**
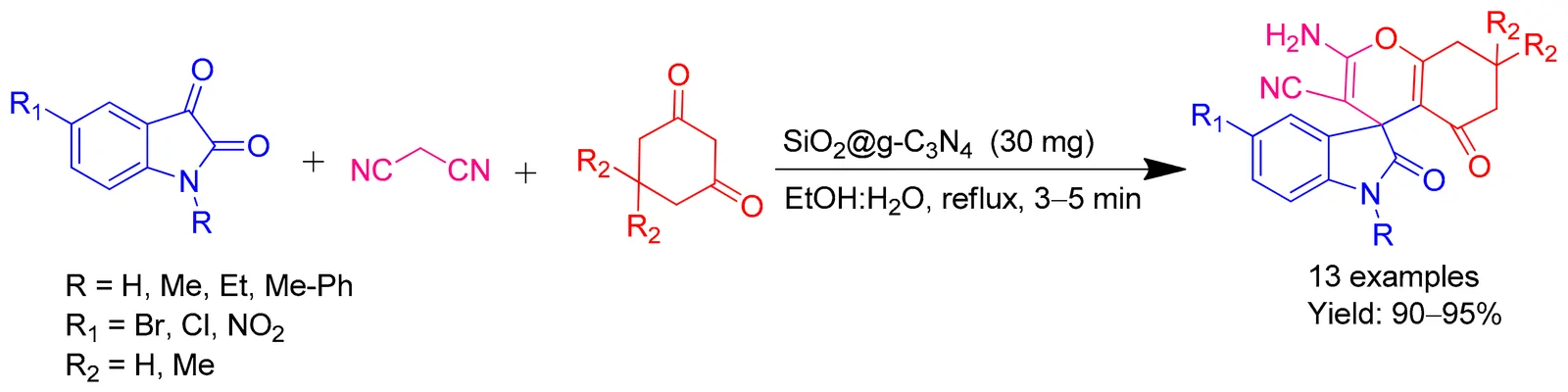
Synthesis of spirooxindole derivatives using a SiO_2_@g-C_3_N_4_ catalyst.

Allahresani et al. (2018) [106] developed a SiO_2_@g-C_3_N_4_ nanocomposite by immobilizing SiO_2_ NPs onto g-C_3_N_4_ nanosheets and demonstrated its efficiency as a heterogeneous catalyst for the one-pot preparation of spirooxindole derivatives. The 3CR of dimedone, isatin, and malononitrile in EtOH/water medium under reflux gave the target products in excellent yields (90–95%) within 3–5 min, as illustrated in Scheme 46. The catalyst also exhibited excellent recyclability, retaining its catalytic performance over several consecutive reaction cycles.

**Scheme 47:**
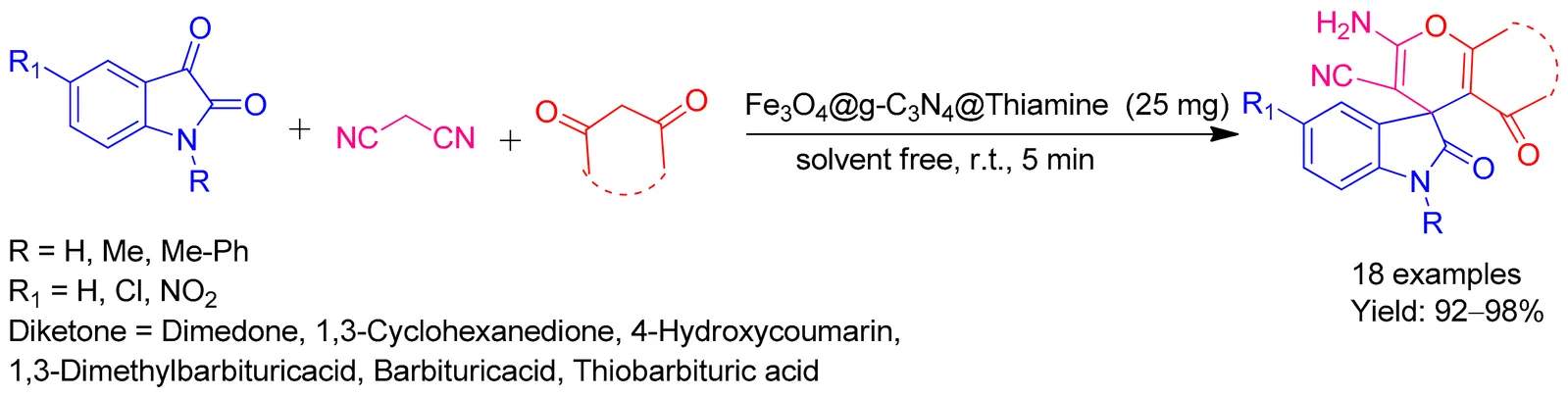
Synthesis of spirooxindole-pyrans using Fe_3_O_4_@g-C_3_N_4_@Thiamine catalyst.

In 2014, Peiman [107] and co-workers reported a novel strategy for the functionalization of Fe_3_O_4_@g-C_3_N_4_ with vitamin B1 (thiamine hydrochloride) using cyanuric chloride (TCT) as a bifunctional covalent linker. The resulting Fe_3_O_4_@g-C_3_N_4_@Thiamine nanocomposite acted as an effectual organocatalyst for the solvent-free preparation of spirooxindoles via the 3CR of malononitrile, 1,3-dicarbonyl compounds, and isatin derivatives. The desired products were acquired in excellent yields (92–98%) within 5 min, as shown in Scheme 47. Moreover, the catalyst was easily recovered and reused for up to seven sequential reaction runs without significant loss of catalytic activity.

**Scheme 48:**
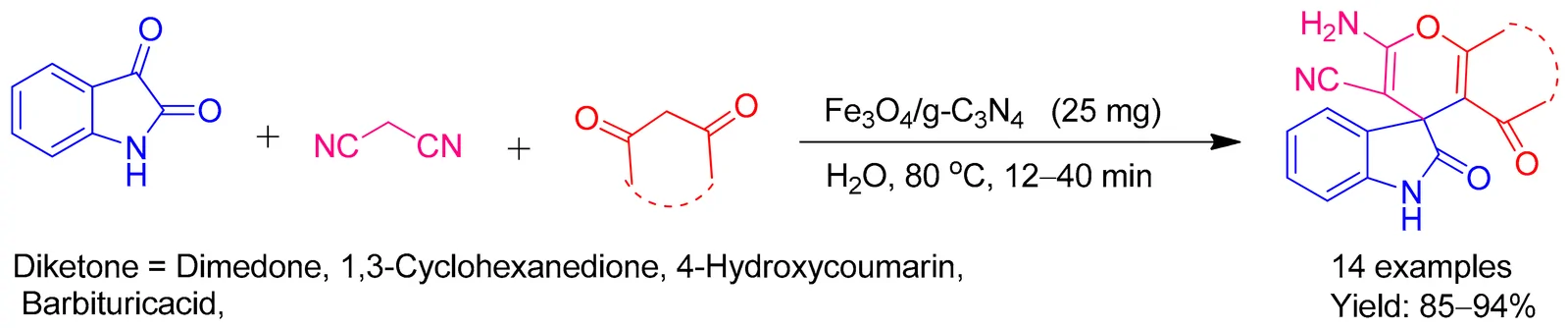
Synthesis of spirooxindole derivatives using Fe_3_O_4_/g-C_3_N_4_ catalyst.

In 2021, Kamali et al. [108] reported an Fe_3_O_4_/g-C_3_N_4_ nanocomposite as an effective and magnetically recoverable catalyst. The catalyst efficiently promoted the preparation of spirooxindole derivatives through the 3CR of isatin, 1,3-diketones and malononitrile in water, affording the desired products in 12–40 min with 85–94% yields, as shown in Scheme 48. The catalyst revealed remarkable recyclability and was reused for six repeated cycles without substantial loss of catalytic action.

### 4.11. Synthesis of Imidazo-Pyridine Derivatives

Imidazo-pyridines are one of the most vital and well-studied series of fused nitrogen heterocycles that have gained attention in medicinal chemistry, pharmaceutical science, and drug discovery due to the variety of biological and pharmacological effects. The fused structure of the imidazole-pyridine system has good electronic effects, hydrogen bonding, and rigidity, which makes this scaffold a useful tool for the development of bioactive compounds. Imidazo-pyridines possess anticancer, antimicrobial, antiviral, anti-inflammatory, antitubercular, and CNS-related effects [109,110].

**Scheme 49:**
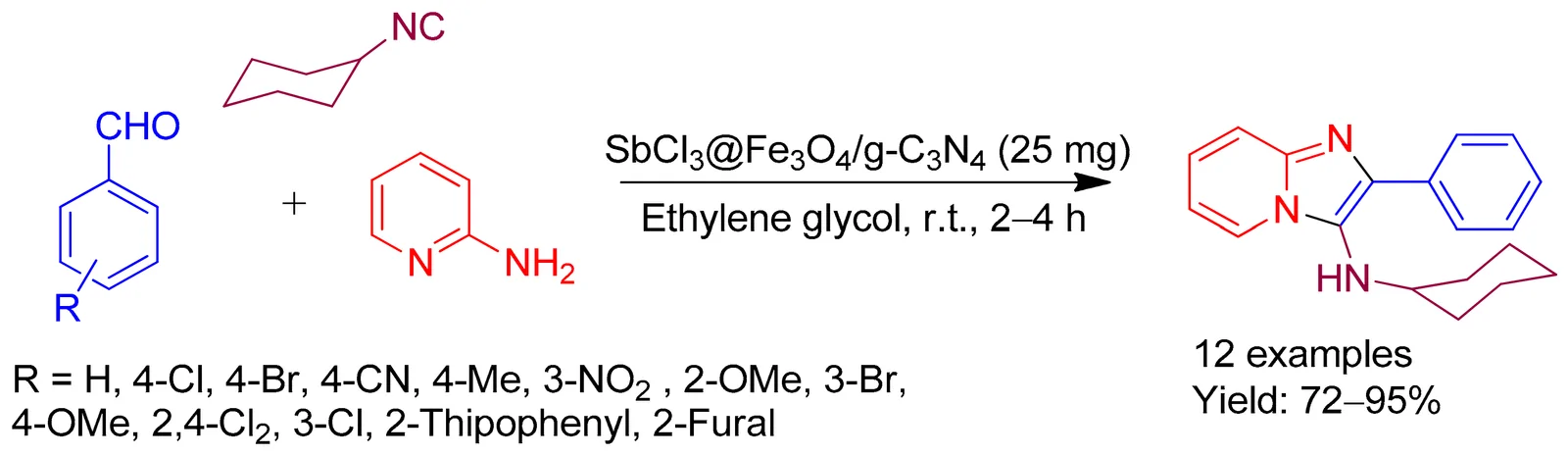
Synthesis of imidazo-pyridine derivatives using the SbCl_3_@Fe_3_O_4_/g-C_3_N_4_ catalyst.

In 2023, Azhdari [111] and co-workers developed an SbCl_3_@Fe_3_O_4_/g-C_3_N_4_ nanocomposite by co-immobilizing magnetic Fe_3_O_4_ nanoparticles and SbCl_3_ onto graphitic carbon nitride nanosheets via a simple solvent evaporation method. The catalyst capably promoted the Groebke–Blackburn–Bienaymé (GBB) MCR between benzaldehyde, 2-aminopyridine, and cyclohexyl isocyanide in ethylene glycol at room temperature, affording imidazo-pyridines in good to excellent yields (72–95%) within 2–4 h, as shown in Scheme 49. Furthermore, the catalyst was readily recovered and reused for at least five successive cycles without any noticeable loss in product yield.

**Scheme 50:**
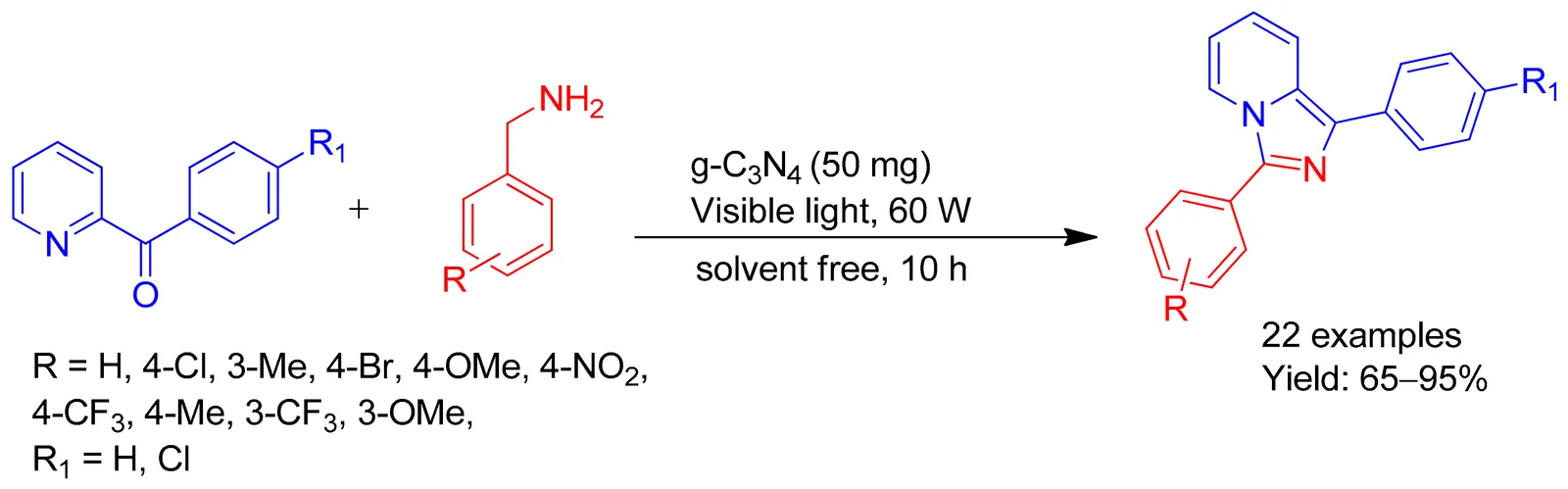
Synthesis of imidazo-pyridines using g-C_3_N_4_ catalyst.

In 2023, Nandish Talpada [112] and co-workers reported GCN (g-C_3_N_4_) as a novel heterogeneous photocatalyst for the visible-light-mediated synthesis of imidazo-pyridines. The protocol involved the reaction of benzylamine with 2-benzoyl pyridine under solvent-free circumstances and visible-light irradiation, affording a wide range of imidazo-pyridine derivatives in 10 h with excellent yields of 65–95%, as demonstrated in Scheme 50. The g-C_3_N_4_ catalyst revealed excellent recyclability and was successfully reused for five repeated runs without any substantial loss of catalytic action.

### 4.12. Synthesis of Xanthene Derivatives

Xanthene analogues constitute an essential family of heterocyclic oxygen-containing aromatic compounds with substantial importance in medicinal chemistry, pharmaceutical studies, and functional materials. The xanthene ring is found in several biologically active compounds and displays varied pharmacological effects, namely, anticancer, antimicrobial, antiviral, anti-inflammatory, antioxidant, and antimalarial effects. In addition to these biological effects, xanthene analogues have received attention for applications as fluorescent dyes and optical materials due to their unique photophysical behaviour [113,114].

**Scheme 51:**
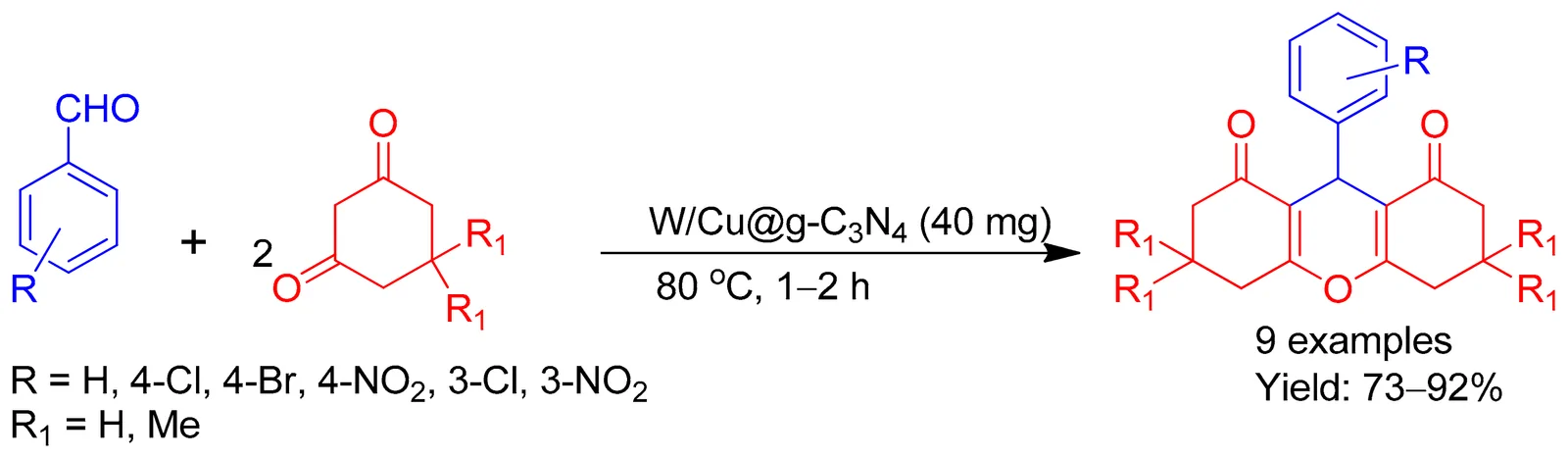
Synthesis of xanthene derivatives using W/Cu@g-C_3_N_4_ catalyst.

In 2023, Azizi et al. [115] developed a novel WCl_6_/CuCl_2_-supported GCN (W/Cu@g-C_3_N_4_) heterogeneous catalyst via a hydrothermal method. The catalyst was effectively used in solvent-free preparation of xanthene derivatives through the condensation of various benzaldehydes with dimedone at 80 °C. The reactions were completed within 1–2 h, offering the desired compounds in good to excellent yields (73–92%), as illustrated in Scheme 51. Furthermore, the catalyst exposed exceptional recyclability and was successfully reused for up to five consecutive cycles with no major loss of catalytic action.

**Scheme 52:**
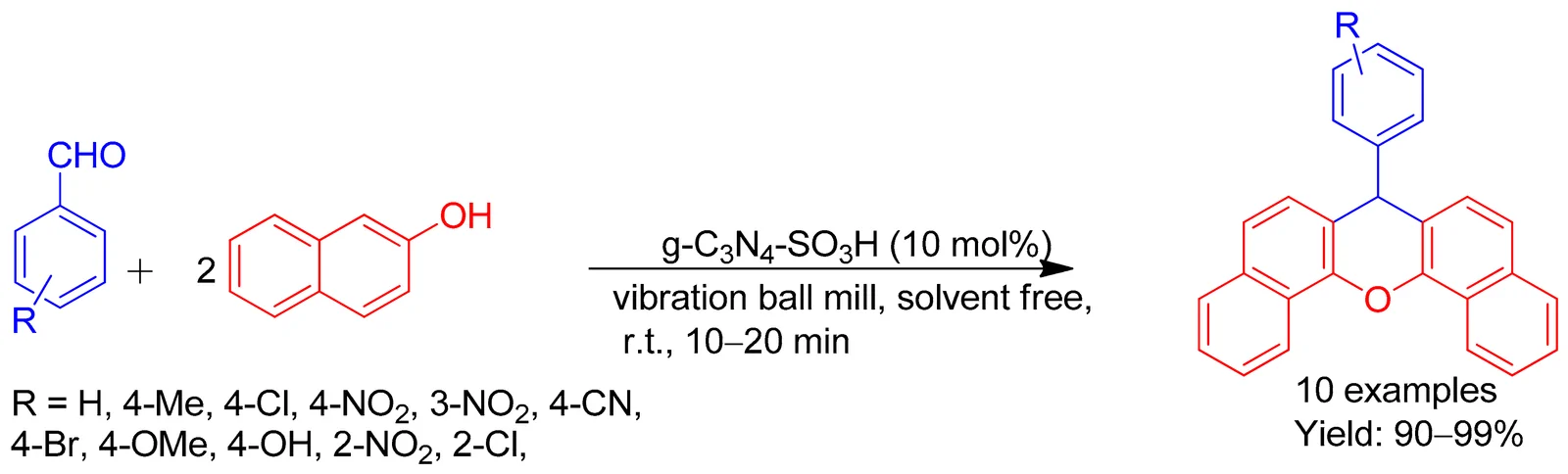
Synthesis of benzo-xanthene derivatives using Sg-CN catalyst.

In 2021, Qareaghaj et al. [116] reported sulfonated GCN (Sg-CN) as an efficient heterogeneous acidic catalyst for the mechanochemical synthesis of xanthene derivatives. The catalyst helped the solvent-free condensation of β-naphthol and various aldehydes under ball-milling situations at room temperature, offering the required products in excellent yields (90–99%) within 10–20 min, as shown in Scheme 52. Furthermore, the Sg-CN catalyst was successfully recovered and reused for up to three repeated reaction cycles with minimal loss of catalytic activity.

### 4.13. Synthesis of Propargylamine Derivatives

Propargylamine derivatives are a wide class of nitrogen-based compounds characterized by significant attention in organic synthesis, medicinal chemistry, and pharmaceutical studies owing to their distinctive features of having both amine groups and alkyne groups in one molecule. Propargylamine derivatives and their derivatives have shown varied biological activity such as antimicrobial, anticancer, antiviral, anti-inflammatory, and neuroactivity [117,118].

**Scheme 53:**
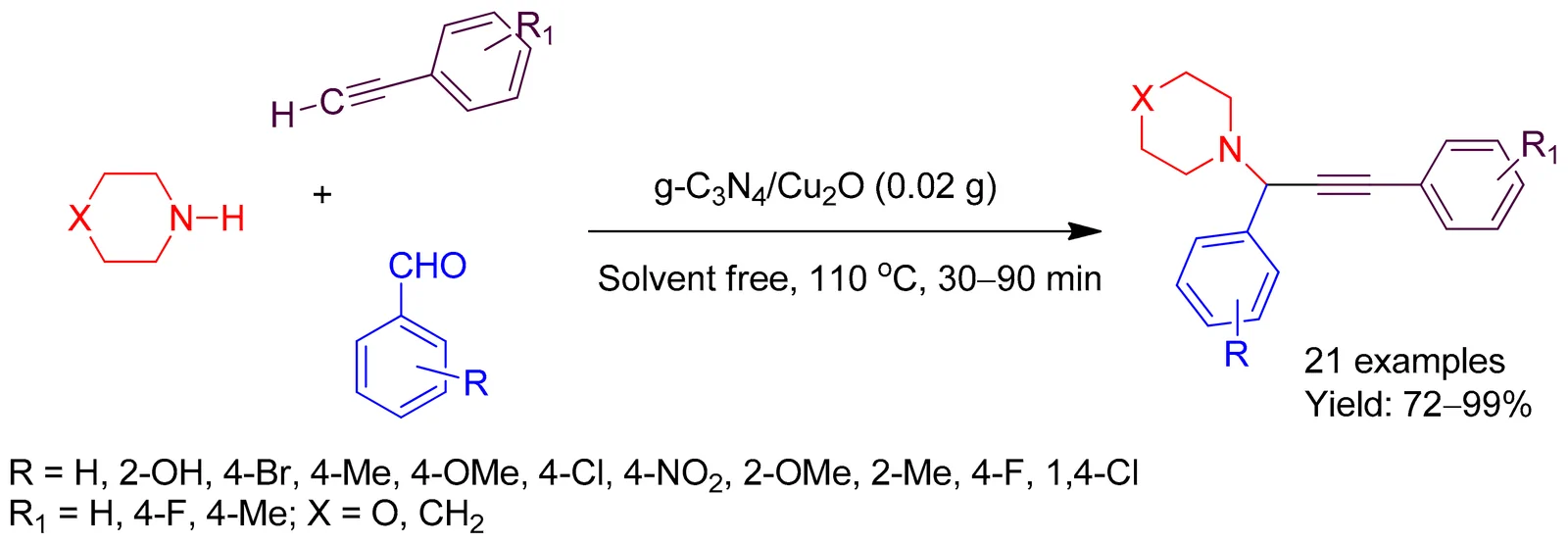
Synthesis of propargylamine derivatives using Cu_2_O@g-C_3_N catalyst.

In 2019, Karkeabadi et al. [119] reported a Cu_2_O-modified GCN (Cu_2_O@g-C_3_N_4_) catalyst prepared by the one-step thermal polycondensation of urea followed by Cu(I) oxide modification. The catalyst was competently used in the A^3^ coupling reaction of alkynes, secondary amines and aryl aldehydes to synthesize a wide range of propargylamine derivatives in 30–90 min with 72–99% yields, as shown in Scheme 53. The catalyst was reused for four repeated cycles with only minimal loss of catalytic activity.

**Scheme 54:**
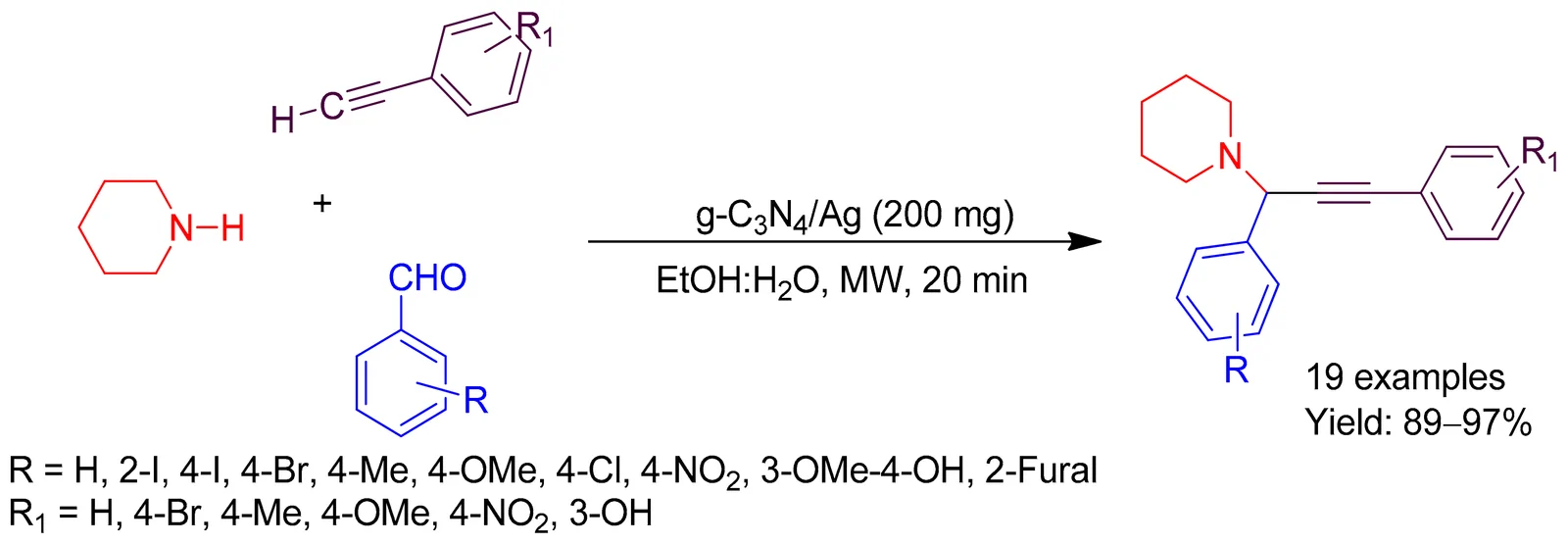
Synthesis of propargylamine derivatives using AgNPs@g-C_3_N_4_ catalyst.

In 2018, Patel [120] and associates reported silver NPS supported on GCN (AgNPs@g-C_3_N_4_) as an effective catalyst for the microwave-assisted A^3^ coupling reaction. The catalyst promoted the reaction of benzaldehydes, terminal alkynes and secondary amines in water to provide propargylamine derivatives in excellent yields of 89–97% within 20 min. The catalyst also showed excellent recyclability and was reused for up to seven repeated reaction cycles without significant loss of action. The synthetic methodology is illustrated in Scheme 54.

### 4.14. Synthesis of Thiazolidine Derivatives

Thiazolidine derivatives form a key group of heterocyclic compounds containing sulphur and nitrogen atoms that have drawn immense interest in medicinal chemistry, pharmaceutical sciences, and drug development due to their multiple biological applications. The thiazolidine framework has been known as a vital pharmacophoric unit exhibiting antibacterial, antifungal, anticancer, anti-inflammatory, antiviral, antitubercular, and antidiabetic properties [121,122].

**Scheme 55:**
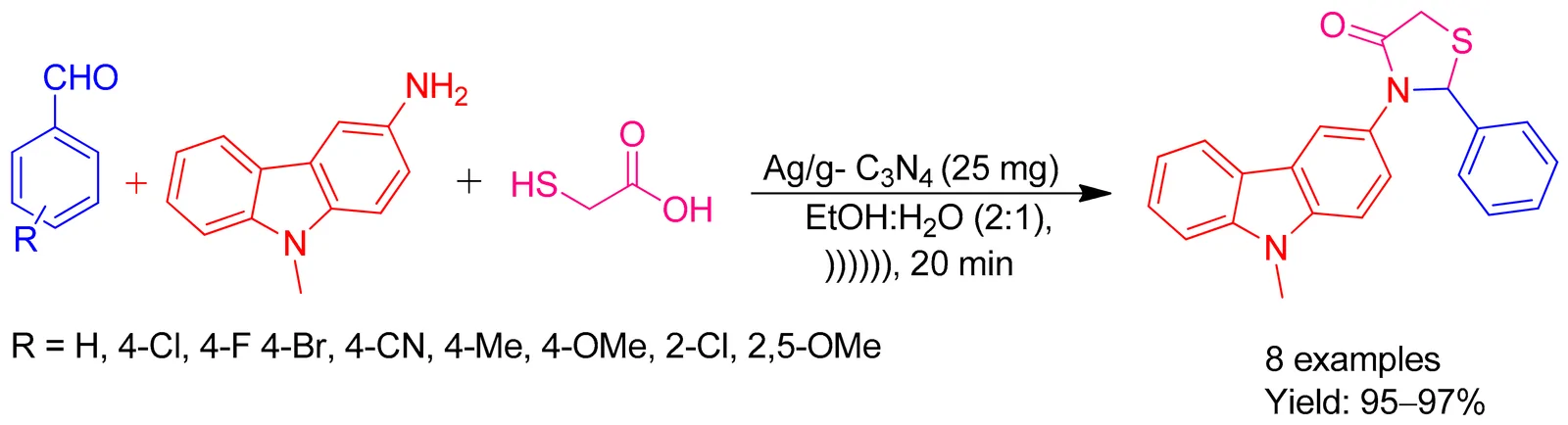
Synthesis of thiazolidinone derivatives using Ag/g-C_3_N_4_ catalyst.

In 2025, Bhabal [123] and co-workers reported Ag/g-C_3_N_4_, featuring uniformly dispersed Ag NPs on the g-C_3_N_4_ surface, as an effective heterogeneous catalyst for the ultrasonic-assisted preparation of thiazolidinone derivatives. The reaction of 3-amino-9-ethylcarbazole, aromatic aldehydes, and thioglycolic acid in ethanol provided the desired products in 95–97% yields within 20 min. The catalyst was readily recovered and reused for up to five repeated runs with no major loss of activity. The synthetic methodology is illustrated in Scheme 55.

**Scheme 56:**
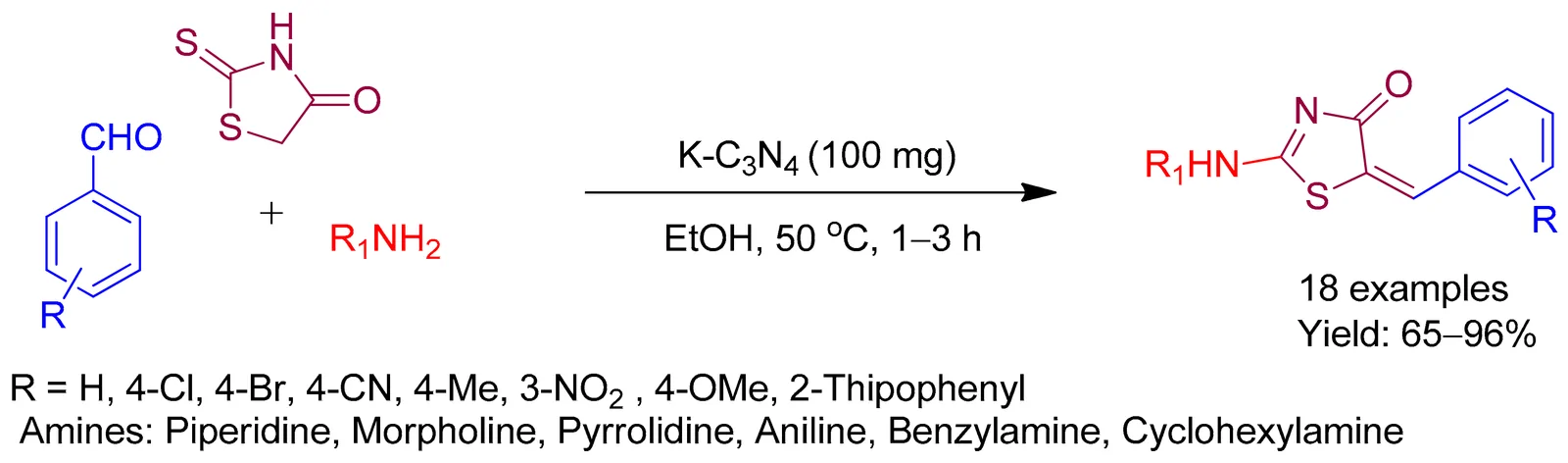
Synthesis of thiazolone derivatives using K-g-C_3_N_4_ catalyst.

In 2022, Azizi et al. [124] reported the synthesis of K-g-C_3_N_4_ nanotubes via the thermal polymerization of melamine followed by alkaline exfoliation. Owing to their porous ionic framework and abundant basic nitrogen sites, the nanotubes served as an effective catalyst for the one-pot 3CR of thiazolone derivatives from aromatic aldehydes, rhodanine and amines, in ethanol. The reactions gave the required products in good to excellent yields (65–96%) within 60–180 min, as shown in Scheme 56. Moreover, the catalyst was successfully recovered and reused for up to five repeated cycles with no major loss of catalytic action.

### 4.15. Synthesis of Pyrrole Derivatives

The derivatives of pyrroles constitute a vital category of heterocycles containing nitrogen atoms and having widespread applications in drug discovery and design, organic natural product syntheses, and materials science. The pyrrole ring system is found in many biological entities and possesses various pharmacological effects, such as antitumor, antimicrobial, antiviral, anti-inflammatory, antioxidant, and antitubercular activity [125,126].

**Scheme 57:**
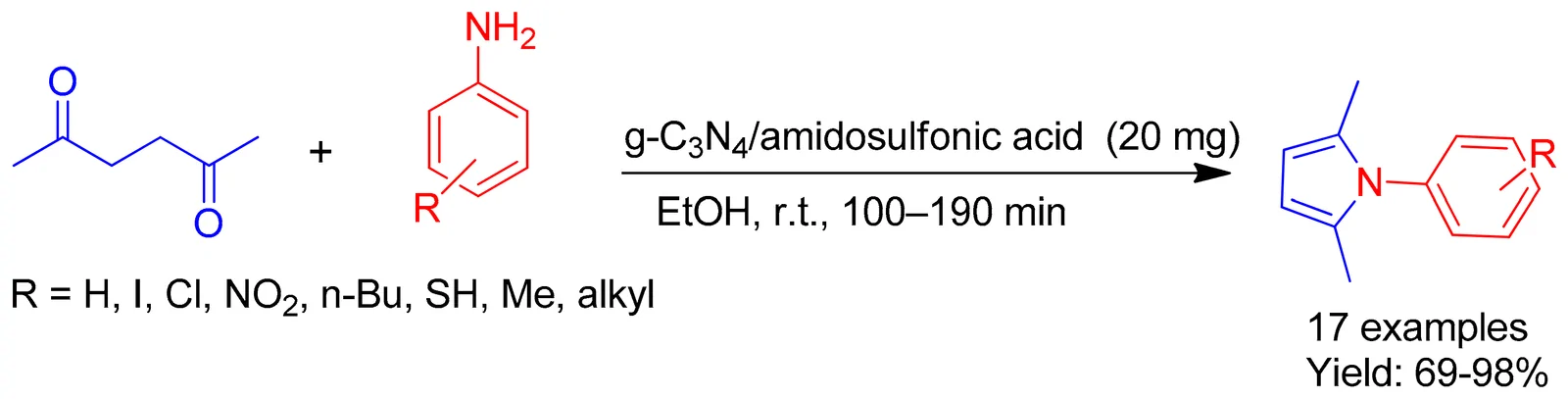
Synthesis of substituted pyrroles using g-C_3_N_4_/amidosulfonic acid catalyst.

In 2021, Azhdari et al. [127] reported an acidic catalyst prepared by the in situ immobilization of NH_2_SO_3_H (amidosulfonic acid) on GCN (g-C_3_N_4_) under hydrothermal conditions. The catalyst efficiently promoted the synthesis of pyrroles via the reaction of substituted anilines with 2,5-hexanedione in ethanol at room temperature, giving the desired compounds in 100–190 min with excellent yields of 69–98%, as illustrated in Scheme 57. The catalyst exhibited excellent stability and was reused for five consecutive cycles with only a slight decline in catalytic action.

### 4.16. Synthesis of Coumarin Derivatives

Coumarin derivatives are a valuable group of oxygen-containing heterocyclic compounds that hold much significance in areas such as medicinal chemistry, pharmaceutical sciences, natural products chemistry, and materials chemistry. Coumarins exist in nature in various plant-based compounds and show numerous biological activities, namely, anticancer, anti-microbial, anti-inflammatory, antioxidant, antiviral, anti-coagulant, and antidiabetic [128,129].

**Scheme 58:**
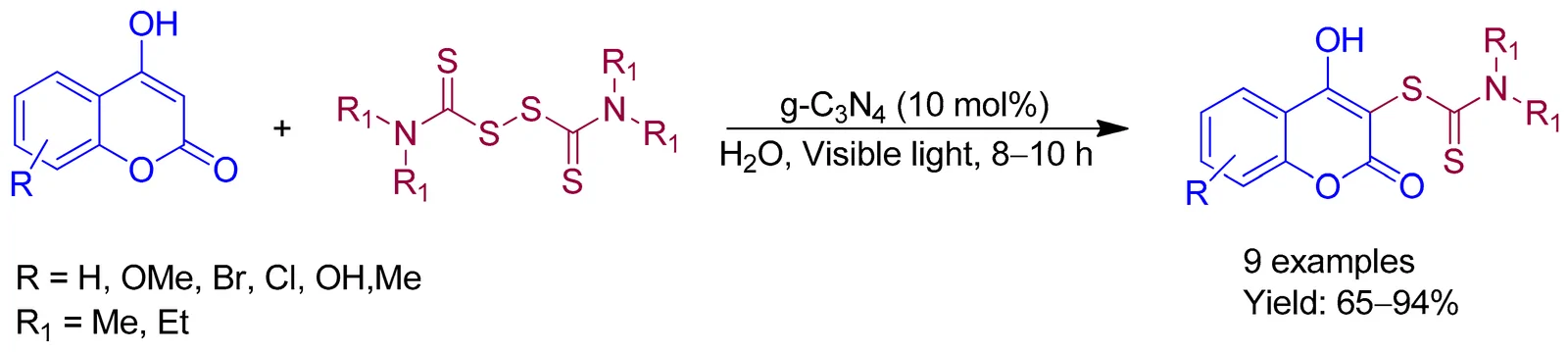
Synthesis of functionalized coumarin derivatives using g-C_3_N_4_ catalyst.

In 2025, Pandya [130] and co-workers reported a metal-free g-C_3_N_4_ photocatalyst for the visible-light-driven regioselective sulfenylation of 4-hydroxycoumarin derivatives. The reaction involved the coupling of 4-hydroxycoumarins with tetraalkyl thiuram disulfides in water under visible-light irradiation, affording the corresponding coumarin derivatives in 65–94% yields within 8–10 h. The protocol avoided the use of transition metals and demonstrated the effectiveness of g-C_3_N_4_ as a recyclable heterogeneous photocatalyst under mild conditions. The synthetic methodology is illustrated in Scheme 58.

### 4.17. Synthesis of Acridine Derivatives

Acridines constitute a significant family of nitrogenous fused heterocycles which have been extensively studied in the field of pharmaceutical sciences, medicinal chemistry, and materials science due to their unique electronic and photophysical characteristics along with their varied biological activities which include anticancer, antimicrobial, antimalarial, antiviral, and antiparasitic effects [131,132].

**Scheme 59:**
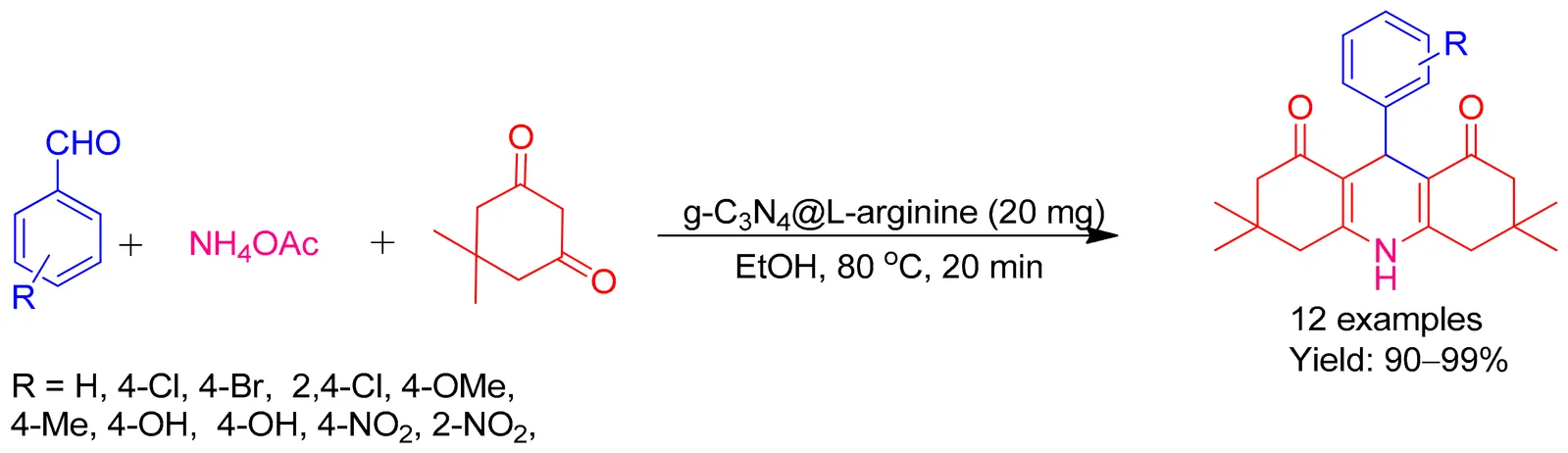
Synthesis of acridine derivatives using g-C_3_N_4_@L-arginine.

In 2022, Bijari [133] and colleagues developed GCN-supported L-arginine (g-C_3_N_4_@L-arginine) as an effective catalyst for the one-pot preparation of acridine derivatives. The reaction of dimedone, aromatic benzaldehydes, and NH_4_OAc in ethanol at 80 °C afforded the desired compounds in 90–99% yields within 20 min (Scheme 59). The catalyst was readily recovered and reused for five consecutive cycles without substantial loss of action.

### 4.18. Synthesis of Phenazines

Phenazine derivatives are a significant class of nitrogen-containing fused heterocyclics that have attracted considerable interest in medicinal chemistry, pharmaceutical research, and materials science owing to their unique redox and electronic properties. The phenazine framework is found in several naturally occurring bioactive compounds and exhibits varied biological actions, including antifungal, antimicrobial, antiviral, anticancer, and anti-inflammatory effects [134,135].

**Scheme 60:**
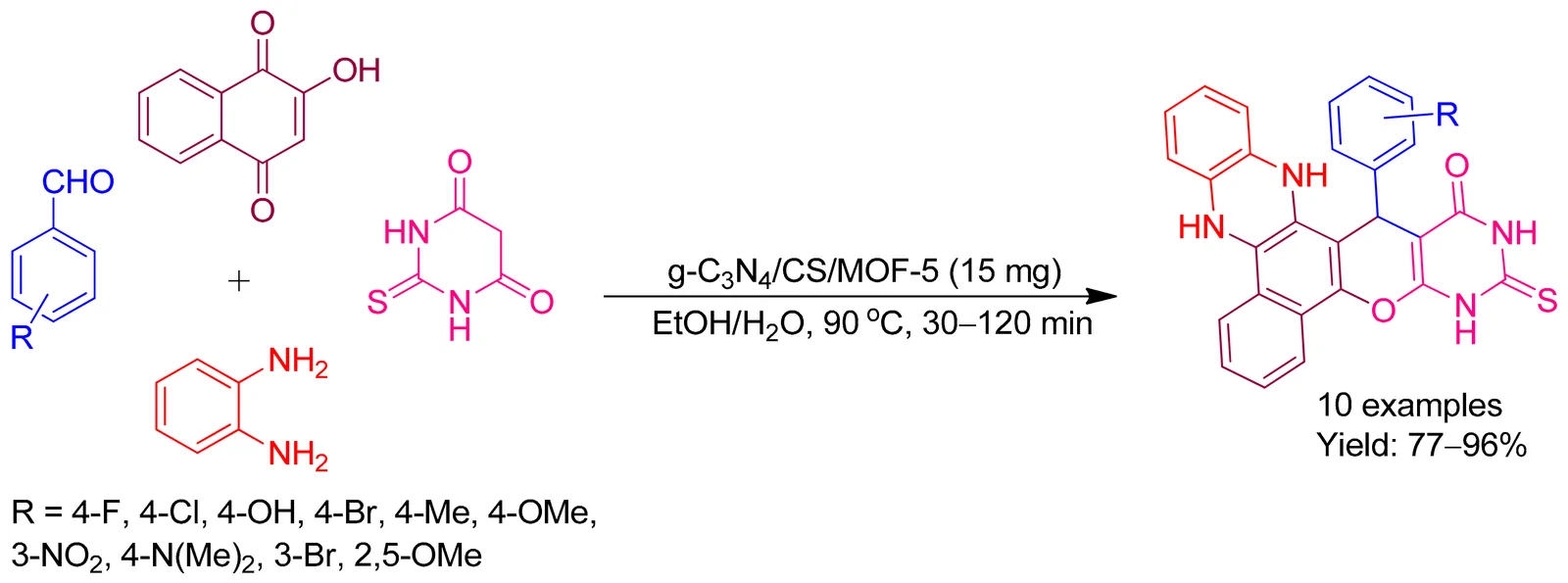
Synthesis of pyrano-phenazines using g-C_3_N_4_/CS/MOF-5 catalyst.

In 2026, Atefeh Zeinali [136] and co-workers reported a biocompatible g-C_3_N_4_/CS/MOF-5 nanocomposite as an effective catalyst for the one-pot preparation of pyrano-phenazine derivatives. The reaction of thiobarbituric acid, *o*-phenylenediamine, benzaldehydes, and 2-hydroxy-1,4-naphthoquinone in an aqueous ethanol medium at 90 °C afforded the required products in excellent yields (77–96%) within 30–120 min. The catalyst exhibited excellent recyclability and was successfully reused for up to six repeated reaction cycles without substantial loss of catalytic action. The synthetic methodology is illustrated in Scheme 60.

### 4.19. Synthesis of Epoxide Derivatives

The epoxide family represents an essential group of strained three-membered cyclic ethers which are widely used in synthetic, medicinal, pharmaceutical and materials chemistry. The presence of an active three-membered ring makes epoxides a versatile reagent to be involved in various regio- and stereoselective reactions which makes them a useful tool for heterocyclic and biologically active compounds preparation [137,138].

**Scheme 61:**
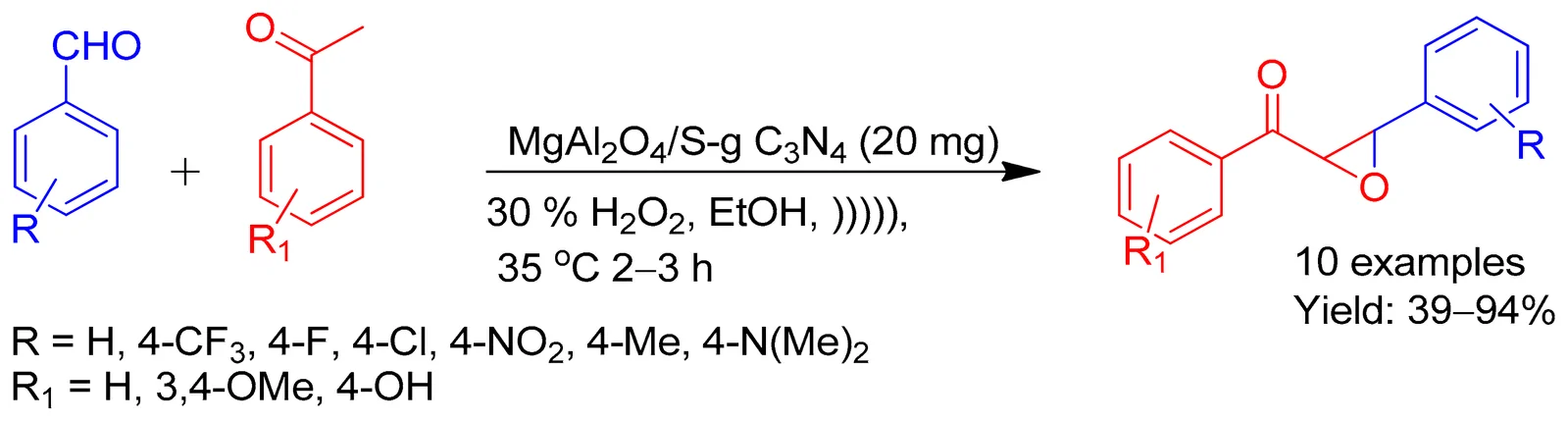
Synthesis of epoxide derivatives using MgAl_2_O_4_/S-g C_3_N_4_ catalyst.

In 2026, Bhanderi [139] and colleagues developed a series of MgAl_2_O_4_/S-g-C_3_N_4_ composite heterogeneous catalysts by incorporating MgAl_2_O_4_ onto sulphur-doped graphitic carbon nitride nanosheets. The catalysts exhibited enhanced activity for the ultrasonic-assisted one-pot preparation of epoxide derivatives, from benzaldehyde and acetophenone derivatives in the existence of H_2_O_2_ in ethanol (Scheme 61). The desired products were obtained within 2–3 h in moderate to excellent yields (39–94%). Furthermore, the MgAl_2_O_4_/S-g-C_3_N_4_ catalyst was readily recovered and successfully reused for up to five consecutive reaction cycles with minimal loss of catalytic activity.

### 4.20. Synthesis of Aminonitrile Derivatives

Aminonitriles are important molecules that possess two different functionalities, namely, the amino functionality and nitrile functionality. As such, these molecules can be regarded as bifunctional molecules with great applications in synthetic chemistry and medicine. Due to their complementary nature, they can undergo various chemical reactions that lead to products such as amino acids and amides [140,141].

**Scheme 62:**
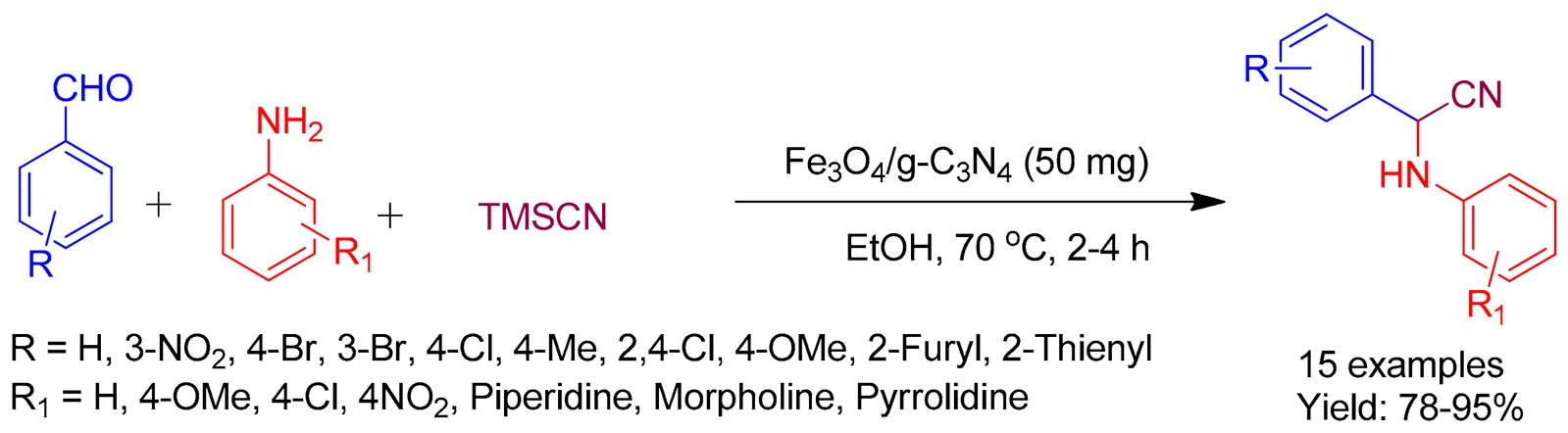
Synthesis of α-aminonitriles using Fe_3_O_4_/g-C_3_N_4_ catalyst.

In 2018, Azizi et al. [142] reported an Fe_3_O_4_/g-C_3_N_4_ nanocomposite as an effective heterogeneous catalyst. The catalyst progressed the 3CR of amines, carbonyl compounds, and trimethylsilyl cyanide (TMSCN) in ethanol, affording the corresponding α-aminonitriles within 2–4 h with 78–95% yields, as shown in Scheme 62. The catalyst exhibited excellent recyclability and was reclaimed for five consecutive cycles without substantial loss of catalytic action.

Table 2 summarizes the preparation and structural modification strategies employed for g-C_3_N_4_-based catalysts in the reported reactions. The table highlights the different approaches used to tailor the structural, optical, and surface properties of g-C_3_N_4_ for improved catalytic performance, which are presented for comparison.

## 5. Green and Sustainable Aspects of g-C_3_N_4_-Catalysed MCRs

The sustainability of the multicomponent reactions catalysed by g-C_3_N_4_ can be analysed in terms of green chemistry metrics such as reaction mass efficiency (RME), atom economy (AE), E-factor, process mass intensity (PMI), carbon efficiency (CE), energy efficiency, catalyst loading, and recyclability, as is summarized in Table 3. The intrinsic feature of MCRs to occur as one-pot reactions lowers the number of synthetic stages, isolation, purification steps, solvent usage, and waste production, hence providing the reactions with improved environmental performance. The nitrogen-enriched and heterogeneous nature of g-C_3_N_4_ allows for efficient catalysis at relatively low temperatures, while the possibility of easy separation and reuse of the catalyst can decrease the amount of catalyst used. High yields of products, short reaction time, low catalyst loading, and the use of environmentally friendly solvents, e.g., water, ethanol, and aqueous ethanol, provide further improvement in the reaction mass efficiency and reduction of E-factor. Moreover, microwave irradiation, ultrasound, visible-light irradiation, and mechanochemical activation can decrease the reaction time and lower energy requirements.

More importantly, the greening of these reactions should not only be evaluated based on yield alone. The use of solvents and reagents, catalyst preparation, energy consumption, purification procedures, and catalyst recyclability should also be taken into consideration. AE and CE represent an effective utilization of the atoms from the reactants and carbon into the product, while RME, PMI, and E-factor are better representations of real material consumption and waste production. The ability of the catalyst to be recyclable is especially beneficial in g-C_3_N_4_ catalysts, as there are some catalysts that can retain their catalytic activity through several cycles. Nevertheless, the quantification of green metrics is lacking in numerous publications. Hence, future studies should include the evaluation of the following along with the yield: reaction time, choice of solvent, and recyclability of the g-C_3_N_4_-catalysed MCRs.

Green metrics provide a systematic means of assessing the environmental and resource efficiency of g-C_3_N_4_-catalysed synthetic processes. The commonly applied parameters, including atom economy, reaction mass efficiency, energy efficiency, catalyst loading, and recyclability, are summarized in Table 4.

## 6. Mechanistic Insights of Multicomponent Reactions (MCRs) Catalysed by Graphitic Carbon Nitride

GCN (g-C_3_N_4_) is considered one of the most effective metal-free heterogeneous catalysts for MCRs due to its specific surface properties and electronic characteristics. The catalyst is a π-conjugated system consisting of heptazine units that are bound to each other via nitrogen atoms, which provides numerous Lewis base nitrogen sites along with weak Brønsted acidic surface amino (–NH and –NH_2_) groups. It allows the implementation of acid–base cooperation between the catalyst’s sites, thus enabling simultaneous activation of electrophilic and nucleophilic molecules. Furthermore, the porous layered structure and high conjugation of the surface make it possible to effectively adsorb the reacting molecules via hydrogen and π-π interactions and increase the concentration of the adsorbed reactants. Thus, MCRs proceed rapidly under mild reaction conditions without any environmental contamination.

Usually, the catalytic reaction starts with adsorption of the molecules onto the surface of the catalyst. Carbonyl compounds are activated via hydrogen bonding with the surface amino groups to make the carbonyl carbon more electrophilic, while nucleophilic molecules are activated by Lewis-basic nitrogen atoms to abstract the protons or increase nucleophilicity [143]. The mutual activation facilitates the creation of suitable intermediates by way of Knoevenagel condensation, Schiff base (imine) formation, or other types of condensation, depending on the type of reactants. These intermediates further undergo Michael addition or nucleophilic addition with the third reacting element to give rise to very well-functionalized intermediates.

**Figure 2:**
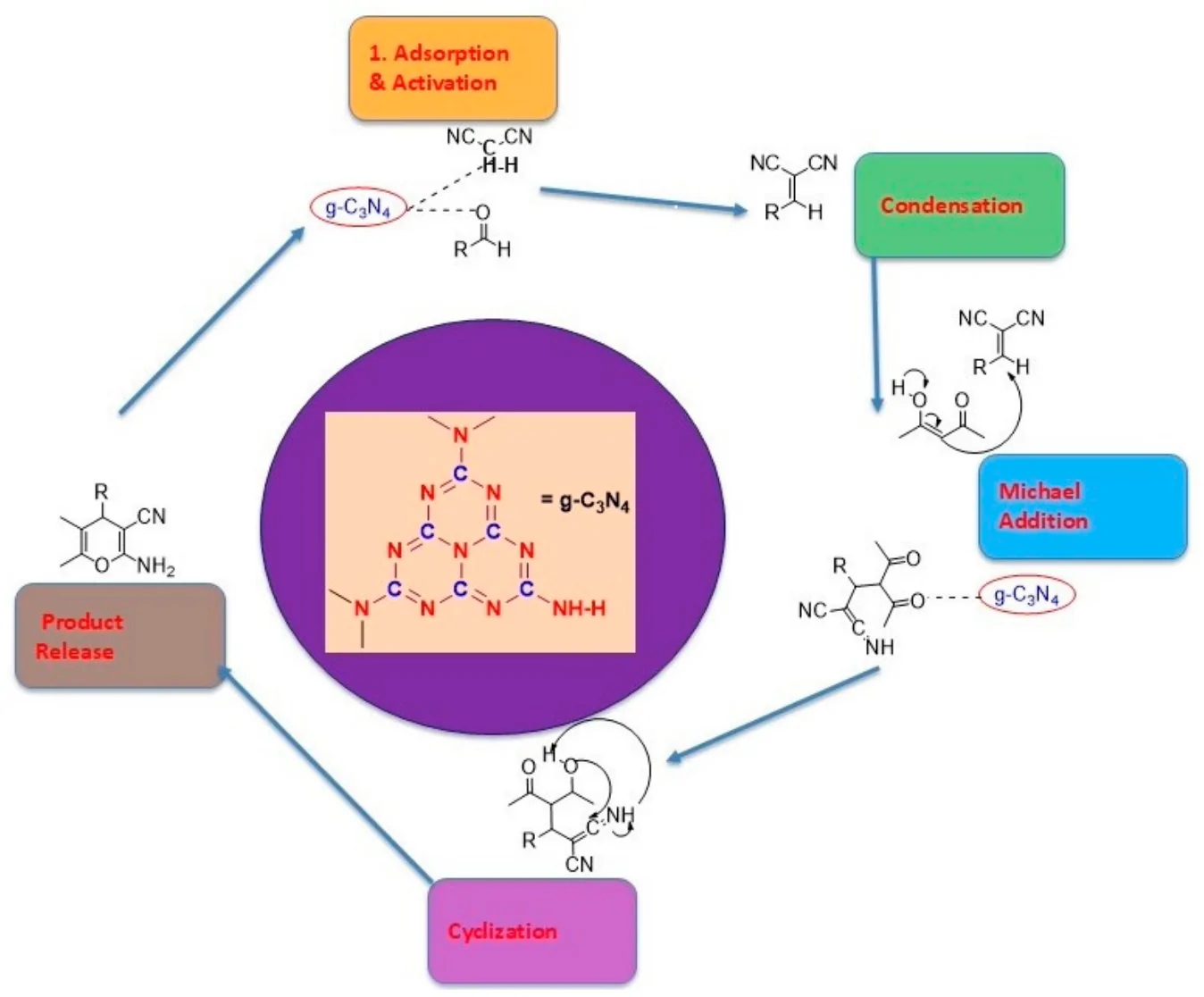
Mechanism of g-C_3_N_4_-catalysed MCRs.

The heterogenous catalyst makes the intermediates more stable and reduces the activation energy of subsequent reactions to increase the speed and specificity of reactions. Once the intermediates have been created, the next step involves intramolecular cyclization of intermediates to create the heterocyclic ring, followed by dehydration, aromatization, or tautomerization for the creation of heterocyclic products [144,145], and this is graphically represented in Figure 2.

The mechanistic behaviour of g-C_3_N_4_-catalysed multicomponent reactions (MCRs) depends on the catalyst structure, reaction conditions, and presence or absence of light. Accordingly, the reported mechanisms can be broadly classified into conventional thermal/acid–base, photocatalytic, radical/oxidative, and hybrid-catalyst pathways.

Conventional thermal/acid–base pathway: In the absence of irradiation, the nitrogen-rich framework of g-C_3_N_4_ provides Lewis-basic nitrogen sites, surface amino groups, hydrogen-bonding interactions, and adsorption sites for reactants. These surface functionalities can activate carbonyl compounds and nucleophilic partners, facilitating Knoevenagel condensation or imine formation, followed by Michael/nucleophilic addition, cyclization, dehydration, and aromatization to produce the desired heterocycles.

Photocatalytic pathway: Under visible-light irradiation, g-C_3_N_4_ absorbs photons and promotes electrons from the valence band to the conduction band, generating electron–hole pairs. Their separation and migration can induce oxidation/reduction of adsorbed substrates and generate radical or radical-ion intermediates, which subsequently undergo coupling, cyclization, and product formation. Efficient charge separation is essential for photocatalytic activity, whereas rapid electron–hole recombination can decrease the reaction efficiency.

Radical and oxidative pathway: When supported by appropriate experimental or computational evidence, g-C_3_N_4_-mediated reactions may proceed through photoinduced electron transfer, carbon- or heteroatom-centred radicals, reactive oxygen species, or oxidation by photogenerated holes. These reactive species can participate in C–C, C–N, C–O, or C–S bond formation followed by cyclization. However, radical mechanisms should be considered proposed or plausible when direct mechanistic evidence is unavailable.

Hybrid-catalyst pathway: In modified systems such as Fe_3_O_4_@g-C_3_N_4_ and Pd/g-C_3_N_4_, catalytic enhancement may result from interfacial charge transfer, improved charge separation, substrate adsorption, stabilization of reactive intermediates, or cooperative activation by the incorporated component. Thus, the observed catalytic activity should not necessarily be attributed solely to the g-C_3_N_4_ framework.

Overall, the mechanistic pathways can be summarized as (i) surface acid–base/hydrogen-bond activation, (ii) conventional condensation–addition–cyclization, (iii) visible-light-induced photocatalysis, (iv) radical/oxidative transformation, and (v) hybrid interfacial/cooperative catalysis. This classification provides a clearer distinction between fundamentally different catalytic processes that may otherwise be grouped together simply because they yield similar heterocyclic scaffolds.

## 7. Strategies for Improving Yield in g-C_3_N_4_-Catalysed Heterocyclic Synthesis

The catalytic yield is an important criterion for assessing the effectiveness of g-C_3_N_4_-based catalysts in heterocyclic synthesis. The reported examples indicate that high product yields can be achieved by appropriately controlling catalyst structure, reaction conditions, and activation mode. Catalyst modification is one of the most effective strategies for improving yield. Incorporation of magnetic metal oxides, metal nanoparticles, layered double hydroxides, ionic-liquid functionalities, acidic groups, or other catalytic components can introduce additional active sites, improve substrate adsorption, facilitate interfacial charge separation, or promote activation of specific reaction intermediates. In particular, the combination of g-C_3_N_4_ with metal nanoparticles or metal oxides can enhance substrate activation and catalytic performance.

Reaction conditions also have a decisive influence on product yield. Optimization of catalyst loading, substrate ratio, temperature, solvent, reaction time, and irradiation or mechanical activation can substantially improve conversion and selectivity. Solvent-free conditions and environmentally preferable solvents such as water, ethanol, and aqueous ethanol are particularly attractive when they provide efficient mass transfer and high conversion. In photocatalytic reactions, appropriate light irradiation is required to generate sufficient charge carriers, whereas microwave, ultrasound, and mechanochemical activation can accelerate reaction rates and thereby reduce the time required to achieve high conversion. The present literature also indicates that high yields are frequently associated with short reaction times and low catalyst loading when the catalyst possesses an appropriately engineered surface.

Catalyst morphology is another important factor governing yield. Mesoporosity, nanosheet structures, nanotubular architectures, and high surface accessibility can facilitate contact between the catalyst and reactants. Surface functionalization with acidic, basic, ionic-liquid, or metal-containing groups can further tune the interaction of the catalyst with aldehydes, carbonyl compounds, amines, and other reaction partners. Consequently, rational modification of g-C_3_N_4_ rather than simply increasing the catalyst amount can be a more effective strategy for improving yield.

The examples collected in this review suggest that high yields should not be considered solely because of the intrinsic activity of pristine g-C_3_N_4_. Instead, yield improvement generally results from the combined effects of catalyst composition, surface properties, reaction medium, temperature, reactant concentration and stoichiometry, and external activation. Therefore, systematic optimization and appropriate catalyst design are essential for maximizing both conversion and selectivity in g-C_3_N_4_-catalysed heterocyclic synthesis, as is summarized in Table 5.

## 8. Challenges and Future Perspectives of g-C_3_N_4_-Catalysed MCRs

Although great success has been made in the utilization of g-C_3_N_4_ as a heterogeneous catalyst for multicomponent reactions (MCRs), several drawbacks still exist that limit further development of this catalyst for organic synthesis. In general, bulk g-C_3_N_4_ is characterized by relatively small specific surface area, low accessibility of active sites, and poor electrical conductivity, which might prevent efficient substrate adsorption and catalytic process. In the case of photochemical applications, fast recombination of photoinduced electron–hole pairs reduces quantum efficiency. In addition, catalytic activity of pristine g-C_3_N_4_ is not always enough for carrying out challenging transformations; therefore, structural modification of the material by means of heteroatom doping, defect formation, exfoliation, and hybridization with metal nanoparticles, metal oxides, and carbon-based materials becomes necessary. Although such modifications considerably enhance the catalytic activity, they could increase synthetic complexity and unscalability and destroy the metal-free nature of the catalyst. Moreover, numerous examples of g-C_3_N_4_-catalysed MCRs reported to date were conducted only on a laboratory scale with model substrates, and systematic investigations of reaction mechanism, catalyst stability, scalable synthesis, and industrial application are rather rare [146,147].

The future research needs to be directed toward the rational design of highly efficient g-C_3_N_4_-based catalysts with well-controlled surface chemistry, hierarchical porosity, and a rich number of available active sites. The use of defect-dense, single-atom decorated, and metal-free doped g-C_3_N_4_ catalysts is anticipated to give a higher performance while maintaining sustainability and recyclability. The integration of g-C_3_N_4_ catalysis with novel green techniques such as visible-light photocatalysis, mechanochemistry, microwave, ultrasound-assisted reaction, electrocatalysis, flow synthesis, and green solvents derived from biomass or deep eutectic solvents provides great opportunities for energy-efficient and eco-friendly multicomponent reactions. In addition to that, the combination of experimental studies along with density functional theory (DFT), machine learning, and artificial intelligence-driven catalyst design will provide valuable information about the mechanisms of the reactions and help in discovering efficient catalysts. The use of g-C_3_N_4_ as a catalyst in asymmetric multicomponent reactions, flow synthesis, drug production, and large-scale industry can lead to the use of g-C_3_N_4_ as an effective catalyst for next-generation heterocycle synthesis according to the principles of green chemistry [148,149].

## 9. Conclusions

Graphitic carbon nitride (g-C_3_N_4_) has begun to be one of the most favourable heterogeneous metal-free catalysts for the synthesis of heterocyclic compounds in a sustainable manner. The special characteristic features of this material, which include nitrogen-rich polymeric network structure, excellent thermal and chemical stability, visible-light sensitivity, economical nature, and easy recyclability, make it a highly desirable substitute for traditional homogeneous and transition-metal-based catalysts. As discussed in detail throughout this review article, g-C_3_N_4_ has proved to be a valuable catalyst for thermal, photocatalytic, and multicomponent reactions (MCRs), helping in the preparation of nitrogen-, oxygen-, and sulphur-containing heterocyclic compounds in an environment-friendly manner. The recent advancements made through catalyst modification techniques, which include doping, defect generation, morphology design, and the formation of hybrid composite materials, have resulted in an improvement in the catalytic performance of g-C_3_N_4_. In addition, coupling of g-C_3_N_4_ with green chemistry protocols, which include solvent-free reactions, aqueous phase reactions, visible-light-induced photocatalysis, microwave, and ultrasonic irradiations, clearly highlights the suitability of g-C_3_N_4_ in facilitating sustainable organic synthesis.

However, there are still several issues including surface area limitation, incomplete understanding of the reaction mechanism, and the requirement for consistent green metrics that need to be addressed. For future work, the rational design of novel g-C_3_N_4_-based catalysts and a thorough investigation of their mechanism along with the development of industrially applicable continuous flow procedures are required. In general, the extraordinary flexibility and sustainability of the g-C_3_N_4_ catalyst render it a promising next-generation platform for heterocyclic compound synthesis. Further advancements in the field of catalyst design, mechanistic insights and green process engineering are anticipated to widen the scope of applications and promote sustainable approaches for producing valuable heterocycles.

## Figures and Tables

**Table 1:** Functional roles of individual components in representative g-C_3_N_4_-based catalytic systems.

Catalyst System	Main Components	Role of g-C_3_N_4_	Role of Second Component	Role of Third Component/Modification	Overall Catalytic Function
C_3_N_4_/Fe_3_O_4_/NiFe-LDH	g-C_3_N_4_ + Fe_3_O_4_ + NiFe-LDH	Catalytic support and surface-active framework	Magnetic recovery and interfacial charge/catalytic contribution	Provides additional active hydroxide/metal sites	Synergistic heterogeneous catalysis
PIL-Br-modified tubular g-C_3_N_4_	Tubular g-C_3_N_4_ + PIL-Br	Catalytic/structural semiconductor framework	Modifies surface functionality and substrate interaction	—	Functionalized g-C_3_N_4_ catalysis
Fe_3_O_4_@g-C_3_N_4_-Pr-Cap	Fe_3_O_4_ + g-C_3_N_4_ + Pr-Cap	Support and surface-active framework	Magnetic separation and possible catalytic contribution	Provides functional catalytic sites	Multifunctional heterogeneous catalyst
Fe_3_O_4_@g-C_3_N_4_-SO_3_H	Fe_3_O_4_ + g-C_3_N_4_ + SO_3_H	Support/surface platform	Magnetic recovery	Brønsted-acidic SO_3_H sites	Acid-functionalized heterogeneous catalysis
Cu/Cu_2_O@g-C_3_N_4_	Cu/Cu_2_O + g-C_3_N_4_	Photocatalytic semiconductor and charge-transfer platform	Redox-active Cu/Cu_2_O centres	—	Photoredox catalysis
BC@GCN-P-IL	Biochar + g-C_3_N_4_ + polymeric ionic liquid	Catalytic/photocatalytic framework	Conductive/high-surface-area support	Ionic-liquid functionality modifies surface interactions	Functionalized composite catalysis
g-C_3_N_4_ NRs/Melamine/PMA	g-C_3_N_4_ + melamine + PMA	Main carbon-nitrogen framework	Structural/electronic modification	PMA introduces additional surface functionality	Modified g-C_3_N_4_ catalysis
g-C_3_N_4_/PANI/Cu^2+^	g-C_3_N_4_ + PANI + Cu^2+^	Semiconductor/catalytic platform	Conducting polymer facilitates charge transfer	Cu^2+^ provides redox-active sites	Composite redox/photocatalytic system
CDs/g-C_3_N_4_	Carbon dots + g-C_3_N_4_	Semiconductor framework	Photosensitization/charge-transfer enhancement	—	Enhanced photocatalysis
Cu_2_O/g-C_3_N_4_	Cu_2_O + g-C_3_N_4_	Semiconductor and charge-separation partner	Redox-active semiconductor	—	Heterojunction photocatalysis
g-C_3_N_4_-Bu-SO_3_H	g-C_3_N_4_ + SO_3_H-functionalized group	Heterogeneous framework	Acidic functionality activates suitable substrates	—	Brønsted-acid-assisted catalysis
g-C_3_N_4_/Fe_2_O_3_/ZIF-8	g-C_3_N_4_ + Fe_2_O_3_ + ZIF-8	Semiconductor/support	Redox-active metal oxide	Porous ZIF-8 provides adsorption/dispersion	Multifunctional heterogeneous catalysis
g-C_3_N_4_/NHSO_3_H	g-C_3_N_4_ + sulfamic-acid functionality	Heterogeneous support	Acidic catalytic functionality	—	Acid-functionalized catalysis
g-C_3_N_4_@L-arginine	g-C_3_N_4_ + L-arginine	Heterogeneous support/surface	Provides additional basic/polar functional groups	—	Surface-functionalized catalysis
g-C_3_N_4_/Fe_3_O_4_/CuO	g-C_3_N_4_ + Fe_3_O_4_ + CuO	Semiconductor framework	Magnetic/redox component	CuO provides additional semiconductor/redox sites	Composite photocatalysis
Fe_3_O_4_@g-C_3_N_4_@Thiamine	Fe_3_O_4_ + g-C_3_N_4_ + thiamine	Heterogeneous framework	Magnetic separation	Thiamine introduces functional catalytic sites	Functionalized recyclable catalyst
W/Cu@g-C_3_N_4_	W/Cu + g-C_3_N_4_	Support/photocatalytic platform	Metal centres provide catalytic/redox sites	—	Multifunctional catalysis
Cu_2_O@g-C_3_N_4_	Cu_2_O + g-C_3_N_4_	Semiconductor support/charge-transfer partner	Cu_2_O provides redox-active sites	—	Composite catalysis
AgNPs@g-C_3_N_4_	Ag nanoparticles + g-C_3_N_4_	Semiconductor support	Ag provides catalytic/electron-trapping sites	—	Heterogeneous/photocatalytic catalysis
Ag/g-C_3_N_4_	Ag + g-C_3_N_4_	Catalytic/photocatalytic framework	Ag provides active metal sites and facilitates electron transfer	—	Composite catalysis
g-C_3_N_4_/CS/MOF-5	g-C_3_N_4_ + chitosan + MOF-5	Catalytic/photocatalytic component	Chitosan provides functional groups	MOF-5 contributes porosity and adsorption	Multifunctional porous composite

**Table 2:** Preparation and structural modification of g-C_3_N_4_-based catalysts used in the reported reactions.

Scheme	Catalyst	Preparation Method Explicitly Stated in Manuscript	Subsequent Modification/Composite Formation	Morphology/Structural Feature
1	C_3_N_4_/Fe_3_O_4_/NiFe-LDH	Nanocomposite preparation	Fe_3_O_4_ + NiFe-LDH	Magnetic mesoporous
2	PIL-Br-modified tubular g-C_3_N_4_ (CNT1)	Hollow tubular g-C_3_N_4_ fabrication followed by PIL-Br modification	Poly(ionic liquid) bromide	Hollow tubular/nanoporous
3	Fe_3_O_4_@g-C_3_N_4_-Pr-Cap	Magnetic g-C_3_N_4_ followed by linker-assisted functionalization	Fe_3_O_4_ + captopril + linker	Magnetic functionalized
4	Fe_3_O_4_@g-C_3_N_4_-SO_3_H	g-C_3_N_4_ composite preparation and surface functionalization	Fe_3_O_4_ + SO_3_H	Magnetic acidic catalyst
5	Cu/Cu_2_O@g-C_3_N_4_	g-C_3_N_4_/Cu-Cu_2_O nanocomposite preparation	Cu/Cu_2_O	Photocatalytic nanocomposite
6	BC@g-C_3_N_4_-P-IL	Calcination, followed by functionalization	Acidic and ionic-liquid groups	Biochar-based GCN
7	g-C_3_N_4_ NRs/Melamine/PMA	GCN nanorod preparation followed by PMA modification	PMA	Nanorods
9	g-C_3_N_4_/PANI/Cu^2+^	Composite preparation	PANI + Cu^2+^	Polymer/metal-functionalized
10	CDs/g-C_3_N_4_	CDs/g-C_3_N_4_ heterojunction preparation	Carbon dots	Heterojunction
13	MSN/C_3_N_4_/CNH	Mesoporous silica/GCN/CNH composite preparation	MSN + CNH	Mesoporous composite
14	g-C_3_N_4_[IMEP][Cl]/CNT	CNT fabricated using ionic copolymer template	[IMEP][Cl] copolymer	Mesoporous carbon nitride tube
15	g-C_3_N_4_/Fe_2_O_3_/ZIF-8	Straightforward synthetic approach	Fe_2_O_3_ + ZIF-8	Nanocomposite
16	g-C_3_N_4_-CO-(CH_2_)_3_-CO_2_H	GCN preparation followed by sequential functionalization	Glutaric anhydride + 1,3-propanesultone	Surface-functionalized GCN
17	g-C_3_N_4_/NHSO_3_H	Covalent sulfamic-acid functionalization	NHSO_3_H	Acid-functionalized
18	g-C_3_N_4_@Bu-SO_3_H	GCN nanosheet functionalization	1,4-Butanesultone/SO_3_H	Functionalized nanosheets
19	CNCT	Exfoliation of g-C_3_N_4_ followed by Cu_3_TiO_4_ incorporation	Cu_3_TiO_4_	Exfoliated nanosheet composite
20	g-C_3_N_4_@L-arginine	GCN-supported L-arginine preparation	L-Arginine	Functionalized GCN
22	CN-Pr-VB1	Covalent functionalization of GCN nanosheets	1,3-Dibromopropane + vitamin B1	GCN nanosheets
28	Bi/Cr-ZnO@g-C_3_N_4_	Multicomponent nanocomposite preparation	4% Bi + 1% Cr-doped ZnO	Doped ZnO/GCN
29	Co_3_O_4_/g-C_3_N_4_Si	Nanocomposite preparation	Co_3_O_4_ + Si-containing GCN	Nanocomposite
30	Lysozyme@g-C_3_N_4_	Immobilization of lysozyme on GCN	Lysozyme	Biocatalyst/GCN
32	CuFe_2_O_4_/g-C_3_N_4_	Facile one-step method	CuFe_2_O_4_	Magnetic hybrid
33	Fe_3_O_4_-g-C_3_N_4_-alginate-Ag	Multicomponent nanocomposite preparation	Fe_3_O_4_ + alginate + Ag NPs	Magnetic Ag composite
39	Hal-BS/g-C_3_N_4_-Dy	Functionalized GCN/halloysite composite preparation	Halloysite + Dy-balsalazide complex	Functionalized porous composite
42	g-C_3_N_4_/Fe_3_O_4_/CuO	Composite preparation	Fe_3_O_4_ + CuO	Magnetic photocatalyst
44	g-C_3_N_4_/Fe_3_O_4_@P_2_W_15_V_3_	Decoration of GCN with magnetic heteropolyacid	Fe_3_O_4_ + P_2_W_15_V_3_	Magnetic nanocomposite
45	Fe(III)@g-C_3_N_4_	FeCl_3_ impregnation onto GCN	Fe(III)	Metal-supported GCN
47	Fe_3_O_4_@g-C_3_N_4_@Thiamine	Functionalization using TCT linker	Thiamine hydrochloride + cyanuric chloride	Magnetic organocatalyst
49	SbCl_3_@Fe_3_O_4_/g-C_3_N_4_	Simple solvent-evaporation method	Fe_3_O_4_ + SbCl_3_	Magnetic metal-functionalized
51	W/Cu@g-C_3_N_4_	Hydrothermal method	WCl_6_ + CuCl_2_	Metal-loaded GCN
57	g-C_3_N_4_/amidosulfonic acid	In situ immobilization of NH_2_SO_3_H under hydrothermal conditions	Amidosulfonic acid	Acid-functionalized
60	g-C_3_N_4_/CS/MOF-5	Nanocomposite preparation	Chitosan + MOF-5	Biocompatible porous composite
61	MgAl_2_O_4_/S-g-C_3_N_4_	Incorporation of MgAl_2_O_4_ onto S-doped GCN nanosheets	MgAl_2_O_4_ + S doping	Composite nanosheets

**Table 3:** Important green metrics for g-C_3_N_4_-catalysed MCRs.

Green Metrics	Definition	Significance in g-C_3_N_4_ MCRs
Atom Economy (AE)	Percentage of reactant atoms incorporated into the final product	MCRs generally exhibit 80–100% AE because most atoms are retained in the heterocyclic framework.
Reaction Mass Efficiency (RME)	Isolated product mass relative to the combined reactant mass	High isolated yields (85–98%) provide excellent RME.
E-factor	Amount of waste produced per unit mass of product	Low E-factor due to one-pot synthesis, minimal purification, and reusable catalyst.
Process Mass Intensity (PMI)	Mass of materials consumed per unit mass of product	Reduced because of fewer reaction steps and low solvent consumption.
Carbon Efficiency (CE)	Percentage of carbon atoms incorporated into the desired product	High owing to efficient multicomponent condensation.
Catalyst Recyclability	Number of successful reuse cycles without significant activity loss	Most g-C_3_N_4_ catalysts remain active for 3–10 cycles with negligible decrease in yield.
Energy Efficiency	Energy consumed during synthesis	Enhanced by room-temperature, microwave, visible-light, mechanochemical, or ultrasonic protocols.
Turnover Number (TON) & Turnover Frequency (TOF)	Catalyst productivity and catalytic activity	Useful for comparing pristine and modified g-C_3_N_4_ catalysts.

**Table 4:** Green metrics used to evaluate the sustainability of g-C_3_N_4_-catalysed reactions.

Ref.	Heterocyclic Product	Catalyst	Catalyst Loading	Solvent/Medium	Temperature/Irradiation	Time	Yield	Reuse
[36]	DHPMs	C_3_N_4_/Fe_3_O_4_/NiFe-LDH	15 mg	Solvent-free	80 °C	5–15 min	91–96%	5 cycles
[37]	Imidazo-pyrimidines	CNT1 (PIL-Br modified g-C_3_N_4_)	10 mg	H_2_O	Ultrasonication	3–15 min	96–98%	6 cycles
[40]	1,4-DHPs	Cu/Cu_2_O@g-C_3_N_4_	10 mg	EtOH	White LED, 5 W	40–55 min	85–96%	5 cycles
[48]	O-Arylated quinolines	CDs/g-C_3_N_4_	9.6 mg	DMF	Blue LED	12–24 h	65–90%	5 cycles
[51]	PHQs	MSN/C_3_N_4_/CNH	10 mg	Solvent-free	50 °C	15 min	88–97%	6 cycles
[52]	Hexahydroquinolines	g-C_3_N_4_ [IMEP][Cl]	10 mg	H_2_O	Ultrasonication	5 min	93–98%	7 cycles
[65]	Quinoxalines	g-C_3_N_4_-Pr-VB1	15 mg	Solvent-free	100 °C	3–40 min	81–97%	6 cycles
[69]	Imidazoles	Fe_3_O_4_@g-C_3_N_4_	20 mg	EtOH	78 °C	120–200 min	72–95%	6 cycles
[73]	Imidazoles	CuO@g-C_3_N_4_	12 mg	EtOH	Reflux/ultrasound	2–4 h	72–85%	5 cycles
[82]	Triazoles	Fe_3_O_4_-g-C_3_N_4_-alginate-Ag	0.02 g	H_2_O	Room temp.	15 min	90–98%	7 cycles
[85]	Tetrazoles	Pd@g-C_3_N_4_	20 mg	PEG-400	100 °C	40 min–3 h	83–98%	5 cycles
[93]	Annulated chromenes	Hal-BS/g-C_3_N_4_-Dy	40 mg	H_2_O	Reflux	11–30 min	90–98%	8 cycles
[98]	Pyrano-pyrimidines	g-C_3_N_4_	0.06 g	EtOH	Room temp.	40–65 min	80–96%	5 cycles
[99]	Pyrans	g-C_3_N_4_/Fe_3_O_4_/CuO	30 mg	H_2_O	Visible light/reflux	4–8 min	78–89%	5 cycles
[107]	Spirooxindole-pyrans	Fe_3_O_4_@g-C_3_N_4_@Thiamine	25 mg	Solvent-free	—	5 min	92–98%	7 cycles
[111]	Imidazo-pyridines	SbCl_3_@Fe_3_O_4_/g-C_3_N_4_	25 mg	Ethylene glycol	Room temp.	2–4 h	72–95%	5 cycles
[123]	Thiazolidinones	Ag/g-C_3_N_4_	25 mg	EtOH/H_2_O (2:1)	Ultrasound	20 min	95–97%	5 cycles
[127]	Pyrroles	g-C_3_N_4_/amidosulfonic acid	20 mg	EtOH	Room temp.	100–190 min	69–98%	5 cycles
[133]	Acridines	g-C_3_N_4_@L-arginine	20 mg	EtOH	80 °C	20 min	90–99%	5 cycles
[136]	Pyrano-phenazines	g-C_3_N_4_/CS/MOF-5	0.015 g	EtOH/H_2_O	90 °C	30–120 min	77–96%	6 cycles
[139]	Epoxides	MgAl_2_O_4_/S-g-C_3_N_4_	20 mg	EtOH/H_2_O_2_	Ultrasound	2–3 h	39–94%	5 cycles
[142]	α-Aminonitriles	Fe_3_O_4_/g-C_3_N_4_	50 mg	EtOH	70 °C	2–4 h	78–95%	5 cycles

**Table 5:** Summary of factors for improving yield.

Factor	Effect on Yield	Strategy Highlighted in the Review
Catalyst composition	Provides additional active sites and improved substrate activation	Combine g-C_3_N_4_ with Fe_3_O_4_, metal oxides, metals, LDHs, etc.
Surface functionalization	Tunes acidic/basic interactions and substrate adsorption	SO_3_H, ionic liquids, amino acids, organic functionalities
Morphology	Improves surface accessibility and mass transfer	Mesoporous, nanosheet, nanotubular structures
Catalyst loading	Adequate loading increases active-site availability; excessive loading may be unnecessary	Optimize catalyst amount rather than simply increasing it
Temperature	Increases reaction rate when thermally activated	Optimize temperature according to substrate/catalyst
Solvent	Influences solubility, mass transfer and catalyst–substrate interactions	Solvent-free, water, ethanol, aqueous ethanol where appropriate
Reactant ratio	Controls conversion and suppresses incomplete reaction	Optimize stoichiometric ratio of MCR components
Reaction time	Sufficient time improves conversion; excessive time is inefficient	Optimize to achieve maximum yield in minimum time
Light irradiation	Generates photocarriers in photocatalytic systems	Optimize wavelength/intensity and irradiation conditions
Ultrasound/microwave/ mechanochemistry	Enhances mixing/activation and can shorten reaction time	Use appropriate external activation
Metal/semiconductor coupling	Can improve charge separation and substrate activation	Pd, Cu, Fe-based and metal-oxide/GCN systems
Recyclability	Maintains catalytic performance over repeated cycles	Magnetic/separable g-C_3_N_4_ composites

## Data Availability

No new data were created or analyzed in this study. Data sharing is not applicable to this article.
